# Ubiquilins Chaperone and Triage Mitochondrial Membrane Proteins for Degradation

**DOI:** 10.1016/j.molcel.2016.05.020

**Published:** 2016-07-07

**Authors:** Eisuke Itakura, Eszter Zavodszky, Sichen Shao, Matthew L. Wohlever, Robert J. Keenan, Ramanujan S. Hegde

**Affiliations:** 1 MRC Laboratory of Molecular Biology, Francis Crick Avenue, Cambridge CB2 0QH, UK; 2 Department of Biology, Faculty of Science, Chiba University, 1-33, Yayoi-cho, Inage-ku, Chiba, 263-8522, Japan; 3 Department of Biochemistry and Molecular Biology, The University of Chicago, 929 East 57^th^ Street, Chicago, IL 60637, USA

## Abstract

We investigated how mitochondrial membrane proteins remain soluble in the cytosol until their delivery to mitochondria or degradation at the proteasome. We show that Ubiquilin family proteins bind transmembrane domains in the cytosol to prevent aggregation and temporarily allow opportunities for membrane targeting. Over time, Ubiquilins recruit an E3 ligase to ubiquitinate bound clients. The attached ubiquitin engages Ubiquilin’s UBA domain, normally bound to an intramolecular UBL domain, and stabilizes the Ubiquilin-client complex. This conformational change precludes additional chances at membrane targeting for the client, while simultaneously freeing Ubiquilin’s UBL domain for targeting to the proteasome. Loss of Ubiquilins by genetic ablation or sequestration in polyglutamine aggregates leads to accumulation of non-inserted mitochondrial membrane protein precursors. These findings define Ubiquilins as a family of chaperones for cytosolically exposed transmembrane domains and explain how they use ubiquitin to triage clients for degradation via coordinated intra- and intermolecular interactions.

## Highlights

• Ubiquilins are conserved cytosolic chaperones for transmembrane domains
• Mitochondrial membrane proteins that fail targeting are degraded by Ubiquilins
• The absence of Ubiquilins in cells leads to membrane protein precursor aggregation
• Interactions with Ubiquilins’ UBA and UBL domains coordinate client degradation

## Graphical Abstract

**Figure:**
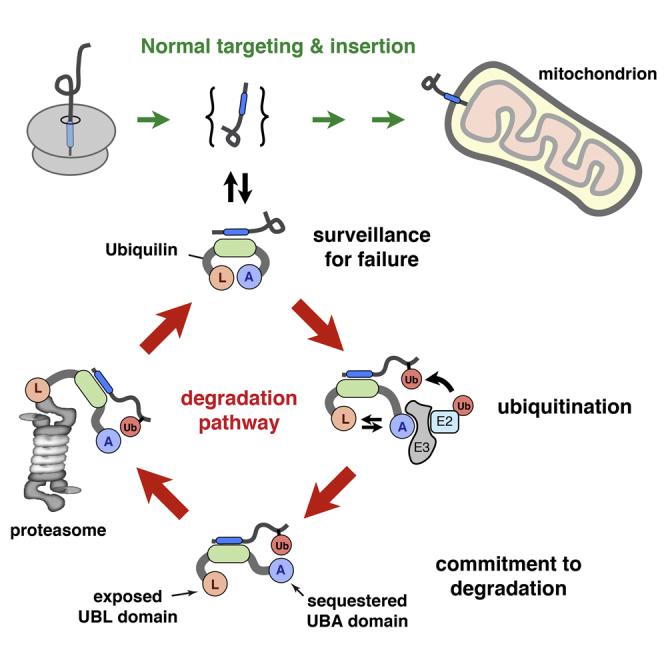

## Teaser

Membrane proteins require targeting to an intracellular organelle for insertion. When targeting fails, they must be recognized and degraded promptly to avoid aggregation. Itakura et al. show that Ubiquilins are cytosolic chaperones that prevent aggregation of mitochondrial membrane protein precursors, routing them for degradation if they linger uninserted in the cytosol.

## Introduction

Approximately 25% of protein-coding genes in all organisms encode integral membrane proteins. Although their final destination is in a lipid bilayer, they are synthesized by cytosolic machinery and transiently navigate the aqueous cytosol. Because the transmembrane domains (TMDs) in these proteins are effectively insoluble in aqueous environments, specialized machinery must recognize and shield them until their insertion. Several TMD-shielding factors have been identified and extensively studied for proteins destined for the endoplasmic reticulum (ER) (Cross et al., 2009, Hegde and Keenan, 2011). In contrast, the factors that interact with and maintain the solubility of mitochondrial membrane proteins during their transient residence in the cytosol are incompletely defined.

Mitochondria contain ∼1,000–1,500 proteins. Nearly all mitochondrial proteins are imported from the cytosol (Neupert, 1997), and a large proportion of them contain TMDs. The import machinery at the outer and inner mitochondrial membranes has been extensively studied (Chacinska et al., 2009). However, the cytosolic factors that maintain import competence of mitochondrial precursors, prevent aggregation, and route them for degradation in the case of import failure are largely unknown. The general chaperones Hsp70 and Hsp90 are implicated in maintaining an unfolded state and targeting various precursors to the mitochondrial outer membrane (Young et al., 2003). However, these chaperones have client-binding clefts that are too small to effectively shield the long hydrophobic regions that typify many TMDs (Shiau et al., 2006, Zhu et al., 1996), and suitable alternative factors have not been identified.

TMD shielding is likely to be important not only for productive biogenesis, but also for degradation of failed insertion precursors. Failure of mitochondrial import can occur for a number of reasons. In addition to inherent inefficiencies that accompany any biological process (Wolff et al., 2014), import is under regulatory control and may be acutely inhibited under different physiologic conditions (Harbauer et al., 2014, Schmidt et al., 2011). Furthermore, mitochondrial stress can result in impaired import (Wright et al., 2001), and chronic failure of import is detrimental to cytosolic protein homeostasis (Wang and Chen, 2015, Wrobel et al., 2015). Thus, cells presumably have pathways to degrade mitochondrial precursors after their initial attempts at translocation or insertion fail. The factors involved in this type of quality control are not clear. As with biogenesis, membrane proteins pose a particularly difficult challenge due to their relative insolubility.

Nearly all available information about chaperones for TMDs in the cytosol comes from the study of targeting and degradation of proteins destined for the ER (Cross et al., 2009, Hegde and Keenan, 2011). Most ER membrane proteins are recognized co-translationally by the ribosome-associating signal recognition particle (SRP). The positioning of its TMD-binding domain at the ribosome exit tunnel ensures effective TMD shielding from solvent during targeting. Membrane proteins that fail to engage SRP encounter a different set of cytosolic TMD-binding factors that mediate post-translational targeting to the ER (Hegde and Keenan, 2011) or degradation at the proteasome (Hessa et al., 2011).

The major class of membrane proteins post-translationally targeted to the ER are tail-anchored (TA) proteins, named for their single C-terminal TMD (Hegde and Keenan, 2011). TA proteins must be loaded onto an ATPase named Get3 (TRC40 in mammals) for targeting to ER-resident receptors (Stefanovic and Hegde, 2007, Schuldiner et al., 2008). Loading onto Get3 requires transfer, via a bridging complex, from a “pre-targeting” chaperone called Sgt2 (Wang et al., 2010). Mammals appear to use homologous components, with the additional inclusion of Bag6 as a subunit of the bridging complex (Mariappan et al., 2010). Sgt2 (SGTA in mammals), Get3, and Bag6 are all TMD-binding proteins, thereby precluding aggregation of their hydrophobic clients. Of these, Bag6 appears to have broader specificity beyond TA proteins (Hessa et al., 2011) and can route its clients for degradation via recruitment of an E3 ubiquitin ligase (Rodrigo-Brenni et al., 2014).

Given the extensive machinery for ER membrane proteins, we anticipated the existence of analogous factors that operate on the equally challenging problem of mitochondrial membrane proteins. However, earlier searches for the simplified case of mitochondrial TA proteins (e.g., Krumpe et al., 2012, Setoguchi et al., 2006) did not reveal protein factors in either the cytosol or the membrane required for targeting, insertion, or degradation. We now report that Ubiquilins are general TMD chaperones for both TA and non-TA mitochondrial membrane proteins. Functional and biochemical studies indicate that the primary role of Ubiquilins is to triage their clients for proteasomal degradation in the instance of failed membrane targeting.

## Results

### Ubiquilins Are Major TMD-Binding Proteins of the Cytosol

**Figure 1:**
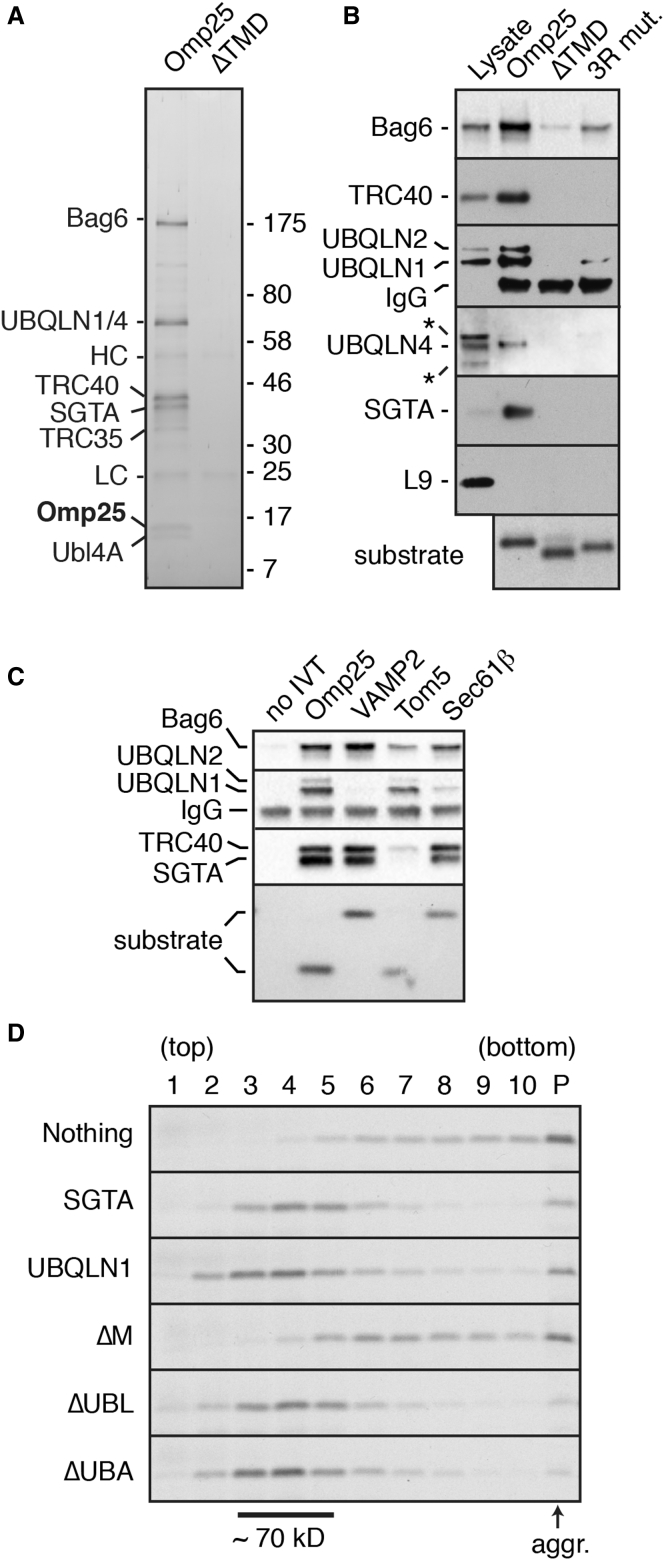
Ubiquilins Are Cytosolic TMD-Binding Chaperones (A) FLAG-tagged constructs containing or lacking the Omp25 TMD (see Figure S1) were translated in RRL, affinity purified via the FLAG tag, separated by SDS-PAGE, and detected with SYPRO-Ruby stain. The identity of each band is indicated. HC and LC are immunoglobulin heavy and light chain, respectively. (B) FLAG-tagged constructs containing the Omp25 TMD or the indicated TMD mutants were translated in RRL containing ^35^S-methionine, affinity purified, and analyzed by immunoblotting relative to total RRL (first lane). The translated substrates were detected using autoradiography. Asterisks indicate non-specific products. (C) FLAG-tagged constructs containing the TMD regions of the indicated proteins were analyzed for interaction partners as in (B). (D) FLAG-tagged construct containing the Omp25 TMD was translated in the PURE translation system with ^35^S-methionine and 15 μM of recombinant SGTA, UBQLN1, or the indicated UBQLN1 mutant. The reactions were separated by sucrose gradient, and the Omp25 visualized by autoradiography. SGTA (∼70 kD native size) and each of the recombinant UBQLN1 proteins migrate primarily in fractions 3 to 5 (not shown). See also Figures S1 and S2.

We initiated our studies using the outer mitochondrial TA membrane protein Omp25 (Figure S1A). We found that Omp25 synthesized in rabbit reticulocyte lysate (RRL) can be selectively targeted to mitochondria of semi-permeabilized cells (Figure S1B), but is ubiquitinated when insertion is precluded (Figure S1C). Both targeting and ubiquitination strictly depend on its TMD, which was observed to engage with factors in the translation extract as judged by the relatively large native size of Omp25 on sucrose gradient separations (Figure S1D). To find these putative factor(s), we affinity purified RRL-translated FLAG-tagged Omp25 and identified the major interaction partners using mass spectrometry (Figure 1A). In addition to the TMD chaperones for ER-destined TA proteins (SGTA, TRC40, and the heterotrimeric Bag6 complex), we identified UBQLN1 and UBQLN4, two homologous members of the Ubiquilin (UBQLN) family of proteins.

Mammals have four UBQLNs with identical domain architecture and high sequence conservation (Figure S2A). They have an N-terminal ubiquitin-like (UBL) domain and a C-terminal ubiquitin-associating (UBA) domain. The middle region (hereafter termed the M domain) is poorly characterized, but has an unusually high methionine content similar to the TMD-binding domains of SRP (Bernstein et al., 1989), SGTA (Wang et al., 2010), and TRC40 (Mateja et al., 2009). This region contains predicted “Sti1-like” domains of uncertain relevance. Three of the UBQLNs (1, 2, and 4) are widely expressed, show between 68% and 83% sequence similarity to each other, and are specifically recovered in Omp25 immunoprecipitates (IPs) in a TMD-dependent (Figure 1B) and detergent-sensitive (data not shown) manner. The more distantly related testis-specific UBQLN3 was not identified with mass spectrometry or IP and was not pursued further.

Analysis of the interaction partners for a range of TMDs and mutants indicate that UBQLNs prefer moderately hydrophobic TMDs that typify many mitochondrial membrane proteins (Figures 1C and S2B). Hence, TMDs from the mitochondrial proteins Omp25, Tom5, and Bak engage UBQLNs more effectively than the ER-destined TMDs from Sec61β and VAMP2. ATP5G1, a two-TMD inner mitochondrial membrane protein containing a mitochondrial targeting signal (MTS) for import via the TOM and TIM complexes (Figure S2C; Chacinska et al., 2009), also interacted with UBQLNs in a TMD-dependent manner (Figure S2D). No interaction was seen with either the MTS or incomplete or disrupted TMDs. At this level of resolution, we have not observed major differences in the substrate specificity of the different UBQLN family members, so we focused our subsequent studies on UBQLN1.

To determine whether UBQLN1 can maintain solubility of its TMD-containing client, we used the “PURE” translation system reconstituted with recombinant *E*. *coli* translation factors (Shimizu and Ueda, 2010). Aggregation of newly synthesized Omp25 in this chaperone-free system was substantially prevented by the presence of recombinant UBQLN1 (Figure 1D). Analysis of UBQLN1 deletion mutants in this assay indicates that the UBA and UBL domains are dispensable for TMD binding, which is mediated by the central M domain (Figure 1D). The native size of the UBQLN1-Omp25 complex suggests that it contains a single UBQLN1, similar to the native UBQLN-Omp25 complexes observed in cytosol (Figure S2E).

Depletion of the major TMD-binding proteins from RRL by passage over phenyl-sepharose produced a translation extract with diminished capacity to maintain Omp25 solubility (Figure S2E). This was rescued in a dose-dependent manner by UBQLN1. The major cytosolic chaperones of the Hsp70 and Hsp90 family are not depleted by phenyl-sepharose. Therefore, whereas these general chaperones appear to maintain translocation competence of less hydrophobic protein precursors (Young et al., 2003), hydrophobic TMD-containing proteins require other factor(s) to prevent their aggregation. UBQLNs appear to be a highly conserved, widely expressed, and abundant family of factors that are capable of fulfilling this TMD-chaperoning function in vitro.

### UBQLN Insufficiency Causes Aggregation of Membrane Protein Precursors

**Figure 2:**
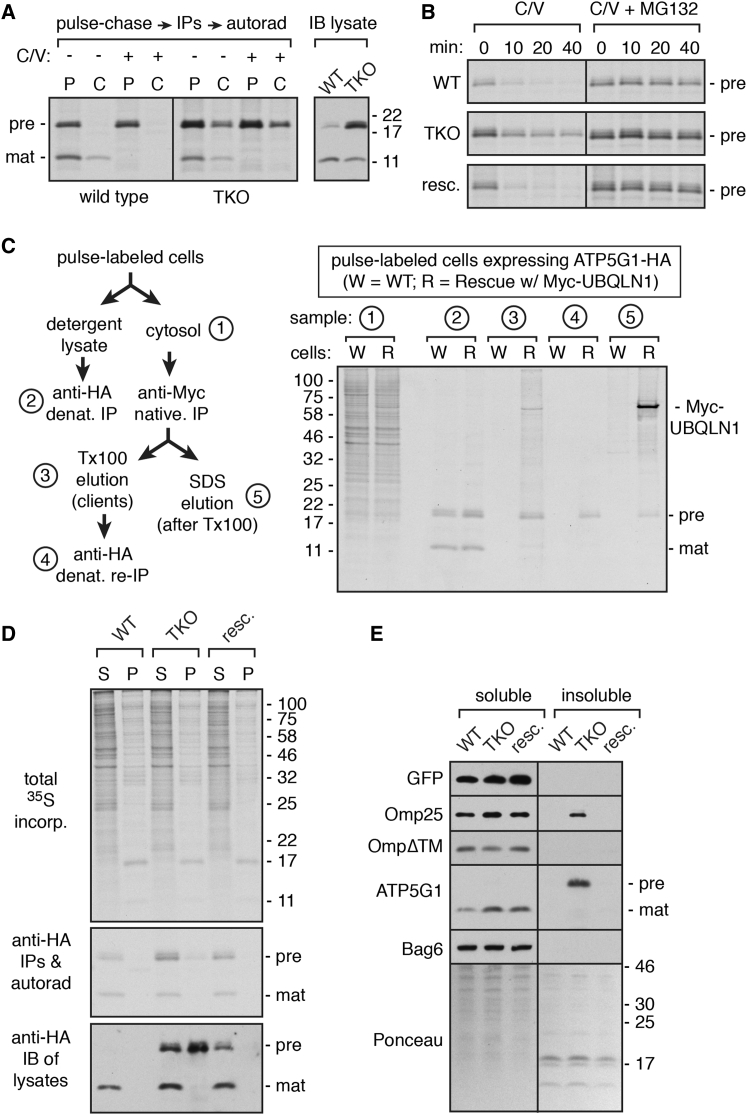
The Consequences of UBQLN Deficiency in Cells (A) Wild-type (WT) or UBQLN triple knockout (TKO) cells expressing ATP5G1-HA were pulse-labeled with ^35^S-methionine for 30 min and chased for 1 hr with unlabeled methionine. Immunoprecipitated ATP5G1-HA was detected using autoradiography. Where indicated, a mixture of CCCP and valinomycin (C/V) was included during the pulse-chase to inhibit mitochondrial import. The right image shows an anti-HA immunoblot of untreated total cell lysate to detect steady-state levels of ATP5G1-HA. The positions of precursor (pre) and mature (mat) forms of ATP5G1 are indicated. (B) WT, TKO, and Myc-UBQLN1 rescue cells (resc.) were transfected with ATP5G1-HA and analyzed by pulse-chase labeling in the presence of C/V. Pulse time was 5 min, and chase times were from 0 to 40 min. Where indicated, the proteasome was inhibited with MG132. (C) Detergent-free cytosolic lysates from cells pulse-labeled for 30 min with ^35^S-methionine were subjected to the separation protocol shown on the left. The indicated fractions were analyzed by SDS-PAGE and autoradiography. The positions of Myc-UBQLN1 (only expressed in the rescue cells) and the precursor and mature forms of ATP5G1-HA (expressed in both cells) are indicated. (D) WT, TKO, and rescue cells transfected with ATP5G1-HA were pulse-labeled for 30 min with ^35^S-methionine, separated into detergent soluble (S) and insoluble (P) fractions, and either analyzed directly by autoradiography (top), immunoblotting with anti-HA (bottom), or immunoprecipitation with anti-HA and autoradiography (middle). (E) WT, TKO, and rescue cells were transfected with plasmids expressing GFP, GFP-Omp25, GFP-Omp25ΔTM, or ATP5G1-HA, separated into detergent-soluble and insoluble fractions, and equivalent amounts of each fraction immunoblotted for the respective antigens. Endogenous Bag6 and the Ponceau-stained blot from one of the experiments are shown to illustrate uniformity of fractionation. The fractions for any given sample were analyzed on the same gel, and the image was assembled from the same exposure in each case. See also Figures S3 and S4.

To understand the physiologic importance of this biochemical activity, we simultaneously disrupted UBQLN1, 2, and 4 in cultured cells using CRISPR/Cas9 methodology (Figure S3A) and analyzed the effect on transfected mitochondrial membrane proteins. We focused on ATP5G1 because its cytosolic precursor and mitochondrial mature product can be distinguished by import-dependent removal of its MTS (Figure S2C). Pulse-labeling of wild-type (WT) cells transfected with ATP5G1-HA showed both precursor and mature products. After a 1 hr chase, only the mature product persisted (Figure 2A). The lower levels of mature product after chase is likely due to degradation within mitochondria after failing to incorporate into pre-existing ATP synthase (Koppen and Langer, 2007). When mitochondrial import is inhibited with CCCP and valinomycin, only ATP5G1-HA precursor is observed after the pulse, and is completely degraded during the chase.

In UBQLN1/2/4 triple knockout (TKO) cells, we observed essentially identical amounts of mature ATP5G1-HA compared to WT cells (Figure 2A). In contrast, the amount of precursor was substantially (∼3-fold) higher after the pulse and was incompletely degraded during the chase. Similar precursor stabilization preferentially in TKO cells was seen in the presence of CCCP and valinomycin (Figure 2A) and at a wide range of ATP5G1-HA expression levels (Figures S3B and S3C). This suggested that the absence of UBQLNs has little effect on ATP5G1-HA import, but significantly impairs the degradation of non-imported precursors. Indeed, immunoblotting of total cell lysate showed similar steady-state levels of mature ATP5G1-HA in WT and TKO cells, but increased precursor selectively in the latter (Figure 2A).

Finer pulse-chase studies under conditions of blocked mitochondrial import (Figure 2B) showed that degradation of non-imported precursor in WT cells was rapid and nearly complete within 20 min. In contrast, ∼50% precursor remained in TKO cells after 40 min, and this stabilization was rescued by re-expressing physiologic levels of Myc-UBQLN1 (hereafter termed rescue cells; Figure S3A). In all cases, degradation was completely blocked by proteasome inhibition. Pulse-chase studies without and with proteasome inhibition in WT cells indicate that ∼25% of nascent ATP5G1-HA molecules fail import (Figure S3D). Although this is exaggerated by overexpression (e.g., Figure S3C), import failure would always occur at some basal level, necessitating the UBQLN-dependent degradation pathway.

A role for UBQLNs in precursor degradation in cells was further supported by an interaction between newly synthesized ATP5G1-HA and Myc-UBQLN1 in rescue cells by co-immunoprecipitation (Figure 2C). This interaction was detergent sensitive, permitting selective release of ATPG1-HA from immunoprecipitated Myc-UBQLN1. Numerous nascent endogenous interacting partners (observed as a heterogeneous collection of products of varying sizes) were also eluted from Myc-UBQLN1 under these conditions, indicating their binding via primarily hydrophobic interactions. This is further consistent with our conclusion, based on in vitro analysis, that UBQLNs engage clients via TMDs.

At steady state, ATP5G1-HA precursors that fail to be degraded in TKO cells were mostly detergent insoluble (>70%), consistent with their aggregation (Figure 2D, bottom image). However, this aggregation was minimal (<10%) for newly synthesized ATP5G1-HA detected using pulse labeling (Figure 2D, middle image). This suggests that other chaperones, such as perhaps SGTA (e.g., Figure 1D), can temporarily maintain precursor solubility, but that the inability to degrade this product leads to its aggregation over time. Similar to ATP5G1, we observed increased detergent-insoluble Omp25 selectively in TKO cells (Figure 2E), without any appreciable difference in the amount of Omp25 successfully targeted to mitochondria (Figure S3E). As expected from in vitro interaction results (Figure 1B), UBQLN-dependent changes in Omp25 solubility were TMD dependent (Figure 2E).

Thus, for both a TA protein (Omp25) and a TOM/TIM pathway client (ATP5G1), UBQLNs are crucial for preventing aggregation and promoting efficient degradation of non-imported mitochondrial products. At this level of resolution, we have not detected a consistent effect on insertion, and do not observe any obvious alterations in mitochondrial morphology (Figure S3E), induction of mitochondrial stress (Figure S4A), or reduced levels of respiratory chain complexes (Figure S4A) in TKO cells. This suggests that other TMD-binding partners (e.g., Figure 1A), perhaps aided by an activated heat shock response (Figures S4A and S4B), prevent aggregation over short time frames to permit membrane targeting and maintain mitochondrial biogenesis. In contrast, cytosolic precursor stabilization and aggregation, a constitutive heat shock response, and decreased fitness in TKO cells (Figures S4C and S4D) all point to the physiologic importance of UBQLNs in maintaining cytosolic protein homeostasis.

### Pathologic UBQLN Depletion by Polyglutamine Aggregates

Earlier studies had suggested that aggregates of expanded polyglutamine (polyQ)-containing proteins, implicated in a variety of human diseases, may partially sequester UBQLNs (Doi et al., 2004, Wang and Monteiro, 2007, Mori et al., 2012, Rutherford et al., 2013). This raised the possibility that polyQ aggregates might affect cytosolic protein homeostasis by depleting UBQLNs. We tested this idea by developing a single-cell fluorescent reporter of UBQLN-mediated degradation and analyzing whether this reporter was preferentially stabilized in polyQ aggregate-containing cells.

**Figure 3:**
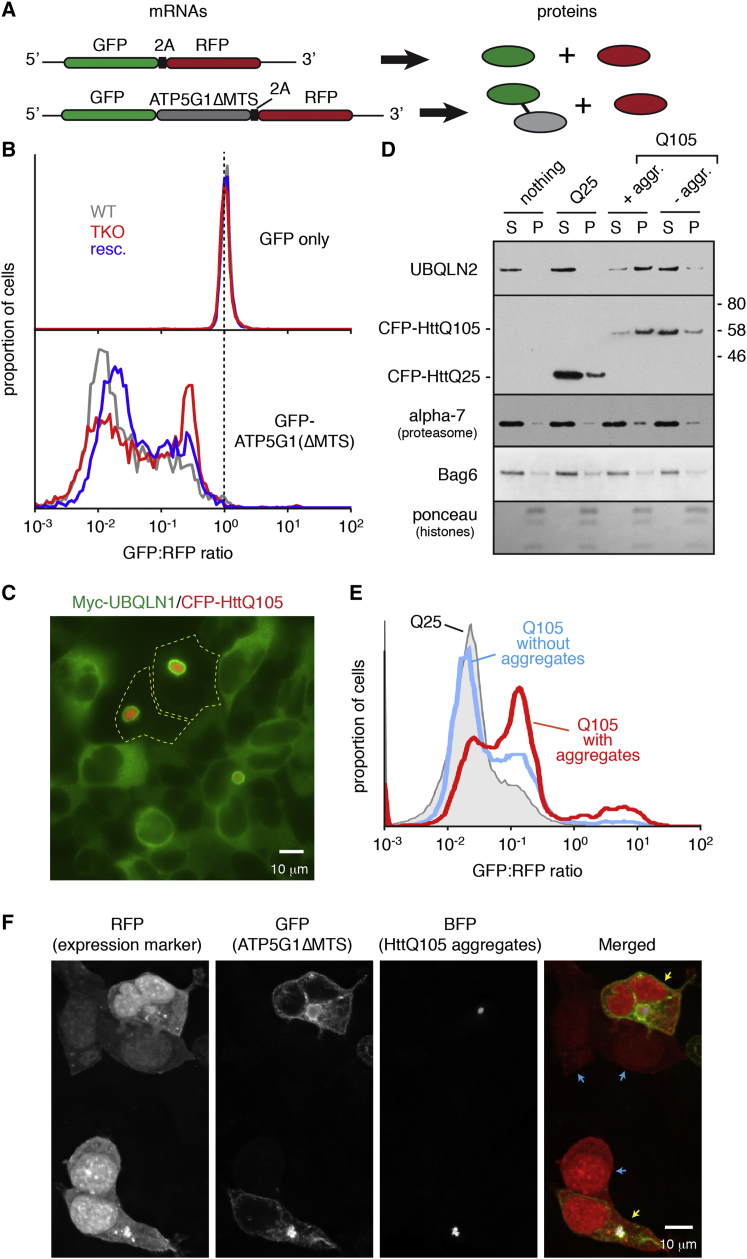
Pathologic UBQLN Depletion by PolyQ Aggregates (A) Schematic of the expected mRNA and protein products of GFP-2A-RFP constructs. (B) Flow cytometry analysis of the GFP:RFP ratio of the indicated cell lines expressing GFP-2A-RFP (top) or GFP-ATP5G1ΔMTS-2A-RFP (bottom). (C) TKO cells rescued with Myc-UBQLN1 were transfected with CFP-HttQ105 and the localization analyzed by immunostaining (Myc-UBQLN1, green) or fluorescence (CFP-HttQ105, red). Yellow dashed lines are cell margins. (D) HEK293 cells were transfected with CFP-HttQ25 or CFP-HttQ105, with the latter separated by FACS into cells containing or lacking aggregates (see Figure S5D). The cells were separated into soluble (S) and insoluble (P) fractions, and analyzed by immunoblotting. The Bag6 blot and Ponceau-stained membrane (to detect the nuclear histones) illustrate uniform fractionation and loading. (E) HEK293 cells co-expressing CFP-HttQ105 and GFP-ATP5G1ΔMTS-2A-RFP were analyzed by flow cytometry. Histograms of the GFP:RFP ratio of the ATP5G1 reporter are shown for aggregate-containing (red) and -lacking (blue) cells. The profile for cells expressing CFP-HttQ25 is shown in gray for comparison. (F) Deconvoluted projection image of cells co-transfected with BFP-HttQ105 and GFP-ATP5G1ΔMTS-2A-RFP. Cells containing and lacking HttQ105 aggregates are indicated by yellow and blue arrows, respectively, in the merge image. See also Figure S5.

To design a constitutive UBQLN pathway client, we deleted the MTS from GFP-tagged ATP5G1 (GFP-ATP5G1ΔMTS) to force its translocation failure. An in-frame fusion with RFP separated by a viral 2A sequence (de Felipe et al., 2006) provided an internal expression control in which an RFP molecule is produced as a separate product every time a GFP-ATP5G1ΔMTS is translated (Figure 3A). Thus, the GFP:RFP ratio provides a quantitative indicator of the stability of the GFP-tagged protein relative to the long-lived RFP.

Relative to GFP-2A-RFP, most cells expressing GFP-ATP5G1ΔMTS-2A-RFP showed an ∼10- to 100-fold lower GFP:RFP ratio (Figure 3B). This ratio increased in TKO cells, and was partially reversed in rescue cells. The GFP-ATP5G1ΔMTS that was stabilized in TKO cells was primarily detergent insoluble (Figure S5A), and appeared to be in amorphous aggregates by microscopy (Figure S5B). The GFP:RFP ratio for the control construct did not change in TKO or rescue cells (Figure 3B), validating the specificity of the ATP5G1ΔMTS reporter.

As expected, CFP-tagged Huntingtin exon 1 containing 105 glutamines (HttQ105) formed aggregates in ∼25%–30% of transfected cells, whereas CFP-HttQ25 remained diffusely nucleocytoplasmic (Figures S5C and S5D). When CFP-HttQ105 was expressed in rescue cells, Myc-UBQLN1 was observed to co-associate with the HttQ105 aggregate, with corresponding depletion from the bulk cytosol in many (but not all) aggregate-containing cells (Figure 3C). Biochemical fractionation of these aggregate-containing cells verified that Myc-UBQLN1 was primarily in the insoluble pellet (along with HttQ105), with more than 50% depletion from the supernatant (Figure S5E). Myc-UBQLN1 was predominantly soluble in HttQ105 cells lacking aggregates, HttQ25-expressing cells, and non-transfected cells. Very similar results were observed for endogenous UBQLN2 in WT cells (Figure 3D). Thus, polyQ aggregates efficiently engage UBQLNs to markedly deplete their availability in the bulk cytosol.

Using the GFP:RFP ratiometric assay, we found that GFP-ATP5G1ΔMTS was preferentially stabilized in cells containing HttQ105 aggregates, but not HttQ25-expressing or aggregate-lacking HttQ105 cells (Figure 3E). The effect was a partial phenocopy of TKO cells (compare to Figure 3B), consistent with partial UBQLN depletion by HttQ105 aggregates. Importantly, the stabilized GFP-ATP5G1ΔMTS only partially co-localized with polyQ, as judged by fluorescence microscopy (Figure 3F) and flow cytometry (Figure S5F), arguing against co-aggregation as the sole explanation for its stabilization. Hence, UBQLN depletion by pathologic sequestration in polyQ aggregates contributes to impaired degradation of membrane protein precursors that fail successful mitochondrial insertion.

### UBQLN1 Recruits an E3 Ligase to Facilitate Client Ubiquitination

To understand the mechanistic basis of UBQLN-mediated degradation, we turned to biochemical studies. We observed that PURE system-produced Omp25-UBQLN1 complex added to cytosol resulted in Omp25 ubiquitination (Figure S6A). This is similar to Omp25 ubiquitination observed in complex with Bag6, a chaperone known to recruit an E3 ligase (Hessa et al., 2011, Rodrigo-Brenni et al., 2014). The specificity of ubiquitination was verified by the markedly lower levels seen for complexes with the targeting factor TRC40 or UBQLN1ΔUBA. These results suggested that UBQLN1 may facilitate client ubiquitination by recruiting an E3 ligase, possibly via its UBA domain.

**Figure 4:**
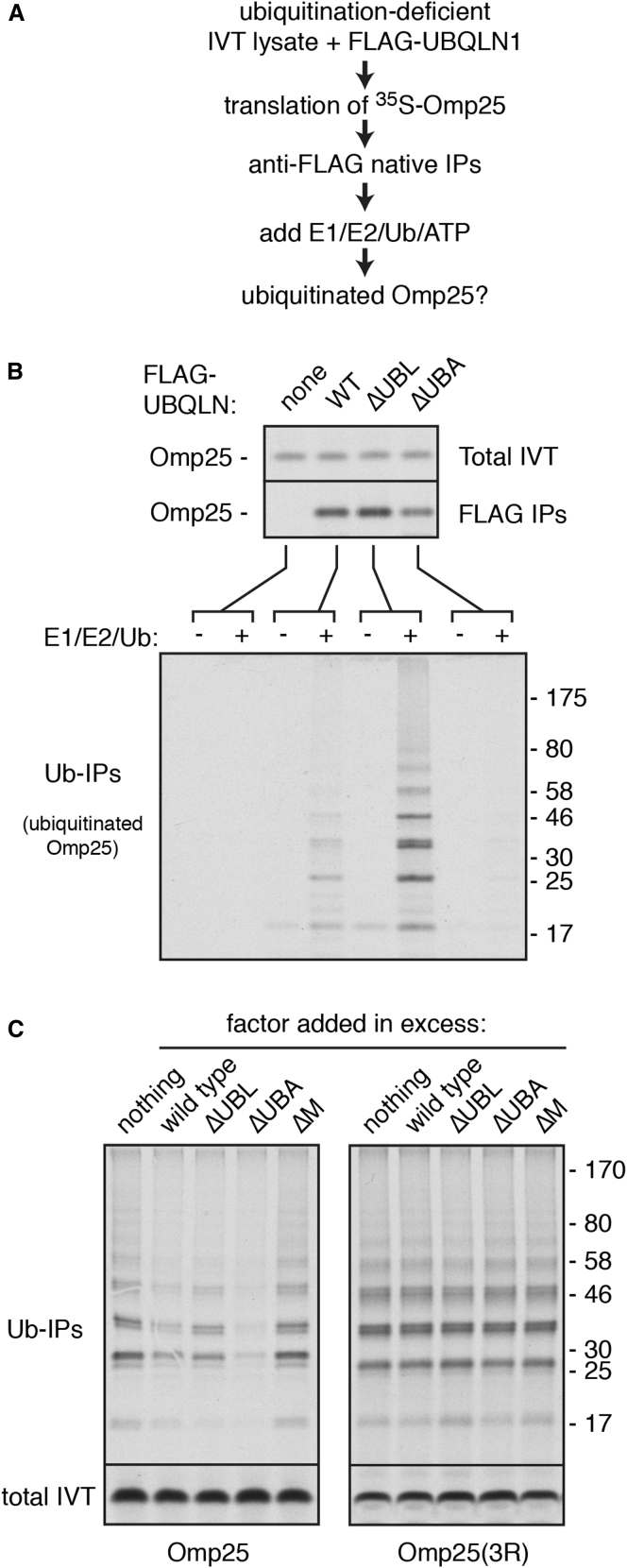
UBQLN1 Recruits an E3 Ligase for Client Ubiquitination (A) Experimental strategy to detect E3 ligase activity associated with UBQLN1-Omp25 complexes. (B) A construct containing the Omp25 TMD was translated in RRL depleted of ubiquitin and the UbcH5 E2 enzyme but supplemented with the indicated FLAG-tagged UBQLN1 protein and ^35^S-methionine. After translation, anti-FLAG affinity resin was used to isolate the UBQLN1-Omp25 complexes, the sample divided in two, and one half supplemented with a mixture of E1, E2, His-tagged Ubiquitin, and ATP. The reaction products were then affinity purified via the His tag on Ubiquitin, and the ^35^S-Omp25 was detected using autoradiography. Aliquots of the sample at each step are shown. (C) The UBQLN-associating client Omp25 and the non-associating Omp25(3R) mutant were translated in RRL containing ^35^S-methionine, the indicated UBQLN1 protein (at 1 μM), and His-tagged Ubiquitin (at 10 μM). Shown are aliquots of the total translation products and the ubiquitinated products isolated via His-tagged Ubiquitin. See also Figure S6.

To test this idea, we determined if an E3 ligase activity could be co-purified with Omp25-UBQLN1 complexes (Figure 4A). RRL was fractionated to remove free ubiquitin and UbcH5 (Hessa et al., 2011), and this ubiquitination-deficient lysate was used to synthesize Omp25 in the presence of FLAG-tagged UBQLN1. The Omp25-UBQLN1 complex was affinity purified via the FLAG tag and incubated with E1, E2, ATP, and His-tagged ubiquitin. Pulldowns via the His tag revealed that Omp25 was ubiquitinated in an E1/E2-dependent manner, indicating that the purified complexes contained an E3 ligase (Figure 4B).

Ubiquitination was not observed when UBQLN1ΔUBA was used, but was enhanced with UBQLN1ΔUBL. Deletion of the UBL domain presumably enhances ligase recruitment by freeing the UBA domain, with which the UBL domain can associate (Lowe et al., 2006, Zhang et al., 2008). Consistent with a key role for the UBA domain in ligase recruitment, we found that ubiquitination of Omp25 translated in RRL is dominantly inhibited most potently by excess UBQLN1ΔUBA (Figure 4C). In contrast, a TMD mutant (Omp25-3R) that cannot interact with UBQLNs (Figure 1B) is not inhibited in its ubiquitination by UBQLN1ΔUBA (Figure 4C). Similar results were observed for a different mitochondrial TA protein (Figure S6B). Thus, we conclude that UBQLN1 can recruit an E3 ligase in a UBA domain-dependent manner to mediate ubiquitination of its bound client.

### UBQLN1 Clients Are Insertion Competent Prior to Their Ubiquitination

**Figure 5:**
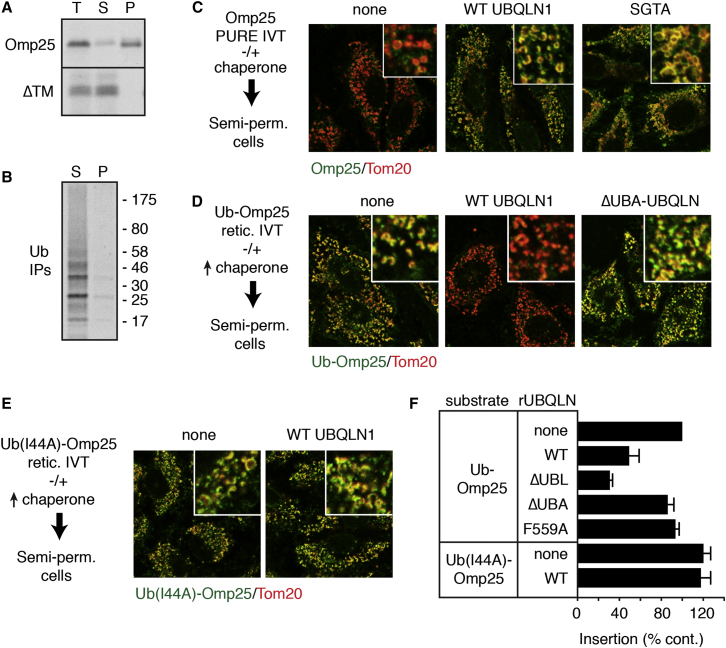
UBQLN1 Conditionally Inhibits Mitochondrial Insertion of Ubiquitinated Clients (A) Constructs containing the Omp25 TMD or a mutant disrupted in its hydrophobic domain (ΔTM) were translated in RRL in the presence of ^35^S-methionine and incubated with semi-permeabilized cultured cells. The total reactions (T) were fractionated into a soluble supernatant (S) and cell pellet (P), and the translated products were detected using autoradiography. (B) A construct containing the Omp25 TMD was translated in a reaction containing ^35^S-methionine, His-tagged Ubiquitin, and semi-permeabilized cells. After translation, the reaction was fractionated as in (A), the ubiquitinated products in each fraction isolated via the His tag, and the ubiquitinated Omp25 detected using autoradiography. (C) An HA-tagged construct containing the Omp25 TMD was translated in the PURE system supplemented without or with the indicated chaperone (either UBQLN1 or SGTA), and the products applied to semi-permeabilized cells. After washing, immunofluorescence microscopy was used to detect the localization of the translation product (green) relative to mitochondria (red). (D) An HA-ubiquitin-tagged construct containing the Omp25 TMD was translated in RRL containing 1 μM of the indicated UBQLN1 protein. The translation products were applied to semi-permeabilized cells and visualized as in (C). (E) As in (D), but with a construct containing the I44A mutation in Ubiquitin. (F) The indicated substrates were translated in RRL supplemented with the indicated recombinant UBQLN1 proteins and analyzed for insertion into the mitochondria of semi-permeabilized cells as in (A). The graph shows the normalized quantification from three experiments (mean ± SD). See also Figure S7.

During the course of our experiments, we observed that Omp25 targeting to mitochondria and ATP5G1 import were preferentially reduced for molecules that had acquired one or more ubiquitins (Figures 5A, 5B, and S7A). This result implies that there exists mechanism(s) to avoid targeting and import of ubiquitinated proteins. We therefore investigated the relationships between engagement of UBQLNs, membrane targeting, and ubiquitination.

To determine if engagement of UBQLN1 is a commitment to degradation, we tested the mitochondrial targeting competence of the Omp25-UBQLN1 complex assembled using the PURE system. Omp25 showed equally high and specific mitochondrial targeting when complexed with either UBQLN1 or SGTA, an unrelated TMD-binding chaperone (Figure 5C). The efficiency of insertion was comparable to that seen for Omp25 produced in RRL. This indicates that engagement of UBQLN1 is not mutually exclusive with insertion, and is not necessarily a commitment to degradation. As expected, Omp25 synthesized in the PURE system in the absence of any chaperone showed no capacity for specific membrane targeting. Thus, precluding aggregation of nascent Omp25 by either UBQLN1 or SGTA is sufficient to maintain insertion competence and permit mitochondrial targeting.

To test whether ubiquitination is the key step for switching from insertion competence to degradation, we generated a “constitutively” ubiquitinated Omp25 in which the deubiquitinase-resistant ubiquitin(G76V) was fused to the N terminus of Omp25 (Ub-Omp25). Translation of Ub-Omp25 in RRL supplemented with excess UBQLN1 (to outcompete capture by other chaperones) resulted in a complex that was sharply reduced in mitochondrial insertion (Figure 5D). This inhibitory effect was not observed with UBQLN1ΔUBA, suggesting that the interaction between ubiquitin on the client with the UBA domain of UBQLN1 may be important for terminating insertion competence.

This was demonstrated by showing that Ub-Omp25 regained insertion competence when ubiquitin contained the I44A mutation to inhibit its association with the UBA domain (Figures 5E and 5F). Similarly, the F559A mutation in the UBA domain, which disrupts ubiquitin binding (Zhang et al., 2008), also restored insertion competence to Ub-Omp25 (Figure 5F). Thus, the Omp25-UBQLN1 complex retains insertion competence until Omp25 acquires ubiquitin, whose inhibitory effect on insertion depends on interaction with the UBA domain of UBQLN1. Similar results were observed for ATP5G1 (Figure S7B).

### Mechanism of Fate-Switching by UBL-UBA-Ubiquitin Interactions

Having identified the key step when insertion competence is lost, we could investigate the mechanistic basis for this shift and determine how it might facilitate degradation. A pre-requisite for membrane insertion is release of the TMD from its protective chaperone. We therefore suspected that ubiquitination of a UBQLN1-bound client precludes insertion by preventing efficient release of the TMD. To test this idea, we modified the PURE system to re-assign the amber stop codon (UAG) to code for *p*-benzoyl-L-phenylalanine (BpF), a UV-activated photo-crosslinker (Chin et al., 2002). We used this system to produce UBQLN1-Omp25 complexes containing BpF in the TMD. Control experiments verified the production of UV- and BpF-dependent photo-adducts to UBQLN1, providing a means to monitor the Omp25-UBQLN1 interaction (data not shown). Importantly, the photo-crosslinking reaction can proceed on flash-frozen samples (e.g., Shao and Hegde, 2011), providing a snapshot of TMD interactions at any particular moment.

**Figure 6:**
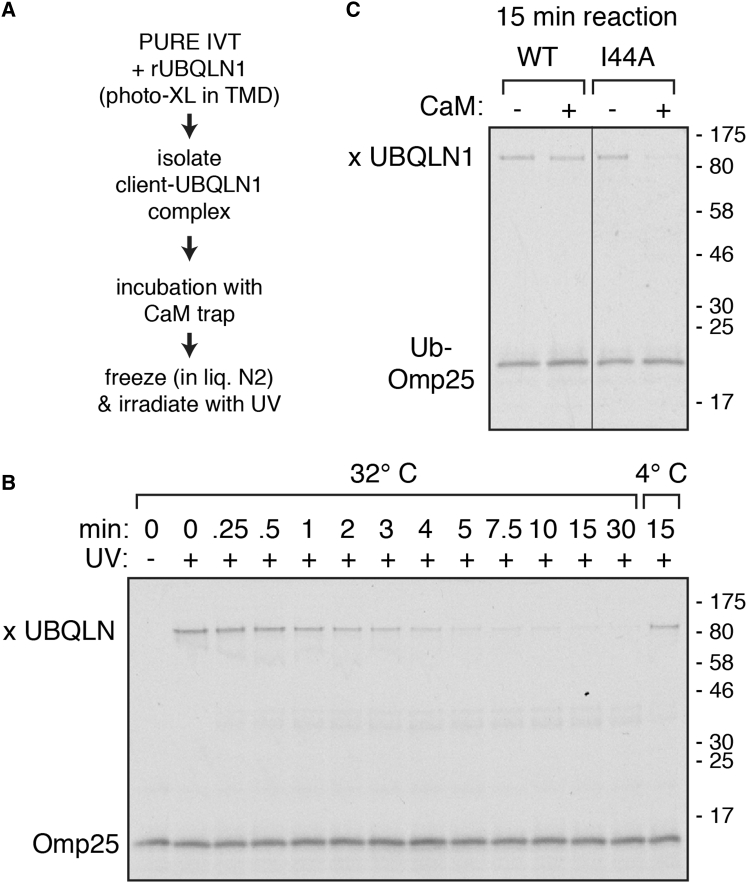
Client Ubiquitination Alters the Dynamics of UBQLN1 Interaction (A) Experimental strategy to detect Omp25 dissociation from UBQLN1. (B) A construct containing an amber codon in the Omp25 TMD was translated in a modified PURE system containing BpF-charged amber suppressor tRNA and recombinant UBQLN1. The UBQLN1-Omp25 complexes were isolated via sucrose gradient, mixed with 10-fold excess calmodulin (CaM), and incubated for the indicated times at either 32°C or 4°C. Aliquots were flash-frozen, irradiated with UV light in the frozen state, and analyzed by SDS-PAGE. Omp25 and its photo-adduct with UBQLN1 are indicated. Crosslinks to CaM are not clearly observed due to heterogeneity and lower efficiency of adduct formation, but can be seen faintly in the middle part of the gel. (C) Ub-Omp25 containing a photo-probe in the TMD, or a variant containing the I44A mutation in Ubiquitin, was assembled into a UBQLN1 complex as in (B). The sample was mixed with CaM, incubated for 15 min at 32°C, subjected to UV irradiation, and analyzed by SDS-PAGE and autoradiography. Ub-Omp25 and its photo-adduct with UBQLN1 are indicated.

Using this assay, we could determine whether client release from UBQLN1 is impeded by a ubiquitin on the client (Figure 6A). To prevent re-binding of client released from UBQLN1, we used calmodulin (CaM), a protein capable of efficient and stable binding to hydrophobic domains in the presence of high Ca^2+^ (Shao and Hegde, 2011), as a “sink” to capture exposed TMDs. Kinetics of Omp25 release showed a t_1/2_ of ∼1–2 min (Figure 6B), which is suitably fast to permit multiple opportunities for mitochondrial insertion before client ubiquitination. In contrast to Omp25, very little Ub-Omp25 was released from UBQLN1 in 15 min, whereas ∼80% of Ub(I44A)-Omp25 was released (Figure 6C). Thus, the UBQLN1-Omp25 complex dynamically dissociates and re-associates rapidly, whereas Ub-Omp25 is stably bound to UBQLN1 over physiologically relevant time frames.

**Figure 7:**
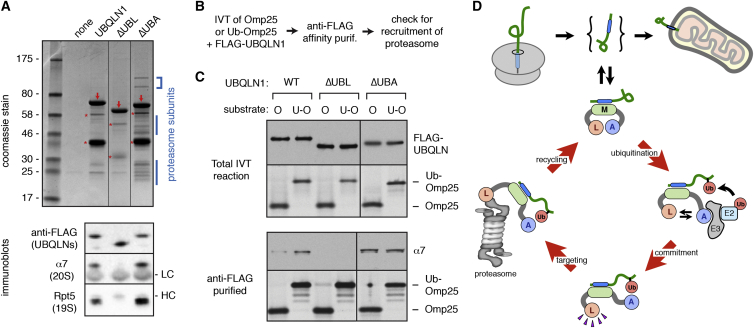
Client-Regulated Proteasome Targeting by UBQLN1 (A) Recombinant FLAG-tagged UBQLN1 or the indicated mutant was added to total RRL, incubated, recovered via anti-FLAG affinity resin, and analyzed by SDS-PAGE. The positions of the recombinant proteins (downward red arrows) and proteolytic fragments (red asterisks) are shown. Blue marks indicate proteasome subunits. Bottom: a similar analytic scale experiment analyzed by immunoblotting for the UBQLN1 protein and proteasome subunits. (B) Experimental strategy for analyzing proteasome targeting of UBQLN1-Omp25 complexes. (C) Constructs containing the Omp25 TMD without or with an in-line fused ubiquitin were translated in lysates lacking ubiquitin, UbcH5, and the major TMD-binding factors, but supplemented with the indicated recombinant UBQLN1 at 40 nM. After translation, UBQLN1-Omp25 complexes were affinity purified via the FLAG tag on UBQLN1, and the products analyzed by autoradiography (to detect the Omp25 substrate) or immunoblotting for the proteasome subunit α7. An aliquot of the starting translation reaction was analyzed in parallel (top). (D) Model for UBQLN function. TMD-containing proteins destined for the mitochondria can be intercepted by UBQLNs during their cytosolic transit. This binding is dynamic, permitting opportunities for mitochondrial targeting in the unbound state. Over time, the UBA domain (blue) facilitates recruitment of an E3 ligase to mediate client ubiquitination. This results in a conformational change that simultaneously increases client-UBQLN affinity (via UBA-ubiquitin interactions), while exposing the UBL domain (light orange). This re-structured complex is now favored for proteasome targeting.

In addition to precluding insertion, degradation should ideally be favored for an ubiquitinated UBQLN1 client. An important clue for how this occurs came from the comparative analysis of interaction partners for UBQLN1ΔUBA versus UBQLN1ΔUBL (Figure 7A): the former preferentially associated with the proteasome, while the latter did not. This suggested a model where engagement of ubiquitin on the client by UBQLN’s UBA domain frees the UBL domain to engage the proteasome.

To test this idea (Figure 7B), we first modified RRL by depleting all of its TMD-binding proteins (by passing over phenyl-sepharose) and removing free ubiquitin (by fractionation using anion-exchange). This translation system was then replenished with recombinant FLAG-tagged UBQLN1 variants at endogenous levels. This allows UBQLN1 to be the only TMD-binding protein for Omp25, whose ubiquitination status could be controlled by using Ub-Omp25. Following translation of a client in this system at levels sufficient to saturate the recombinant UBQLN1, the FLAG tag was used to capture UBQLN1 and its targeting to the proteasome was judged by immunoblotting.

Whereas UBQLN1ΔUBA constitutively associated with the proteasome, and UBQLN1ΔUBL failed to associate, WT UBQLN1 associated in a client-modulated manner (Figure 7C). UBQLN1 interaction with the proteasome was ∼2- to 3-fold higher when UBQLN1 was loaded with Ub-Omp25 relative to Omp25. This difference is similar to that observed between UBQLN1ΔUBA and WT UBQLN1, indicating that UBA domain sequestration by client ubiquitin was involved. Thus, UBQLN1 favors proteasome association when its client is ubiquitinated, and this depends on its UBL domain. The shift toward proteasome targeting accompanies a simultaneous inhibition of insertion capacity (Figure 5), with both events relying critically on the UBA interaction with client ubiquitin. Ubiquitin is therefore used by UBQLN1 to regulate fate-switching of the bound client.

## Discussion

In this study, we have discovered that UBQLNs are a new class of chaperones dedicated to shielding TMDs from aggregation during their transient residence in the cytosol. UBQLN specificity, while overlapping with other TMD chaperones, is tuned to favor mitochondrial membrane proteins due to their slightly lower overall hydrophobicity. Mechanistic analysis of UBQLN1 function indicates that beyond preventing client aggregation, it acts at the crossroads between mitochondrial membrane protein biogenesis and degradation. Hence, a key function of UBQLNs is to facilitate degradation of mitochondrial membrane proteins that fail successful insertion, a pathway likely to be of crucial importance to normal cellular physiology and various pathologic conditions.

The molecular basis of triage between biosynthesis and degradation by UBQLNs appears to be a multi-step process involving a sequence of dynamic and non-dynamic inter- and intramolecular interactions (Figure 7D). Engagement of UBQLN by a membrane protein is highly dynamic, and does not commit the client for degradation. This suggests that UBQLNs, whose total concentration in cytosol we estimate to be ∼1–2 μM, act as surveillance factors for exposed TMDs. Dynamic engagement by a relatively abundant factor not only protects against aggregation in the bound state, but also allows opportunities for engaging the yet unidentified targeting pathway to mitochondria in the unbound state.

This opportunity for mitochondrial targeting is time-limited by the contingency that, while associated with UBQLN, an E3 ligase can be recruited to ubiquitinate the client. The timing of ubiquitination is presumably determined by a combination of how long the ligase is bound to UBQLN and the kinetics of ligase activity. Once ubiquitinated, the previously dynamic client-UBQLN interaction changes to a comparatively stable interaction that precludes additional insertion attempts. Key to this stabilization is the UBA-ubiquitin interaction, whose K_d_ of ∼8 μM is 10-fold lower than the ∼80 μM K_d_ for the UBA-UBL interaction (Lowe et al., 2006). Thus, the UBA domain exploits the high local concentration of the client’s ubiquitin and the avidity gained by the M domain-TMD interaction to effectively switch the fate of membrane protein clients away from insertion. Ubiquitin binding to the UBA domain might also protect the client from deubiquitinases to limit the reversibility of this switch in fate.

The higher affinity interaction of the UBA domain with client ubiquitin relative to the UBL domain means the latter becomes preferentially freed only after the client is ubiquitinated. Hence, proteasome targeting via the UBL domain is favored for UBQLN containing a ubiquitinated client, explaining how futile excursions to the proteasome are minimized for UBQLN that is either empty or loaded with non-ubiquitinated client. Our tools to prepare defined UBQLN-client complexes capable of efficient proteasome targeting now open up opportunities to mechanistically dissect both the targeting reaction and the transfer of clients from UBQLN to the proteasome.

Based primarily on similarity to Rad23 domain organization (Su and Lau, 2009), earlier work had suggested that yeast Dsk2 (and by inference, the homologous UBQLNs) act as putative “shuttling factors” that deliver polyubiquitinated clients to the proteasome (Ghaboosi and Deshaies, 2007). Curiously, UBQLNs contain only one UBA domain relative to Rad23’s tandem UBAs, and hence UBQLNs may not have linkage-specificity for polyubiquitin chains (Zhang et al., 2008). In addition, the methionine-richness of the M domain is conserved from yeast to humans, but is not seen in Rad23. Our findings provide an explanation for these differences and revise the biological role for UBQLNs. Rather than simply bridging ubiquitin with the proteasome, UBQLNs encode client specificity in their M domains and actively participate in deciding the client’s fate via a combination of E3 ligase interactions and dynamic client interactions. Thus, the single low-affinity UBA domain is not selecting clients, but rather stabilizing them after their selection via the M domain and ubiquitination by UBQLN-associating E3 ligases.

In this light, the generic shuttling function assumed for other UBL-UBA proteins merits re-evaluation. Given the ability of intrinsic ubiquitin receptors on the proteasome to select ubiquitinated clients (Finley, 2009), it is perhaps more attractive to view the UBL-UBA proteins as a family of client-specific factors for regulated degradation or quality control. In this view, the UBA-UBL proteins may mediate client ubiquitination analogous to our findings with UBQLNs, or preferentially associate with only certain ubiquitinated clients as dictated by non-UBA substrate-binding region(s). More specific roles for these proteins may better explain their distinct phenotypes and evolutionary conservation of regions, such as the M domain of UBQLNs, outside the UBL-UBA regions (Su and Lau, 2009).

The function of UBQLNs in degrading non-inserted mitochondrial membrane proteins is analogous to the role of Bag6 complex in degrading mislocalized proteins destined for the ER (Hessa et al., 2011). Indeed, UBQLNs and Bag6 have overlapping client specificities, and both are capable of also impacting the respective biosynthesis pathways. Consistent with this conclusion, UBQLN4 was recently observed to influence degradation of proteins that fail to successfully translocate into the ER (Suzuki and Kawahara, 2016), a set of clients also handled by the Bag6 pathway (Hessa et al., 2011). It seems likely that UBQLNs and Bag6 act in partially redundant pathways for the management of diverse hydrophobic domains that must necessarily transit the cytosol en route to either an intracellular membrane or the proteasome. We have observed that while many substrates may prefer one or the other factor in the cytosol, they are capable of binding the non-preferred factor when the primary factor is absent. Such a system would impart substantial robustness to these quality control systems by having alternate pathways for degradation. This would come at the expense of taxing these alternative pathways when one pathway is ablated, causing a general “proteostasis” insufficiency (and an accompanying stress response as seen in TKO cells) that indirectly impacts the fate of substrates for other pathways.

Our assignment of a molecular function for UBQLNs in mitochondrial precursor degradation has implications for both cellular physiology and disease. Mitochondrial import efficiency is not uniformly high and is modulated by physiologic and pathologic stimuli (Harbauer et al., 2014, Schmidt et al., 2011, Wright et al., 2001). It is therefore intriguing that conditional knockout mice lacking UBQLN1 in mouse brain display increased sensitivity to oxidative stress (Liu et al., 2014), during which import is probably impaired (Wright et al., 2001). Furthermore, UBQLNs are upregulated in response to oxidative stress (Ko et al., 2002), supporting a potential role in aiding recovery from the stress.

Conversely, conditions that deplete or impair UBQLNs would affect cytosolic proteostasis. Hence, the finding that UBQLNs are substantially depleted from the bulk cytosol of cells containing polyQ aggregates may help explain their impairment in degradation of a UBQLN client. As these clients would spill over into other quality control pathways, their increased demand could result in the more generalized effects on proteostasis observed in many studies (Labbadia and Morimoto, 2015). It is noteworthy that while a wide range of proteins have been found in aggregates (Olzscha et al., 2011), the precise level of depletion has rarely been defined. In the case of UBQLNs at endogenous levels, the extent of sequestration is clearly sufficient to deplete it from the cytosol. Thus, part of the phenotype caused by polyQ aggregates may result from UBQLN insufficiency, perhaps explaining why UBQLN1 overexpression can partially rescue a mouse model of polyQ disease (Safren et al., 2014). With our introduction of a range of cellular and biochemical assays for UBQLN function, it should now be possible to examine these ideas with greater precision, including the prospect of determining why certain mutations in UBQLN2 cause neurodegenerative disease in humans (Deng et al., 2011).

## Experimental Procedures

### Plasmids, Antibodies, and Proteins

A list of plasmids is provided in Table S1. Plasmids were prepared by standard methods. Antibodies against the following proteins were purchased: UBQLN1/2 (Sigma, clone 5F5), UBQLN4 (Abcam ab106443), L9 and Tom20 (Santa Cruz Biotech., T-17 and FL-145), HA (Covance, clone 16B12), Rpt5 (Abcam ab22635), α7 (Enzo Life Sciences PW8110), FLAG (Sigma, clone M2), Hsc/Hsp70 (AssayDesigns, SPA-822), Hsp60 (Abcam ab46798), ClpP (Abcam ab124822), Actin-HRP (Sigma A3854), OxPhos cocktail (Abcam ab110411). FLAG-M2 Affinity resin was from Sigma, and GFP-trap from Chromotek. Antibodies to Bag6, TRC40, SGTA, TRAPα, GFP, and RFP have been described (Fons et al., 2003, Mariappan et al., 2010, Hessa et al., 2011). Anti-Myc was clone 9E10. Anti-HA used for IPs and blots (e.g., Figure 2A) was raised in rabbits against the KLH-HA peptide conjugate. Recombinant proteins were either purchased (His6-Ubiquitin, E1, and E2 from Boston Biochem) or expressed and purified from *E*. *coli* as described (Mateja et al., 2015).

### Cell Culture

HeLa and Flp-in T-Rex HEK293 cells (Invitrogen) were cultured in DMEM supplemented with 10% fetal bovine serum and appropriate selection antibiotics. Gene disruption in HEK293 cells by CRISPR was performed as described (Ran et al., 2013) with guide RNA sequences provided in the Supplemental Experimental Procedures. Rescue cells were made by integrating a Myc-UBQLN1 expression cassette into the FRT site of TKO cells by Flp recombination (Invitrogen). 10 ng/ml doxycycline was used for induction of the integrated gene at the FRT site. Flow cytometry and FACS separation based on aggregates, pulse-chase experiments, fluorescence microscopy, and the detergent solubility assay have been described (Ramdzan et al., 2012, Ashok and Hegde, 2009).

### In Vitro Biochemistry

In vitro translation (IVT) reactions in the RRL and PURE systems were as described (Sharma et al., 2010, Shimizu and Ueda, 2010). DEAE and/or phenyl-sepharose fractionation of RRL was performed as described elsewhere (Hessa et al., 2011, Shao and Hegde, 2011). Site-specific photo-crosslinker incorporation by amber suppression in the PURE system was performed as described elsewhere (Shimizu et al., 2001). Affinity purification of IVT translation products and mass spectrometry (Stefanovic and Hegde, 2007), ubiquitination analysis (Rodrigo-Brenni et al., 2014), photo-crosslinking (Shao and Hegde, 2011), targeting assays in semi-permeabilized cells (Setoguchi et al., 2006), sucrose gradient separations (Stefanovic and Hegde, 2007), and preparation of chaperone-client complexes in the PURE system (Mateja et al., 2015) have all been described.

## Author Contributions

Conceptualization, R.S.H. and E.I.; Investigation, E.I., E.Z., S.S., M.L.W., and R.S.H.; Writing—Original Draft, R.S.H.; Writing—Review and Editing, E.I., E.Z., S.S., M.L.V., R.J.K., R.S.H.; Supervision, R.S.H. and R.J.K.

## Supplemental Information

Document S1. Supplemental Experimental Procedures, Figures S1–S7, and Table S1

Document S2. Article plus Supplemental Information
