# Supplementary material for: Ubiquilins Chaperone and Triage Mitochondrial Membrane Proteins for Degradation
Source: Mol Cell. 2016 Jul 7;63(1):21–33. doi: 10.1016/j.molcel.2016.05.020 (PMC4942676; doi:10.1016/j.molcel.2016.05.020)
Supplement: Document S1. Supplemental Experimental Procedures, Figures S1–S7, and Table S1 [file mmc1.pdf]

Molecular Cell, Volume *63*

Supplemental Information

Ubiquilins Chaperone and Triage Mitochondrial Membrane Proteins for Degradation

Eisuke Itakura, Eszter Zavodszky, Sichen Shao, Matthew L. Wohlever, Robert J. Keenan, and Ramanujan S. Hegde

Itakura, Fig. S1 (related to Fig. 1)

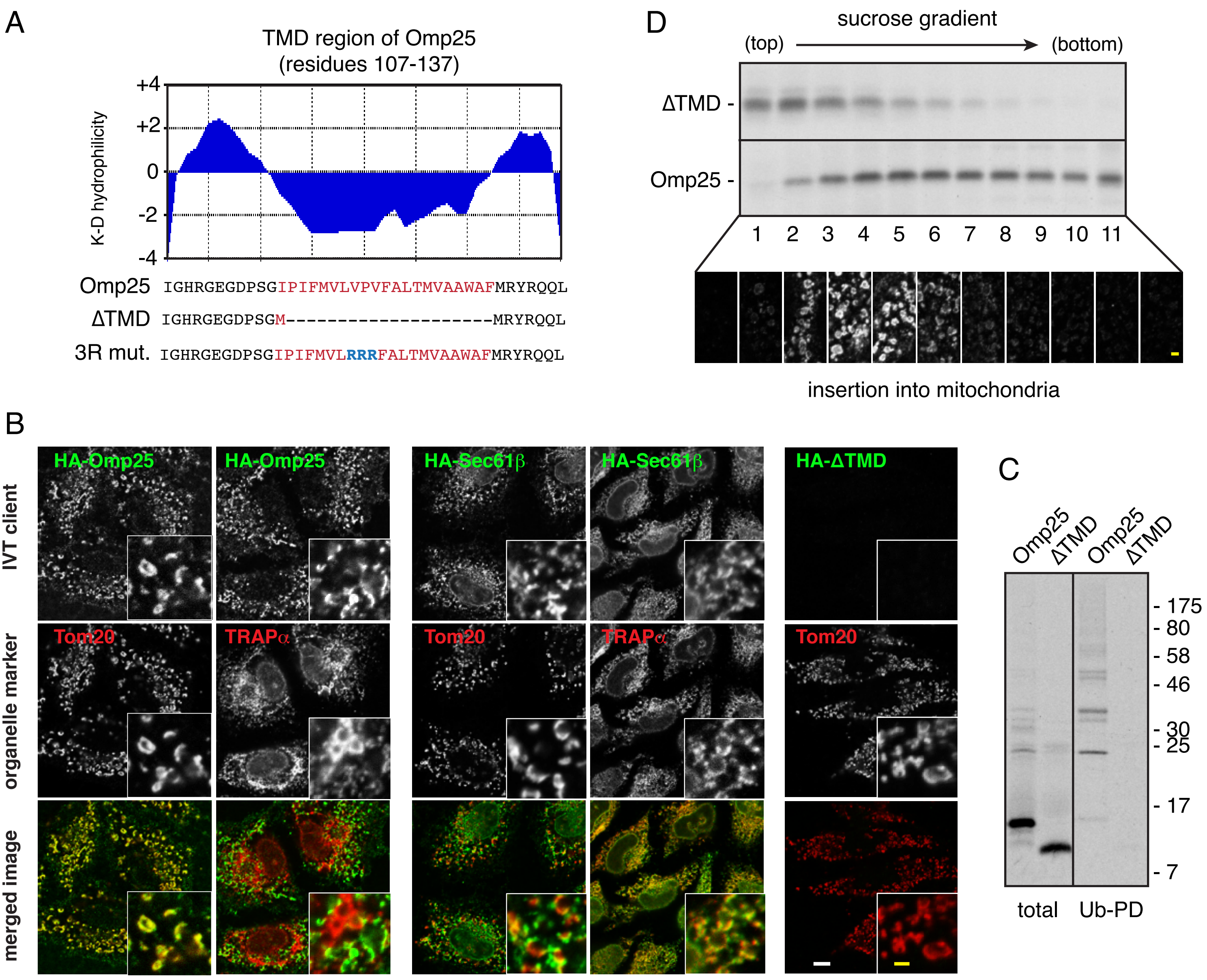

Fig. S1 (related to Fig. 1). Characterization of Omp25 targeting and ubiquitination. (**A**) Sequence and hydrophilicity plot of the TMD region used in all of the Omp25 constructs analyzed in this study. Sequences of the ΔTMD and 3R mutants are also shown. In a series of preliminary experiments, we confirmed that this TMD region at the C-terminus is necessary and sufficient for Omp25 targeting to mitochondria, and for its ubiquitination. Thus, the N-terminal soluble domain of Omp25 could therefore be replaced with any other soluble domains without consequences for targeting or ubiquitination. We therefore produced a number of different N-terminally tagged versions of the Omp25 TMD region (see Table S1) and used them essentially interchangeably as dictated by the requirements for any particular experiment. (**B**) HA-tagged constructs encoding the TMD region of Omp25 or full length Sec61β were translated in reticulocyte lysate and applied to semi-permeabilized HeLa cells. After the incubation, the cells were washed, fixed, and immunostained for the HA tag (green) or an organelle marker (red; Tom20 for mitochondria, or TRAPα for ER). Insets show an enlarged region of one of the cells. A matched ΔTMD control for Omp25 is also shown for comparison. All images were captured using identical settings. Note that mitochondrial targeting of Omp25 is dependent on the hydrophobic domain of the TMD, and that Omp25 and Sec61β are targeted selectively to mitochondria or ER, respectively. (**C**) Matched constructs containing the TMD region of Omp25 or the ΔTMD mutant were translated in reticulocyte lysate containing ^{35}S-methionine and His-tagged Ubiquitin. The samples were either analyzed directly (total) or after isolation of the ubiquitinated products via the His tag (Ub-PD). The products were detected by autoradiography. Note that Omp25 ubiquitination is dependent on the hydrophobic domain of the TMD. (**D**) Constructs containing the Omp25 TMD or the ΔTMD mutant were translated in reticulocyte lysate containing ^{35}S-methionine and separated on a 5-25% sucrose gradient into 11 fractions. Note that while the ΔTMD construct migrates near the top of the gradient consistent with its small size, Omp25 migrates heterogeneously deeper into the gradient, consistent with its engagement in higher molecular weight complexes. In a separate non-radioactive translation reaction of Omp25 fractionated by the same method, each fraction was applied to semi-permeabilized cells and monitored for mitochondrial targeting by immunostaining as in panel B. Note that Omp25-containing complexes in fractions 3-6 display high insertion activity, while complexes in fractions 7-11 are largely inactive. For reference, a native protein of ~60-80 kD would have its peak in fraction 4.

Itakura, Fig. S2 (related to Fig. 1)

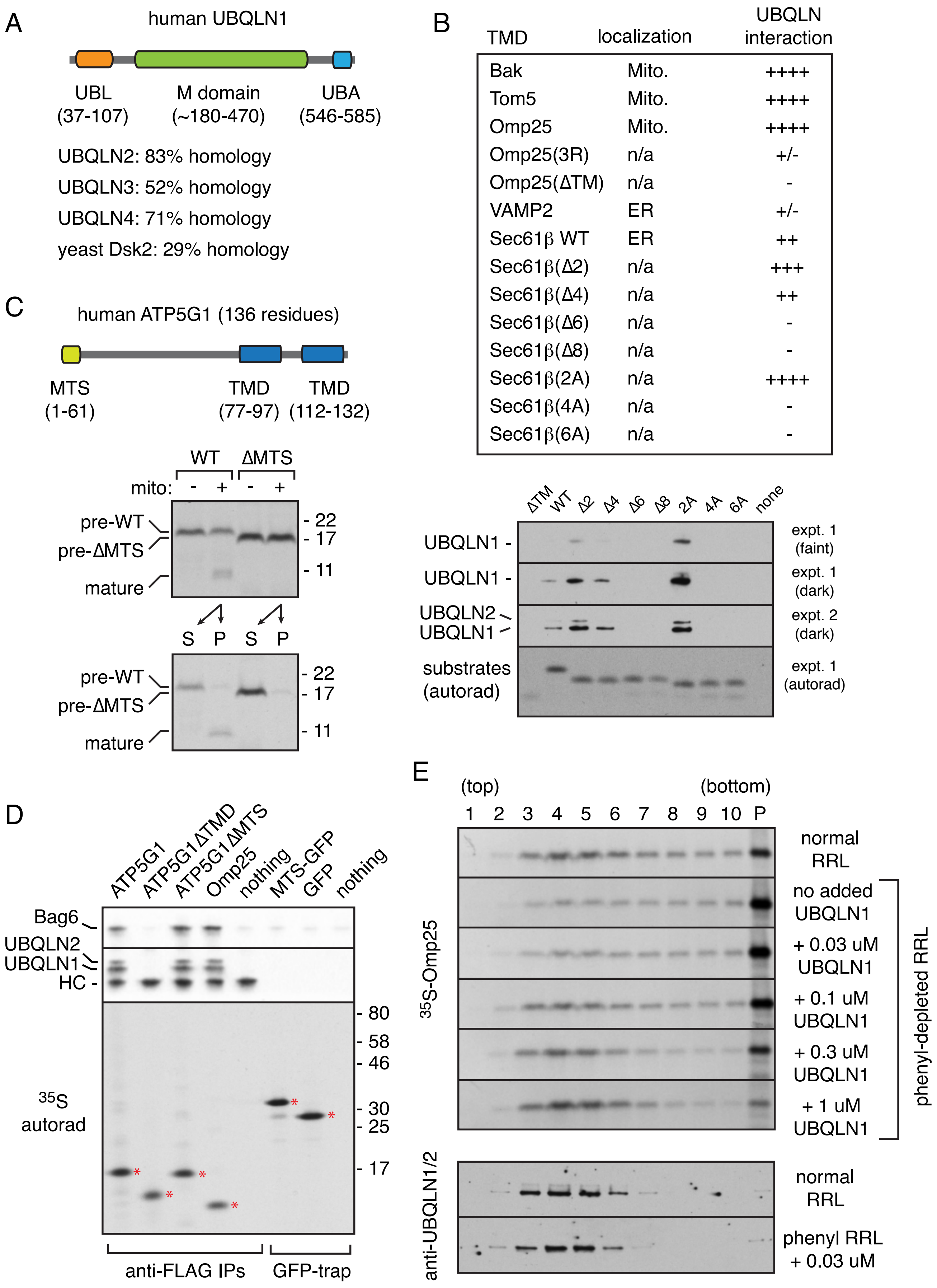

TMD	localization	UBQLN interaction
Bak	Mito.	++++
Tom5	Mito.	++++
Omp25	Mito.	++++
Omp25(3R)	n/a	+/-
Omp25(ΔTM)	n/a	-
VAMP2	ER	+/-
Sec61β WT	ER	++
Sec61β(Δ2)	n/a	+++
Sec61β(Δ4)	n/a	++
Sec61β(Δ6)	n/a	-
Sec61β(Δ8)	n/a	-
Sec61β(2A)	n/a	++++
Sec61β(4A)	n/a	-
Sec61β(6A)	n/a	-

Fig. S2 (related to Fig. 1). Characterization of UBQLN-client interactions. (**A**) Schematic of human Ubiquilin 1 architecture showing the relative positions of the key domains. UBL is the ubiquitin-like domain, UBA is the ubiquitin-associating domain, and the middle "M" domain is the highly methionine-rich region within which putative "Sti1" motifs of uncertain function have been suggested. Although Sti1 domains can potentially bind to heat shock proteins, we have yet to detect such an interaction with UBQLNs in our studies. Percent homology (the sum of identical and similar residues) with other human UBQLN family members and yeast Dsk2 are indicated. (**B**) Table summarizing the relative interactions of the indicated TMDs with UBQLNs as assayed in Fig. 1B and 1C. Bak, Tom5, and Omp25 are all mitochondrially targeted tail-anchored membrane proteins. VAMP2 and Sec61β are ER targeted tail-anchored proteins. The various mutants of Sec61β decrease the hydrophobicity of its TMD by either deleting residues or replacing them with Alanine. The gels below the table show the primary data from two independent experiments for this series of Sec61β TMD constructs. Two exposures of the immunoblot are shown for experiment 1 to better illustrate differences among the strongest interactors. The sum of these analyses indicate that UBQLN interaction depends critically on hydrophobicity of sufficient length to be a TMD, and is disfavored from binding the most hydrophobic TMDs (which are Sec61β and VAMP2). (**C**) Domain diagram of human ATP5G1, an inner mitochondrial membrane protein subunit of ATP synthase, showing its N-terminal mitochondrial targeting sequence (MTS) and two TMDs. The gels show that ATP5G1 is imported into mitochondria in vitro, where its MTS is removed. The wild type (WT) or ΔMTS mutant (lacking residues 1-25) were translated in reticulocyte lysate (RRL) containing ^{35}S-methionine, then incubated with isolated yeast mitochondria. Aliquots of the total products of these reactions are shown in the top gel. In parallel, the products of an insertion reaction were separated into a soluble supernatant (S) and a mitochondrial pellet (P), and shown in the bottom gel. Mature ATP5G1 co-sediments with mitochondria, while the ΔMTS mutant completely prevents import and processing. (**D**) The indicated constructs were translated in RRL containing ^{35}S-methionine, affinity purified, and analyzed by immunoblotting and autoradiography. Negative controls lacking substrate were analyzed in parallel. ATP5G1, its deletion mutants, and Omp25 were FLAG tagged, while the isolated MTS (from cytochrome c) was GFP-tagged. Note that ATP5G1 interacts with Ubiquilins as efficiently as Omp25, and this interaction is dependent on the TMDs. The MTS of neither ATP5G1 nor cytochrome c interacts with UBQLNs. HC indicates IgG heavy chain. (**E**) A construct containing the Omp25 TMD region was translated in RRL containing ^{35}S-methionine and separated on a 5-25% sucrose gradient into 10 fractions and the high molecular weight pellet (P). Translation was performed in either complete RRL or RRL that had been passed over phenyl sepharose to deplete most hydrophobic binding proteins (including UBQLNs, TRC40, Bag6, and SGTA). The phenyl-depleted RRL was supplemented with recombinant UBQLN1 to the indicated concentrations before use in the translation reaction. Note that the amount of Omp25 in the insoluble pellet is increased in phenyl-depleted RRL (with a corresponding decrease from fractions 3-6), and this behavior is progressively rescued by increasing concentrations of UBLQN1. The immunoblots below show the position of UBQLN1 migration in the gradient, illustrating that endogenous and recombinant UBLQN1 migrate similarly. Note that at the low concentration of 0.03 mM, all of the UBQLN1 is expected to be fully engaged by Omp25, indicating that the UBQLN1-Omp25 complex is likely to contain a single UBLQN1.

Itakura, Fig. S3 (related to Fig. 2)

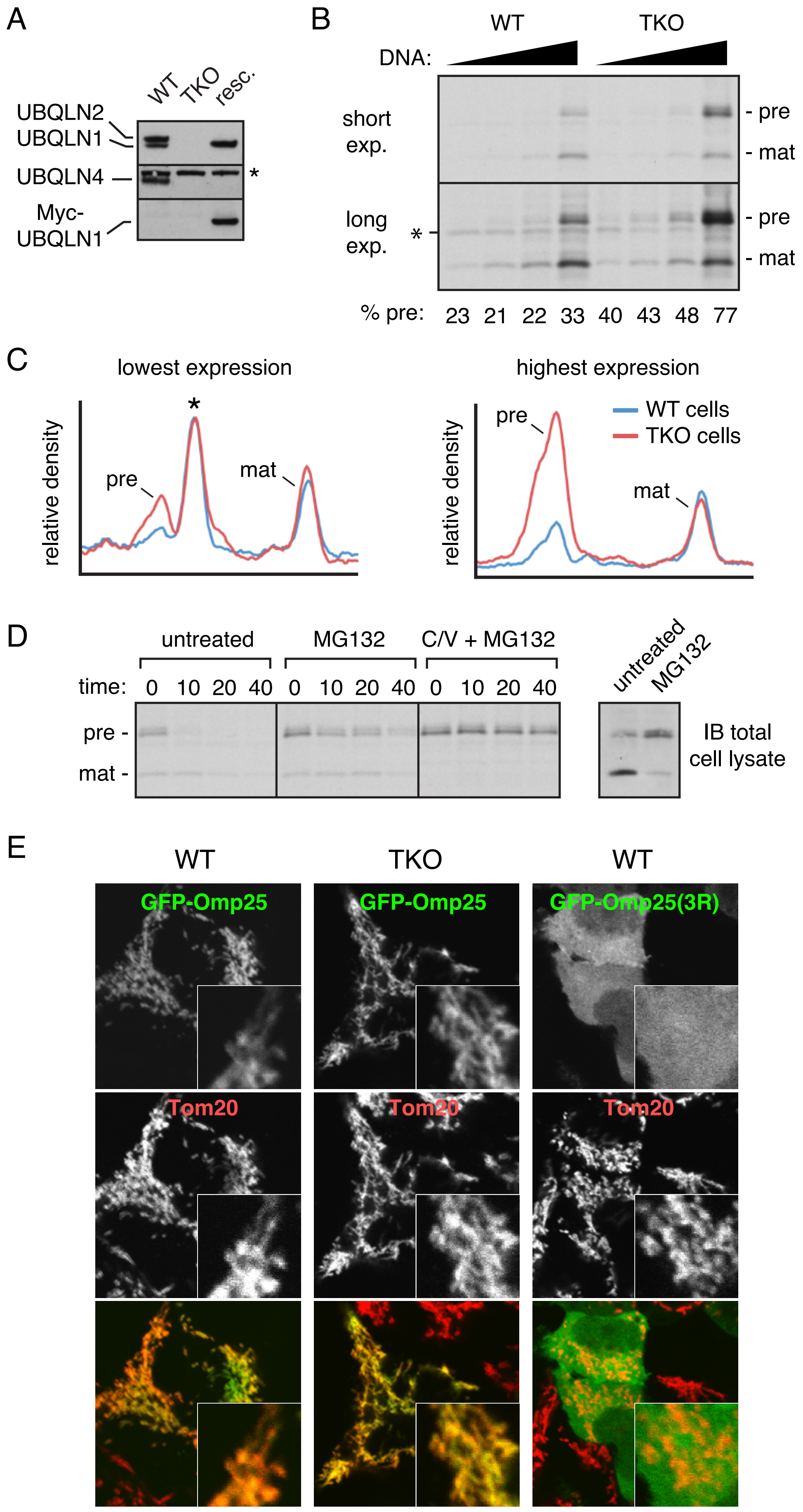

Fig. S3 (related to Fig. 2). Characterization of UBQLN triple knockout cells. (**A**) Immunoblotting of parental (WT), UBQLN1/2/4 triple knockout (TKO), and Myc-UBQLN1 rescued (resc.) HEK293 cells. Asterisk indicates a background band that serves as a loading control. (**B**) WT and TKO cells were transfected with a mixture of ATP5G1-HA and GFP expression plasmids in different ratios (1:5, 2:4, 3:3, and 6:0) to modulate the relative expression of ATP5G1. These cells were pulse labelled for 30 min with ^{35}S-methionine, the ATP5G1-HA was immunoprecipitated, and products were visualized by SDS-PAGE and autoradiography. The positions of precursor and mature forms of ATP5G1-HA are indicated. Short and long exposures of the gel are shown to visualize the highest and lowest expression samples, respectively. The asterisk indicates a background band (probably one of the histone proteins) recovered due to non-specific binding to the beads. Phosphorimaging was used to quantify the relative levels of precursor and mature forms in each sample and determine the percent of precursor (indicated below the gel). Note that the absolute amount of mature protein is very similar between WT and TKO cells across all expression levels. By contrast, the amount of precursor (and hence, the % precursor) is consistently higher in the TKO cells, consistent with its impaired degradation during the period of pulse labelling. Expression of ATP5G1-HA spanned a ~40-fold range (as determined by phosphorimaging) from the lowest to highest levels. This indicates that UBQLN-mediated precursor degradation is occurring at a variety of expression levels, and consistently accounts for ~15-20% of total synthesized ATP5G1-HA. This demand is elevated at the highest expression level, presumably due to some saturation of the import machinery. (**C**) Densitometry traces of the lowest (left) and highest (right) expression lanes from panel B illustrating the preferential increase in precursor in TKO cells (red traces). The positions of precursor (pre) and mature (mat) forms of ATP5G1-HA are indicated, as is the background band (asterisk). (**D**) WT cells expressing ATP5G1-HA were pulse-labeled with ^{35}S-methionine for 5 min and chased for up to 40 min with unlabeled methionine. Immunoprecipitated ATP5G1-HA was visualized by autoradiography. Where indicated, the proteasome was inhibited with MG132 and mitochondrial import was inhibited with a mixture of CCCP and Valinomycin (C/V). The positions of precursor (pre) and mature (mat) forms of ATP5G1 are indicated. Quantification of the MG132-treated pulse-chase samples by phosphorimaging indicates that ~25% of initially synthesized ATP5G1-HA is ordinarily degraded by a proteasome-dependent pathway. This pulse-chase experiment is part of the same experiment shown in Fig. 2B, and the 'C/V + MG132' panel for WT cells is duplicated here for comparison. The right panel shows an anti-HA immunoblot of untreated and MG132-treated (for 5 h) total cell lysate to detect steady state levels of ATP5G1-HA. The lower level of mature form in the MG132 treated lane of the immunoblot is due to some inhibition of protein synthesis during chronic proteasome inhibition. The important observation here is the preferential stabilization of the precursor, consistent with the pulse-chase results. (**E**) GFP-Omp25 and the 3R mutant were transiently transfected into WT and TKO cells as indicated, and their localization relative to mitochondria (stained with anti-Tom20) was determined by confocal microscopy. Note that in TKO cells, mitochondrial morphology is grossly normal, and that GFP-Omp25 is localized correctly and quantitatively. Comparison of the amount of GFP-Omp25 at mitochondria across numerous WT versus TKO cells showed no systematic differences, indicating that its successful targeting is similar in these two cell lines.

Itakura, Fig. S4 (related to Fig. 2)

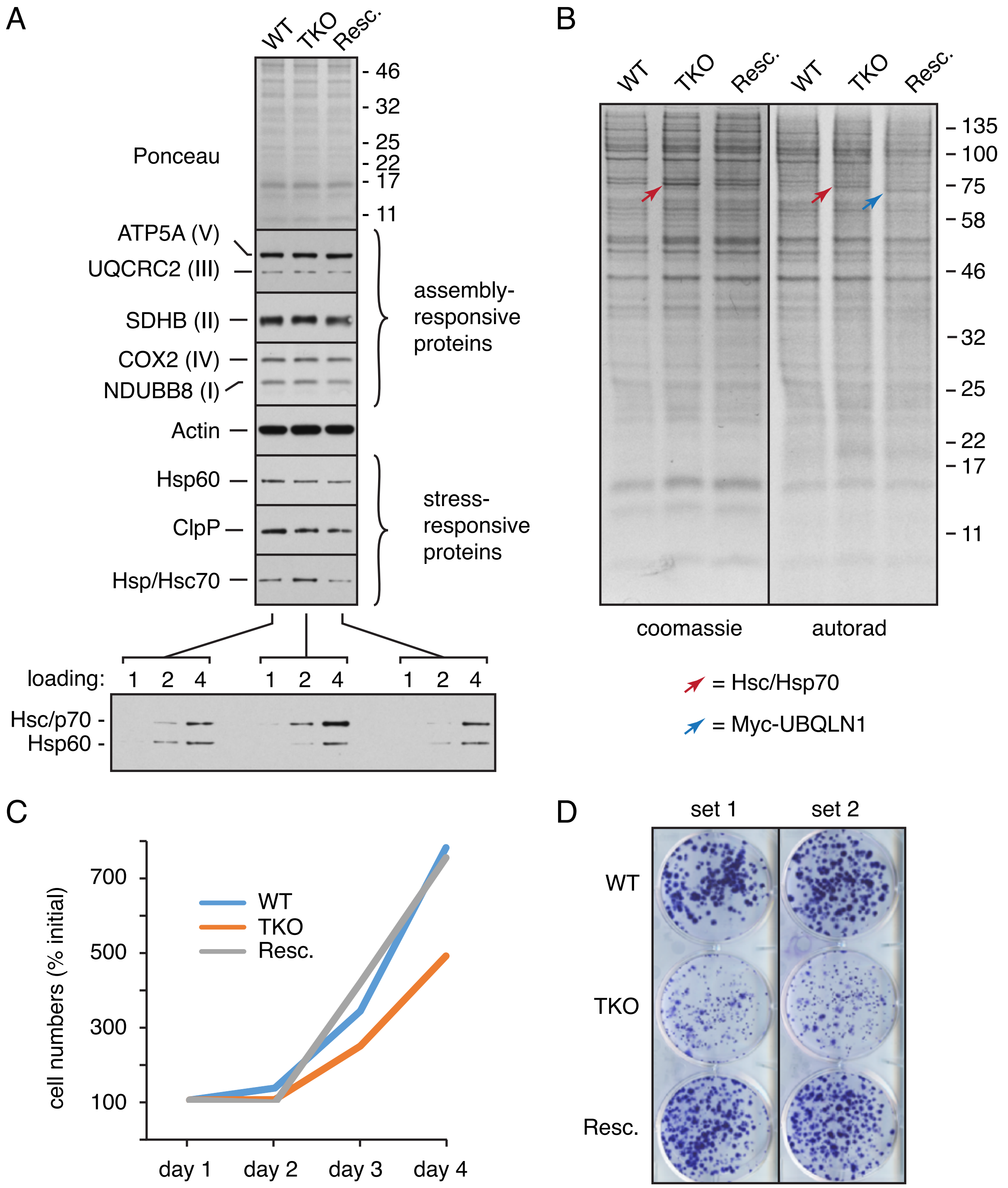

Fig. S4 (related to Fig. 2). Characterization of UBQLN triple knockout cells. (**A**) Equal amounts of total cell lysate prepared from WT, TKO, and rescue cells were analyzed by SDS-PAGE, blotted, and visualized for total protein with Ponceau stain (top panel) and the indicated antigens were detected by immunostaining. The five 'assembly-responsive proteins' are each core components of respiratory complexes I through V (all detected using the human OxPhos cocktail of monoclonal antibodies from Abcam) whose levels are sensitive to deficiencies in assembly of the respective complexes. Hsp60 and ClpP are mitochondrial proteins whose levels are increased by mitochondrial stress, while Hsp70 levels increase during cytosolic stress. The only protein systematically observed in multiple independent samples to be selectively changed in TKO cells was Hsc70/Hsp70 (the antibody detects both isoforms), as verified by analysis of serial dilutions of the three cell lysates (bottom panel) immunoblotted simultaneously for Hsc/Hsp70 and Hsp60. The numbers above the lanes in the bottom panel indicate the relative amounts loaded in each lane. (**B**) WT, TKO, and rescue cells were pulse labeled for 5 min with ^{35}S-methionine and analyzed by SDS-PAGE and coomassie staining (left panel) or autoradiography (right panel). The red arrow points to an abundant 70 kD protein (which we presume is Hsp70 based on the blots in panel A) whose levels are increased selectively in TKO cells at steady state (left panel) and whose rate of synthesis is notably higher (right panel). This indicates that the TKO cells are actively experiencing cytosolic stress. The blue arrow points to Myc-UBQLN1, whose labeling is unusually high due to its very high methionine content. UBQLNs are not easily discernable in the WT cells because the signal is distributed among UBQLN1/2/4. (**C**) Growth rate of WT, TKO, and rescue cells measured over four days after seeding. (**D**) Size of colonies of WT, TKO, and rescue cells formed over 14 days of growth after 100 cells were seeded into individual wells of a 6-well plate. Two replicates are shown.

Itakura, Fig. S5 (related to Fig. 3)

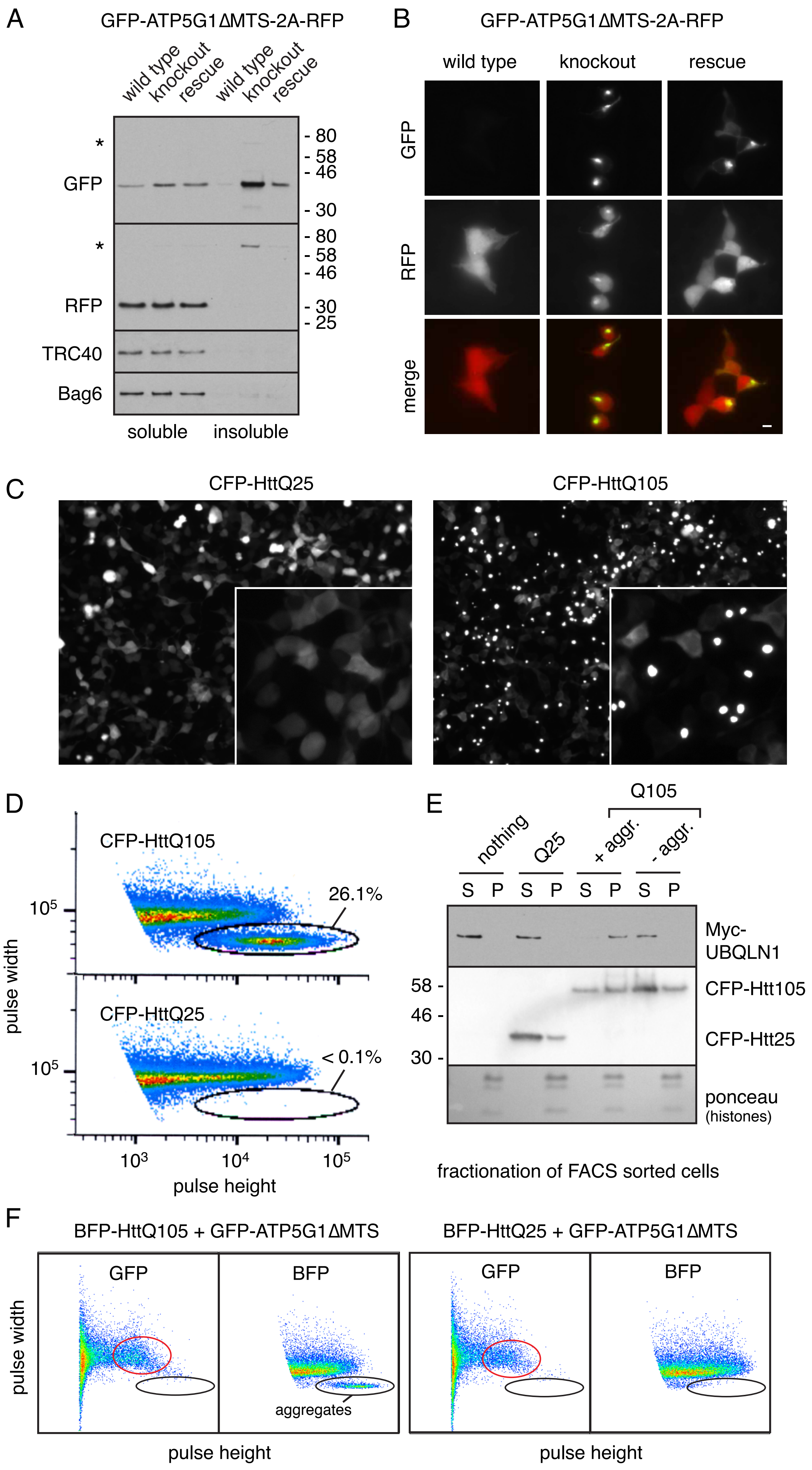

Fig. S5 (related to Fig. 3). Characterization of protein aggregation. (**A**) GFP-ATP5G1ΔMTS-2A-RFP was transfected into the indicated HEK293 cell lines, the cells harvested in non-denaturing detergent, and the lysates separated into soluble and insoluble fractions. The samples were blotted for GFP, RFP, TRC40, and Bag6. Note that the vast majority of translated RFP is appropriately separated at the 2A sequence, with only a minor amount of detectable fusion protein (asterisks in the GFP and RFP blots). As expected, the RFP is uniformly soluble in wild type, UBQLN triple knockout (TKO), and UBQLN1 rescue cells. By contrast, the GFP-ATP5G1ΔMTS fusion protein behaves differently in the three cell lines. Not only is the overall level higher in TKO cells, but a substantial proportion is found in the insoluble fraction, presumably representing aggregated protein. The increased levels and aggregation are partially reversed in knockout cells re-expressing UBQLN1. Note that the small amount of uncleaved GFP-ATP5G1ΔMTS-2A-RFP protein (asterisk) also accumulates preferentially in the insoluble fraction in TKO cells. TRC40 and Bag6 remain uniformly soluble in all cell lines and serve as fractionation and loading controls. (**B**) Cells transfected as in panel A were visualized by fluorescence microscopy to detect the localization of GFP-ATP5G1ΔMTS and RFP. Consistent with the biochemical analysis in panel A, RFP is uniformly distributed in the nucleocytoplasmic compartment of all cells. By contrast, the GFP signal is increased in knockout cells, where it is seen in puncta consistent with its aggregation. This is partially reversed in some, but not all cells upon UBQLN1 rescue. Scale bar is 10 µm. (**C**) View of a field of cells (with one region enlarged in the inset) of cells transfected with CFP-HttQ25 or CFP-HttQ105, illustrating that the latter is preferentially seen in puncta in many, but not all cells. (**D**) Aggregate-containing cells expressing CFP-HttQ105 can be separated from aggregate-lacking cells by flow cytometry using their differences in pulse height versus width. As expected from panel C, CFP-HttQ25 does not form aggregates. Quantification indicates ~25-30% of HttQ105-expressing cells contain aggregates in any typical experiment. (**E**) HEK293 rescue cells (knocked out for UBQLN1/2/4 but rescued with UBQLN1 re-expression) were transfected with CFP-HttQ25 or CFP-HttQ105. The CFP-HttQ105 cells were further separated by FACS into cells containing or lacking aggregates (as in panel D). Each set of cells was lysed in non-denaturing detergent, separated by centrifugation into supernatant (S) and pellet (P) fractions, and analyzed by immunoblotting and Ponceau-staining (to visualize the nuclear histones). Note that the majority of UBQLN1 is soluble, and this property does not change with expression of CFP-HttQ25. By contrast, cells containing CFP-HttQ105 aggregates result in most of the UBQLN1 now residing in the pellet fraction. This re-distribution was not seen in cells lacking aggregates, but nonetheless expressing CFP-HttQ105 at comparable levels. (**F**) HEK293 cells co-transfected with GFP-ATP5G1ΔMTS-2A-RFP and either BFP-HttQ105 or BFP-HttQ25 were analyzed by flow cytometry in both color channels (BFP and GFP) for pulse height versus width as in panel D. Aggregates of BFP-HttQ105, seen as a narrower pulse width relative to height, are not observed for HttQ25. Importantly, co-expressed GFP-ATP5G1 in these same cells does not show the narrow pulse width property in an HttQ105-dependent manner, indicating that the GFP signal is not exclusively co-localized (and hence, not quantitatively co-aggregated with) the BFP signal. The red circle indicates the area where an increased proportion of cells are seen in the HttQ105 cells relative to the HttQ25 cells. These represent cells whose GFP-ATP5G1 levels are increased due to stabilization, and represent specifically the cells containing HttQ105 aggregates (see Fig. 3E).

Itakura, Fig. S6 (related to Fig. 4)

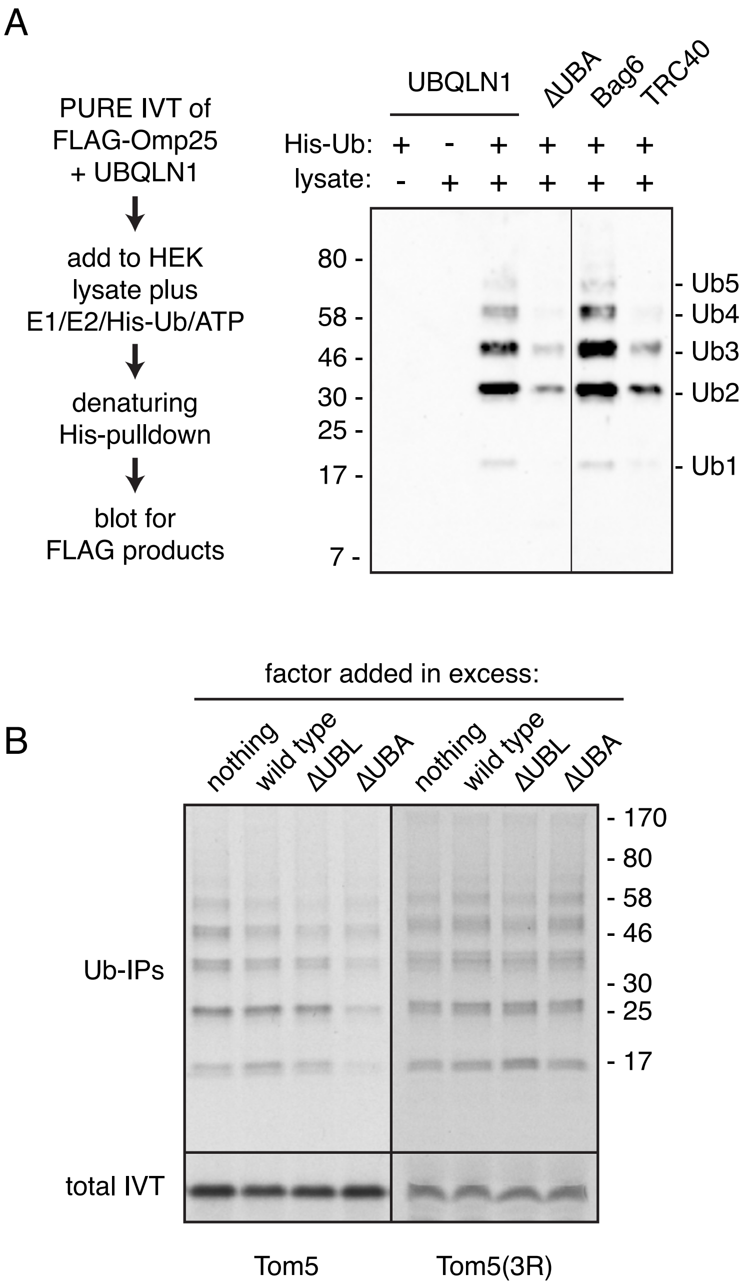

Fig. S6 (related to Fig. 4). Ubiquitination of Omp25 in complex with UBQLN1. (**A**) The left side shows the experimental strategy, and the right side the result. A FLAG-tagged construct containing the Omp25 TMD region was translated in the PURE system supplemented with recombinant UBQLN1 at 15 μM. The resulting UBQLN1-Omp25 complex was added to HEK293 cytosol supplemented with His-tagged Ubiquitin, E1 and E2 enzymes, and ATP. After incubation for 1 h, the ubiquitinated products were recovered via the His tag, and the samples immunoblotted for the FLAG tag. Note that lanes 1-4 were digitally spliced with lanes 5 and 6 to prepare the image. All samples were analyzed together on the same gel and the composite image was produced from the same exposure of the blot. Recovery of ubiquitinated Omp25 is observed to be dependent on both the lysate and His-Ubiquitin. The use of ΔUBA-UBQLN1 or TRC40 as the chaperone for Omp25 results in lower levels of ubiquitination, while Bag6 promotes client ubiquitination as expected from earlier studies. These results suggest that UBQLN1 is able to facilitate ubiquitination of its bound client in a UBA domain-dependent manner. (**B**) The UBQLN-associating client Tom5 and the non-associating Tom5(3R) mutant were translated in RRL containing ^{35}S-methionine, the indicated UBQLN1 protein (at 1 μM), and His-tagged Ubiquitin (at 10 μM). Shown are aliquots of the total translation products and the ubiquitinated products isolated via His-tagged Ubiquitin.

Itakura, Fig. S7 (related to Fig. 5)

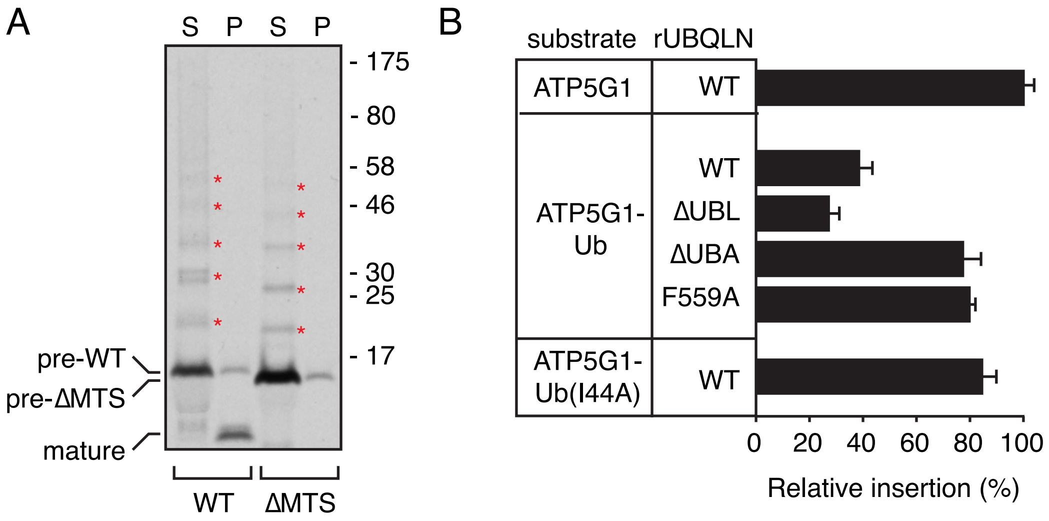

Fig. S7 (related to Fig. 5). Ubiquitinated ATP5G1 is prevented from membrane insertion. (**A**) Wild type (WT) and ΔMTS mutant ATP5G1 were produced in RRL containing ^{35}S-methionine, incubated with isolated mitochondria, separated by centrifugation into cytosolic supernatant (S) and mitochondrial pellet (P) fractions, and analyzed by SDS-PAGE and autoradiography. The positions of the precursor and mature forms of the proteins are indicated. Red asterisks indicate ubiquitinated species. Note that while non-ubiquitinated ATP5G1 is imported into mitochondria as judged by its processing and co-sedimentation, ubiquitinated ATP5G1 remains entirely in the cytosol. (**B**) The indicated substrates were translated in RRL supplemented with the indicated recombinant UBQLN1 proteins (at 1 μM) and analyzed for insertion into the mitochondria of semi-permeabilized cells. The graph shows the relative insertion efficiencies, normalized to that observed for reactions of ATP5G1 containing wild type UBQLN1 (mean ± SD).

Table S1 (related to Experimental Procedures) – List of constructs used in this study.

Construct Name (internal reference)	Purpose	Epitope tag	Functional element	Figure(s)
FH-Omp25 (EI-54)	IVT (RRL)	Tandem 3xFLAG-3xHA @ N-term.	Human Omp25 TMD region	1A, 1B, 5A
FH-Omp25ΔTM (EI-61)	IVT (RRL)	Tandem 3xFLAG-3xHA @ N-term.	Omp25 TMD region with hydrophobic region deleted	1A, 1B, 5A
FH-Omp25(3R) (EI-58)	IVT (RRL)	Tandem 3xFLAG-3xHA @ N-term.	Omp25 TMD region with 3R mutation	1B
FLAG-Omp25 (EI-71)	IVT (RRL)	3xFLAG @ N-term.	Omp25 TMD region	1C, 4C, S2D
FLAG-Omp25(3R) (EI-108)	IVT (RRL)	3xFLAG @ N-term.	Omp25 TMD region with 3R mutation	4C, S2D
β-Omp25 (SS)	IVT (RRL)	Sec61β-cytosolic domain @ N-term.	Omp25 TMD region	S1C, S1D
β-Omp25ΔTM (SS)	IVT (RRL)	Sec61β-cytosolic domain @ N-term.	Omp25 TMD region with hydrophobic region deleted	S1C, S1D
FLAG–β (4010)	IVT (RRL)	3xFLAG + Sec61β-cytosolic domain @ N-term.	Sec61β TMD region	1C
FLAG–β-VAMP2 (SS)	IVT (RRL)	3xFLAG + Sec61β-cytosolic domain @ N-term.	VAMP2 TMD region	1C
HA-Omp25 (EI-77)	IVT (RRL)	3xHA @ N-term.	Omp25 TMD region	S1B
HA-Omp25ΔTM (EI-78)	IVT (RRL)	3xHA @ N-term.	Omp25 TMD region with hydrophobic region deleted	S1B
HA-Sec61β (4009)	IVT (RRL)	3xHA @ N-term.	Human Sec61β	S1B
FLAG-Tom5 (EI-138)	IVT (RRL)	3XFLAG @ N-term.	Human Tom5 TMD region	1C, S6B
FLAG-Tom5(3R)	IVT (RRL)	3XFLAG @ N-term.	Human Tom5 TMD region with 3R mutation (changes residues ALLRVT with ARRRVT in TMD)	S6B
ATP5G1-FLAG (EI-186)	IVT (RRL)	3XFLAG @ C-terminus	Human ATP5G1	S2C, S2D, S7A
ATP5G1(ΔTM)-FLAG (EI-187)	IVT (RRL)	3XFLAG @ C-terminus	Human ATP5G1 with both TMDs deleted (residues 70-135)	S2D
ATP5G1(ΔMTS)-FLAG (EI-188)	IVT (RRL)	3XFLAG @ N-terminus in place of the MTS	Human ATP5G1 with N-terminal MTS disrupted (residues 1-25)	S2C, S2D, S7A
ATP5G1-HA	IVT (RRL)	3xHA @ C-terminus	Human ATP5G1	S7B
ATP5G1-Ub-HA	IVT (RRL)	3xHA @ C-terminus	Human ATP5G1 containing Ubiquitin(G76V) between ATP5G1 and the 3xHA tag.	S7B
ATP5G1-Ub(I44A)-HA	IVT (RRL)	3xHA @ C-terminus	Human ATP5G1 containing Ubiquitin(144A;G76V) between ATP5G1 and the 3xHA tag.	S7B
AcGFP (EI-190)	IVT (RRL)	AcGFP	none	S2D
MTS-AcGFP (EI-189)	IVT (RRL)	AcGFP	Mitochondrial targeting sequence (MTS) from cytochrome B @ N-terminus	S2D
HA-β-Omp25 (EI-197)	IVT (RRL)	3xHA + Sec61β cytosolic domain @ N-term.	Omp25 TMD region	4B, 5B, 7C, S2E
HA-Ub-β-Omp25 (EI-225)	IVT (RRL)	3xHA + Sec61β cytosolic domain @ N-term.	Omp25 TMD region plus Ubiquitin between HA and Sec61β domains	5D, 5F, 7C
HA-Ub(I44A)-β-Omp25 (EI-226)	IVT (RRL)	3xHA + Sec61β cytosolic domain @ N-term.	Omp25 TMD region plus Ubiquitin(I44A) between HA and Sec61β domains	5E, 5F
Sec61β (2935)	IVT (RRL)	None	Wild type human Sec61β	S2B
Sec61β(Δ2) (3554)	IVT (RRL)	None	Two residues deleted from TMD of Sec61β See Mariappan et al., 2010	S2B
Sec61β(Δ4) (3555)	IVT (RRL)	None	Four residues deleted from TMD of Sec61β See Mariappan et al., 2010	S2B
Sec61β(Δ6) (3556)	IVT (RRL)	None	Six residues deleted from TMD of Sec61β See Mariappan et al., 2010	S2B
Sec61β(Δ8) (3557)	IVT (RRL)	None	Eight residues deleted from TMD of Sec61β See Mariappan et al., 2010	S2B
Sec61β(2A) (3558)	IVT (RRL)	None	Two residues of Sec61β TMD changed to Alanine See Mariappan et al., 2010	S2B
Sec61β(4A) (3559)	IVT (RRL)	None	Four residues of Sec61β TMD changed to Alanine See Mariappan et al., 2010	S2B
Sec61β(6A) (3560)	IVT (RRL)	None	Six residues of Sec61β TMD changed to Alanine See Mariappan et al., 2010	S2B
FLAG-Omp25 (EI-117)	IVT (PURE)	3xFLAG @ N-term.	Omp25 TMD region	1D, S6A
HA-Omp25 (EI-118)	IVT (PURE)	3xHA @ N-term.	Omp25 TMD region	5C
HA-β-Omp25 (EI-203)	IVT (PURE)	3xHA + Sec61β-cytosolic domain @ N-term.	Omp25 TMD region	6B
HA-Ub-β-Omp25 (EI-227)	IVT (PURE)	3xHA + Sec61β-cytosolic domain @ N-term.	Omp25 TMD region plus Ubiquitin between HA and Sec61β domains	6C
HA-Ub(I44A)-β-Omp25 (EI-228)	IVT (PURE)	3xHA + Sec61β-cytosolic domain @ N-term.	Omp25 TMD region plus Ubiquitin(I44A) between HA and Sec61β domains	6C
GFP (F2)	Cell expression	Monomeric EGFP (originally from Clontech)	n/a	2E
GFP-Omp25 (EI-113)	Cell expression	Monomeric EGFP	Omp25 TMD region	2E, S3E
GFP-Omp25ΔTM (EI-174)	Cell expression	Monomeric EGFP	Omp25 TMD region with hydrophobic region deleted	2E
GFP-Omp25(3R) (EI-167)	Cell expression	Monomeric EGFP	Omp25 TMD region with 3R mutation	S3E
ATP5G1-HA	Cell expression	3xHA @ C-terminus	ATP5G1	2A, 2B, 2C, 2D,

(EI-156)				2E, S3B-S3D
GFP-ATP5G1ΔMTS-2A-RFP (EI-210)	Cell expression	GFP @ N-terminus; 2A-separated RFP (mCherry variant).	ATP5G1 with its MTS deleted (and replaced by GFP)	3B, 3E, 3F, S5A, S5B, S5F
GFP-2A-RFP (KY)	Cell expression	GFP @ N-terminus; 2A-separated RFP (mCherry variant).	n/a	3B
CFP-HttQ25 (EZ)	Cell expression	CFP (Cerulean variant) at C-terminus.	Human Htt exon1 containing 25 glutamine codons (mixture of CAA and CAG codons) at polymorphic site.	3D, 3E, S5C, S5D, S5E
CFP-HttQ105 (EZ)	Cell expression	CFP (Cerulean variant) at C-terminus.	Human Htt exon1 containing 105 glutamine codons (mixture of CAA and CAG codons) at polymorphic site.	3C, 3D, 3E, S5C, S5D, S5E
BFP-HttQ25 (EZ)	Cell expression	BFP (mTagBFP variant) at C-terminus.	Human Htt exon1 containing 25 glutamine codons (mixture of CAA and CAG codons) at polymorphic site.	S5F
BFP-HttQ105 (EZ)	Cell expression	BFP (mTagBFP variant) at C-terminus.	Human Htt exon1 containing 105 glutamine codons (mixture of CAA and CAG codons) at polymorphic site.	3F, S5F
pX330ΔCas9 (EI-154)	sgRNA cassette	n/a	pX330 from Ran et al. (2013) modified to excise the spCas9 expression cassette.	n/a
pX330Δ-UBQLN1 (EI-93)	sgRNA expression	n/a	pX330ΔCas9 plasmid with sgRNA that targets human UBQLN1 (CGAGAATAGCTCCGTCCAGC)	2A-2E, S3A, 3B, 3C, S5A, S5B, S5E, S4
pX330Δ-UBQLN2 (EI-98)	sgRNA expression	n/a	pX330ΔCas9 plasmid with sgRNA that targets human UBQLN2 (CGCGGGAACTAACACTACCT)	2A-2E, S3A, 3B, 3C, S5A, S5B, S5E, S4
pX330Δ-UBQLN4 (EI-101)	sgRNA expression	n/a	pX330ΔCas9 plasmid with sgRNA that targets human UBQLN4 (CTCTTGATCACAGTTCAAAG)	2A-2E, S3A, 3B, 3C, S5A, S5B, S5E, S4
pCDNA5-Frt-TO-FLAG-spCas9 (EI-128)	Cell expression	3xFLAG tag at N-terminus	Humanized S. pyogenes Cas9 in plasmid for stable-inducible expression in HEK293-Flp-in-TREX cells.	2A-2E, S3A, 3B, S5A, S5B, S4
pCDNA5-Frt-TO-Myc-UBQLN1 (EI-141)	Cell expression	3xMyc tag at N-terminus	Human UBQLN1 with 3xMyc at N-terminus in plasmid for stable-inducible expression in HEK293-Flp-in-TREX cells.	2B-2E, S3A, 3B, 3C, S5A, S5B, S5E, S4
pCDNA5-Frt-TO-Bag6-3C-FLAG (3444)	Cell expression	3C-FLAG tag at C-terminus	Human Bag6; See Hessa et al., 2011	S6A
His6-UBQLN1 (p24)	Bacterial expression	His6-tag at N-terminus	pCOLAduet vector containing human UBQLN1 tagged at N-terminus with His6-tag.	1D, S6A, 4C, S6B
His6-UBQLN1ΔUBL (p46)	Bacterial expression	His6-tag at N-terminus	pCOLAduet vector containing human UBQLN1ΔUBL tagged at N-terminus with His6-tag. Residues 2-107 deleted.	1D, 4C, S6B
His6-UBQLN1ΔUBA (p47)	Bacterial expression	His6-tag at N-terminus	pCOLAduet vector containing human UBQLN1ΔUBA tagged at N-terminus with His6-tag. Residues 547-589 deleted.	1D, S6A, 4C, S6B
His6-UBQLN1ΔM (p59)	Bacterial expression	His6-tag at N-terminus	pCOLAduet vector containing human UBQLN1 tagged at N-terminus with His6-tag. Residues 182-251 and 387-470 deleted.	1D, 4C
His6-3C-FLAG-UBQLN1 (EI-213)	Bacterial expression	His6-3C-3xFLAG at N-terminus	pCOLAduet vector containing human UBQLN1 tagged at N-terminus with His6-3C-FLAG sequence.	4B, 5C, 5D, 5E, 5F, 6, 7A, 7C, S7B
His6-3C-FLAG-UBQLN1ΔUBL (EI-214)	Bacterial expression	His6-3C-3xFLAG at N-terminus	pCOLAduet vector; human UBQLN1 tagged at N-terminus with His6-3C-FLAG sequence. Residues 2-107 deleted.	4B, 5F, 7A, 7C, S7B
His6-3C-FLAG-UBQLN1ΔUBA (EI-215)	Bacterial expression	His6-3C-3xFLAG at N-terminus	pCOLAduet vector; human UBQLN1 tagged at N-terminus with His6-3C-FLAG sequence. Residues 547-589 deleted.	4B, 5D, 5F, 7A, 7C, S7B
His6-3C-FLAG-UBQLN1(F559) (EI-229)	Bacterial expression	His6-3C-3xFLAG at N-terminus	pCOLAduet vector; human UBQLN1 tagged at N-terminus with His6-3C-FLAG sequence. F559A mutation in UBA domain.	5F, S7B
GST-3C-SGTA (3685)	Bacterial expression	GST-3C at N-terminus	Human SGTA. See Mateja et al., 2015	1D, 5C
His6-TEV-TRC40 (3567)	Bacterial expression	His6-TEV at N-terminus	Zebrafish TRC40	S6A
His6-HA-CaM (3621)	Bacterial expression	His6 and HA tags at N-terminus	Human Calmodulin; See Shao and Hegde, 2011	6

Extended Experimental Procedures

Plasmids, antibodies, and proteins

A description of all plasmids is provided in Table S1, along with the figure panels in which each was used. Plasmids for in vitro translation (IVT) in reticulocyte lysate (RRL) were based on the SP64 vector (Promega). Plasmids for IVT in the PURE system (Shimizu and Ueda, 2010) were based on the PURExpress DHFR Control Template provided by New England Biolabs. The DHFR open reading frame was replaced with the desired open reading frames. Plasmids for substrate expression in mammalian cells were based on either pCDNA3.1 (Invitrogen) or pEGFP (originally from Clontech) vectors, both of which use the CMV promoter.

Plasmid pX330 (Addgene 42230; Ran et al., 2013) was modified to remove the Cas9 expression cassette. This was then used for cloning of the desired sgRNA (chosen using the CRISPR design tool at crispr.mit.edu) to make knockout cell lines using CRISPR as described below. For transfection, the relevant expression cassettes for sgRNAs were PCR amplified using primers GAGGGCCTATTTCCCATGATTCC and CGGGCCATTTACCGTAAGTTAT and purified. The expression plasmid for FLAG-Cas9 was constructed by taking the FLAG-Cas9 open reading frame from plasmid pX330 (Ran et al., 2013) and cloned into the pCDNA5-Frt-TO vector. Myc-UBQLN1, used for rescue of the UBQLN knockout cell line, was cloned into the pCDNA5-Frt-TO vector.

The highly homologous UBQLN1 and UBQLN2 were detected simultaneously in blots using anti-UBQLN2 (from Sigma, clone 5F5, used at 1:1000 dilution). Control experiments using recombinant UBQLN1 and UBQLN2 verified that this antibody detects both antigens (data not shown). Anti-UBQLN4 was from Abcam (ab106443) and used at 1:1000 dilution. Rabbit polyclonal antibodies to Bag6, TRC40, SGTA, TRAPα, GFP, RFP have been described (Fons et al., 2003; Mariappan et al., 2010; Hessa et al., 2011). Anti-HA used for IPs and blots (e.g., Fig. 2A) was raised in rabbits against the KLH-HA peptide conjugate. Anti-L9 ribosomal protein was from Santa Cruz Biotech (T-17). The anti-Myc epitope tag (clone 9E10) was produced as culture supernatant from the corresponding hybridoma. Anti-HA was from Covance (Clone 16B12). Antibodies to proteasomal proteins Rpt5 and α7 were from Abcam (ab22635) and Enzo Life Sciences (PW8110), respectively. Anti-Tom20 was from Santa Cruz (FL-145). Anti-FLAG-M2 affinity resin and anti-FLAG-M2 antibody were from Sigma. GFP-trap was from Chromtek. Additional commercial antibodies used in Fig. 4A were: Hsc/Hsp70 (AssayDesigns, SPA-822), Hsp60 (Abcam ab46798), ClpP (Abcam ab124822), Actin-HRP (Sigma A3854), OxPhos cocktail (Abcam ab110411).

Recombinant His6-Ubiquitin, GST-Ube1 (E1 enzyme), and UbcH5a (E2 enzyme) were from Boston Biochem. Human UBQLN1 was tagged at the N-terminus with either a His6 tag or a tandem His6-3C-FLAG tag (where 3C is the cleavage site for 3C protease) and cloned into the pCOLA duet vector. The plasmids were introduced into BL21(DE3) LOBSTR strain of *E. coli* (Andersen et al., 2013), and induced with 0.1 mM IPTG at 16°C overnight. The cells were sedimented and washed in lysis buffer (PBS supplemented with 300 mM NaCl and 10 mM imidazole), resuspended in lysis buffer, and disrupted by sonication. The insoluble material was removed by centrifugation, and the soluble fraction was adjusted to 0.1% Triton X-100 before passing over a column of Co^{2+}-charged chelating sepharose (GE Biosciences). The column was washed with PBS containing 0.1% Triton X-100 and 500 mM NaCl, then extensively in PBS

plus 500 mM NaCl without detergent, and eluted with PBS plus 150 mM NaCl and 200 mM imidazole. The His tag on His-3C-Flag-UBQLN1 was cleaved by 3C protease, and the tag, protease, and uncleaved products were removed by passing over Co^{2+}-charged chelating sepharose resin. Recombinant TRC40, SGTA, and Bag6 were produced as previously described (Rodrigo-Brenni et al., 2014; Mateja et al., 2015).

Cell culture

HeLa and Flp-in T-Rex HEK293 cells (Invitrogen) were cultured in DMEM supplemented with 10% fetal bovine serum, 50 μg /ml penicillin/streptomycin in a humidified 5% CO_2 incubator. HEK293 Flp-in T-Rex cells were maintained in the presence of 15 μg/ml blasticidin and 100 μg/ml zeocin. Flag-Cas9, UBQLN triple knockout and UBQLN1 rescued cells (produced as described below) were maintained in the presence of 10 μg/ml blasticidin and 100 μg/ml hygromycin. 10 ng/ml doxycycline was used for induction of the integrated gene at the FRT site. Cell growth assay (Fig. 4C) was performed by passaging the cells into replicate 24 well plates and viable cells counted by trypan blue staining on each day for four days. For Fig. 4D, one hundred cells were passaged into 6-well dishes, cultured for 14 days, fixed in 3.7% formaldehyde in PBS for 30 min, and stained with 0.5% crystal violet in 10% methanol for 20 min. After washing in water, the cells were dried and photographed.

Microscopy

Images in Fig. 3C, 3F, and S5B, were acquired on a Nikon TE2000 fluorescence microscope with a 100x/1.49NA Oil objective. Fig. 3C and S5B show epifluorescence images. For Figure 3F, the specimen was imaged as a z-stack, deconvoluted using Huygens Software by Scientific Volume Imaging, and displayed as a maximum intensity projection with Fiji software. For Fig. 3F and S5B, samples were simply fixed and the fluorescent proteins imaged, while for Fig. 3C the sample was fixed, permeabilized, stained using anti-Myc antibody, and mounted prior to imaging.

Pulse-chase analysis

Analysis of cultured cells by pulse-chase labeling was essentially as described before (Ashok and Hegde, 2009, and references therein). Cells were typically transfected 24 h before initiating the experiment. Prior to labeling, cells were starved for 15 min at 37°C in media lacking methionine. Labeling was initiated by addition of ^{35}S-methionine to a final concentration of between 100 and 200 μCi per ml, depending on the experiment. After the intended labeling period, excess unlabeled methionine (to 2 mM final concentration) was added to the media, immediately after which the pulse labeled sample (i.e., the 0 min chase time point) was harvested. Subsequent time points were harvested in the same way (see below). Where indicated, a mixture of CCCP and Valinomycin (2 μM and 1 μM final concentrations, respectively) was included throughout (beginning with the starvation) to acutely inhibit mitochondrial import. Where MG132 was used for proteasome inhibition, it was added to a final concentration of 10 μM 90 min prior to beginning the starvation and maintained throughout the pulse and chase.

Cells were harvested in different ways depending on the downstream application. For immunoprecipitation of labeled products (e.g., Fig. 2A, 2B, S3B, and S3D), media was removed from the cells and they were directly solubilized in 1% SDS, 0.1 M Tris, pH 8. The samples were heated to 95°C, vortexed extensively and/or passed through narrow gauge needles to shear the

DNA, and cooled before dilution in immunoprecipitation (IP) buffer (150 mM NaCl, 50 mM Hepes, pH 7.4, 1% Triton X-100). For analysis of solubility (Fig. 2D), media was removed from the cells and the cells lysed in ice cold lysis buffer (150 mM NaCl, 50 mM Hepes, pH 7.4, 1% Triton X-100). The cell lysate was then passed 10 times through a 26 gauge needle. After centrifugation at 15,000 rpm for 30 min at 4°C, the supernatant was reserved in a separate tube and the pellet was dissolved directly in 1% SDS, 0.1 M Tris, pH 8.0. The genomic DNA for the pellet fraction was sheared by boiling and repeated vortexing, and the supernatant and pellet fractions analyzed either directly for autoradiography, subjected to immunoprecipitation, or subjected to immunoblotting.

In vitro transcription and translation

IVT reactions in the RRL system were performed by minor modifications of published methods (Sharma et al., 2010). SP6-based transcription utilized PCR products as templates and were for 1 hour at 37°C. Translation utilized RRL, phenyl-depleted RRL (Fig. S2E), DEAE-fractionated RRL (Fig. 4B), or phenyl-depleted and DEAE fractionated RRL (Fig. 7C) as previously described (Sharma et al., 2010; Hessa et al., 2011; Shao and Hegde, 2011). Where indicated in the Figure legends, recombinant proteins were added to the translation reactions, typically to 1 μM final concentration. Reactions were typically for 30 min at 32°C.

IVT in the PURE system was essentially as described (Shimizu and Ueda, 2010) and utilized either the PURExpress Kit (NEB) or in-house reagents prepared according to Shimizu and Ueda (2010). Where indicated, ^{35}S methionine and the indicated recombinant chaperone (at 15 μM final concentration) were included. Translation reactions were for 2 hours at 37°C. For site-specific photo-crosslinker incorporation (Fig. 6), the open reading frame was modified to have the UAG amber codon at the desired location and the UAA codon at the desired termination site. Translation reactions were supplemented with total tRNA isolated from *E. coli* expressing the amber suppressor (final A260 of 25), recombinant synthetase for charging the suppressor tRNA (50 μg/ml; Chin et al., 2002), and BpF (100 μM). A series of control experiments verified that all three components were required to obtain read-through of amber codons (data not shown), as expected from the well-documented orthogonality of this system in *E. coli* (Chin et al., 2002).

Sucrose gradient fractionation

0.2 ml gradients were prepared in 7 x 20 mm centrifuge tubes (Beckman 343775) by successively layering 40 μl each of 25%, 20%, 15%, 10%, and 5% sucrose (w/v) in KHM buffer (110 mM KAc, 20 mM Hepes, pH 7.4, 2 mM $MgAc_2$) and allowed to diffuse for 1-2 h at 4°C. Translation reactions (either 20 μl of RRL translations or 10 μl PURE reactions diluted with 10 μl KHM buffer) were loaded on top of the gradients, and the samples centrifuged in a TLS-55 rotor (with appropriate tube adaptors) at 55,000 rpm for 2.3 hours at 4°C with the slowest acceleration and deceleration settings. Eleven 20 μl fractions were successively collected from the top and used for downstream assays as indicated in the individual figure legends. The eleventh fraction was mixed with any pelleted materials. In some experiments, 10-fold larger gradients were prepared in exactly the same way using 11 x 34 mm tubes (Seton 5011), but centrifuged at 55,000 rpm in the TLS-55 rotor for 5 h. When fractionated into eleven fractions, the migration of products corresponds precisely to that seen in the smaller volume gradients.

Affinity purification

Affinity purification of UBQLN-client complexes was performed in the absence of detergent, which potently disrupts the interaction. In vitro translation reactions prepared as indicated in the individual figure legends, were added to pre-washed Anti-FLAG-M2 agarose or GFP-trap (typically 10 μl resin volume) and gently rotated for 2 hours at 4°C. The beads were washed between 4 to 6 times with 1 ml KHM buffer each. In some experiments, the beads were transferred to a new tube after the penultimate wash to reduce background. Bound proteins were eluted with SDS-PAGE sample buffer, or in some cases (e.g., Fig. 1A, 4B, and 7A), the FLAG peptide (added to 0.2 mM for 30 min at 20°C).

For affinity purification of UBQLN-client complexes from cells (Fig. 2C), WT and Myc-UBQLN1 rescue cells transfected with ATP5G1-HA were employed. After pulse-labeling with ^{35}S-methionine for 30 min, one-fifth of the cells was set aside for denaturing IPs of the ATP5G1-HA client to verify equal levels of expression. The remaining cells were chilled rapidly to between 0-4°C on ice, sedimented, and flash-frozen on dry ice. All further manipulations carried out with samples on ice in a cold room to minimize the otherwise rapid dissociation of client from UBQLN1 (e.g., Fig. 6B). The cells were thawed by addition of ice-cold lysis buffer (125 mM KAc, 50 mM Hepes, pH 7.4), dispersed by passage 7-8 times through a 26 gauge needle, and lysed by passage three times through a 30 gauge needle. The insoluble material was removed by centrifugation for 10 min at maximum speed in a microcentrifuge, and the supernatant added to ~5 μl immobilized anti-Myc monoclonal antibody (clone 9E10). After binding for 2 h at 4°C, the resin was washed three times in 80 volumes lysis buffer each, transferred to new tubes, and washed once more. After removing residual lysis buffer, the clients were eluted on ice with 1% Triton X-100, 150 mM NaCl, and 50 mM Hepes, pH 7.4. After removing the eluate, the resin was eluted a second time with SDS-PAGE sample buffer. One half of the Triton X-100 eluate was then subjected to re-immunoprecipitation with anti-HA resin after denaturation in SDS. Aliquots of all fractions and IPs were analyzed by SDS-PAGE and autoradiography.

Targeting assays in semi-intact cells

For microscopy-based targeting assays, HeLa cells grown on glass coverslips (13 mm diameter) were washed with ice-cold KHM buffer and treated with 50 μg/ml digitonin in KHM for 5 min at 4°C. After washing once in KHM buffer, the coverslips were placed in a humidified chamber at 30°C and overlaid with either 30 μl of the RRL translation reaction or 1 μl of the PURE system reaction diluted to 30 μl with KHM buffer. PURE IVT reactions and sucrose gradient fractions were supplemented with 3% BSA (to minimize non-specific binding) and an ATP regeneration system. Incubation was for 15 min (for RRL IVT) or 30 min (for PURE IVT). The coverslips were then washed with PBS twice and fixed in 3.7% formaldehyde in PBS for 15 min at room temperature. Fixed cells were permeabilized with 0.1% Triton X-100 in PBS for 5 min, blocked with 10% fetal bovine serum in PBS for 30 min, and incubated with primary antibodies in blocking buffer for 1 hour. After washing with PBS three times, cells were incubated with AlexaFluor 488-conjugated goat anti-mouse IgG and/or AlexaFluor 564-conjugated goat anti-rabbit IgG secondary antibodies (Invitrogen) in blocking buffer for 60 min. Images were acquired on a confocal laser scanning microscope (LSM 780, Zeiss) using a 63×/1.42NA oil-immersion objective.

For targeting assays using sedimentation, HEK293 cells (from a 10 cm dish at 50% confluence) were detached by pipetting with PBS and transferred to a 1.5 ml microcentrifuge tube. After centrifugation at 5000 rpm for 2 min, the supernatant was removed and the cells were resuspended in 50 μg/ml digitonin in KHM buffer and incubated for 5 min at 4°C. The cells were washed with KHM buffer 2 times and the semi-intact cells were resuspended in 500 μl KHM at 4°C. Twenty μl of the semi-intact cells were mixed with 10 μl of the RRL-based translation reaction and incubated for 30 min at 32°C. The reactions were then centrifuged at 2,348 x g for 5 min. The supernatant was saved and the pellet was washed with KHM 2 times, sedimenting the cells each time at 9,391 x g for 5 min. The cell fraction was treated with 1 U DNAse per 30 μl to digest genomic DNA. The supernatant and cell fractions were denatured in SDS-PAGE sample buffer prior to downstream analysis.

In vitro import into isolated mitochondria

Isolation of yeast mitochondria was as described (Meisinger et al., 2006), using the BY4741 strain. Import assays contained a 10:1 volume ratio of RRL translation reaction to mitochondria (A_{280} of ~30). Incubation was at 25 °C for 15 or 30 min. Reactions were directly analyzed (for signal cleavage) or centrifuged through a 15% sucrose cushion in 100 mM KAc, 50 mM Hepes, pH 7.4, 2 mM $MgCl_2$, in a microcentrifuge at 4 °C for 15 min at maximum speed. Equal amounts of supernatant and pellet samples were then directly analyzed by SDS-PAGE and autoradiography.

Generation of UBQLN triple knockout cells

To facilitate CRISPR/Cas9 mediated gene disruption, we first prepared stable inducible FLAG-Cas9 expressing cells by targeting the FLAG-Cas9 expression cassette to the FRT locus. Briefly, FLAG-Cas9 in the pCDNA5 Frt-Tet-On plasmid was co-transfected with the FLP recombinase expressing plasmid pOG44 (Invitrogen) into HEK293 Flp-in TRex cells and positive integrants were selected by resistance to 100 μg/ml hygromycin. These cells were then co-transfected with purified PCR products containing the sgRNA expression cassettes to target UBQLN1 (CGAGAATAGCTCCGTCCAGC), UBQLN2 (CGCGGGAACTAACACTACCT), and UBQLN4 (CTCTTGATCACAGTTCAAAG). FLAG-Cas9 expression was induced with 10 ng/ml doxycycline for 5 days after sgRNA transfection. Single cell clones were isolated by dilution into 96-well plates followed by culturing for 2 weeks, and screened for successful knockout of all three UBQLNs by immunoblotting relative to serial dilutions of parental cells.

To generate the UBQLN1 rescue cell line, the FLAG-Cas9 at FRT site of the UBQLN triple KO cells was removed by transient expression of the FLP recombinase using the pOG44 vector and selected for resistance to Zeocin. The restored FRT site was then re-targeted with either the empty pCDNA5-FRT-TO vector or pCDNA5-FRT-TO containing the Myc-UBQLN1 open reading frame. After hygromycin selection, the cell line transfected with the empty vector was used as the UBQLN triple knockout cell line, while the cell line transfected Myc-UBQLN1 was used as the rescue cell line. Induction of the rescue cell line with 10 ng/ml doxycycline resulted in Myc-UBQLN1 expression at levels comparable to total UBQLN1/2/4 expression in the parental HEK293 cells.

Detergent solubility assay

Cells in each well of a 6 well plate were mechanically lysed in 100 µl of lysis buffer (150 mM NaCl, 50 mM Tris pH. 7.4, 1mM EDTA, 0.5% Triton, 0.5% Deoxycholate, 1x Complete protease inhibitor cocktail, and 1 mM PMSF) and incubated on ice for 30 min. After centrifugation at 15,000 rpm for 30 min at 4°C, the supernatant was reserved in a separate tube and the pellet was washed once with lysis buffer, recovered by centrifugation, and resuspended in 100ul lysis buffer. Both the soluble and insoluble fractions were denatured in SDS-PAGE sample buffer for downstream analysis. Where necessary, the genomic DNA in the insoluble fraction was sheared by passing it several times through a 30-gauge needle. For Fig. 3D, S5A, and S5E, the cells were lysed in 150 mM NaCl, 50 mM Hepes, pH 7.4, 1% Triton X-100 and passed 10 times through a 26 gauge needle. After centrifugation at 15,000 rpm for 30 min at 4°C, the supernatant was reserved in a separate tube and the pellet was dissolved directly in SDS-PAGE sample buffer. The genomic DNA in was sheared by boiling and repeated vortexing.

Flow cytometry analysis

HEK293 cells transfected as indicated in the individual figure legends were recovered by detachment from the dish with EDTA and gentle pipetting. The cells were sedimented (5000 rpm for 2 min at 4°C) and resuspended in 10% fetal bovine serum in PBS for flow cytometry analysis using a Becton Dickinson LSRII analyzer. Sorting of aggregate-containing from aggregate-lacking cells was performed as described previously (Ramdzan et al., 2012) using a Sony iCyt Synergy sorter.

Analysis of ubiquitination

In experiments where ubiquitination in translation reactions was assessed (Fig. 4C, 5B, and S1C), 10 µM His-tagged Ubiquitin was added to the reaction. Ubiquitinated products were selectively recovered via the His tag by binding to Co^{2+}-charged chelating sepharose as described (Hessa et al., 2011). In reactions where ubiquitination was performed on affinity purified products (Fig. 4B) or PURE IVT reactions (Fig. S6A), the samples were supplemented with one-fourth volume of pre-assembled 4X Ubiquitination mix prepared by mixing 40 µM His-Ubiquitin, 0.4 µM GST-Ube1 (E1 enzyme), 1.2 µM UbcH5a (E2 enzyme), and an energy regenerating system (4 mM ATP, 40 mM creatine phosphate, 160 µg/ml creatine kinase). Reactions were incubated for 30 min at 37°C before termination by boiling in 1% SDS. The denatured products were diluted 10-fold in 50 mM HEPES pH7.4, 100 mM NaCl, 1% TritonX100 and the ubiquitinated products recovered by binding to Co^{2+}-immobilized chelating sepharose.

Miscellaneous

In most cases, samples were analyzed on 10 or 12% Tris-Tricine gels. Equal loading for SDS-PAGE, IPs, and targeting assays into semi-intact cells was controlled as follows. For immunoblotting experiments, equal loading was ensured by two means. First, the protein concentrations of cell lysates were measured and equal amounts were used for downstream analysis. Second, the blots were always stained for total protein (shown in the case of Fig. 2E and 3D as examples), verifying equal loading and recovery. In experiments where radiolabeled products were analyzed, the gels were always stained with coomassie blue to verify equal loading of IVT products or cell lysate as appropriate. In immunoprecipitation or pulldown experiments, equal amounts of samples were used to program the IP reaction, and equal recovery during the IP was always verified by staining of the gel to visualize IgG (or free ubiquitin in the

case of ubiquitin-pulldowns). For the pulse-chase experiment, small aliquots of the lysates for all samples were analyzed separately to verify equal rates of label incorporation. In instances where the IPs were eluted with peptide (e.g., Fig. 1A), the remaining beads were boiled and analyzed to verify equal recovery of IgG. For import reactions into semi-permeabilized cells, the amounts of translation products were quantified by using a small amount of ^{35}S-methionine as a tracer, and equal amounts of translated product were used in the assay.

Supplemental References

Andersen, K.R., Leksa,N.C., Schwartz, T.U. (2013) Optimized E. coli expression strain LOBSTR eliminates common contaminants from His-tag purification. Proteins *81*, 1857-61.

Fons, R.D., Bogert, B.A., Hegde, R.S. (2003) Substrate-specific function of the translocon-associated protein complex during translocation across the ER membrane. J Cell Biol.*160*, 529-39.

Meisinger, C., Pfanner, N., Truscott, K.N. (2006) Isolation of yeast mitochondria. Methods Mol Biol. *313*, 33-9.
