# Supplementary material for: Ubiquilins Chaperone and Triage Mitochondrial Membrane Proteins for Degradation
Source: Mol Cell. 2016 Jul 7;63(1):21–33. doi: 10.1016/j.molcel.2016.05.020 (PMC4942676; doi:10.1016/j.molcel.2016.05.020)
Supplement: Document S2. Article plus Supplemental Information [file mmc2.pdf]

Article

Molecular Cell

Ubiquilins Chaperone and Triage Mitochondrial Membrane Proteins for Degradation

Graphical Abstract

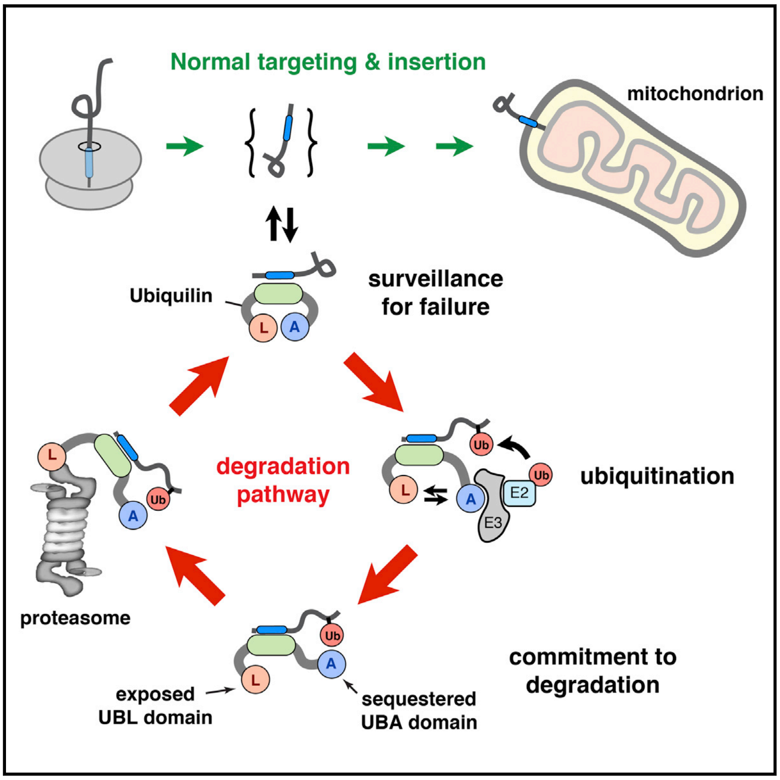

Highlights

- Ubiquilins are conserved cytosolic chaperones for transmembrane domains
- Mitochondrial membrane proteins that fail targeting are degraded by Ubiquilins
- The absence of Ubiquilins in cells leads to membrane protein precursor aggregation
- Interactions with Ubiquilins' UBA and UBL domains coordinate client degradation

Authors

Eisuke Itakura, Eszter Zavodszky, Sichen Shao, Matthew L. Wohlever, Robert J. Keenan, Ramanujan S. Hegde

Correspondence

rhegde@mrc-lmb.cam.ac.uk

In Brief

Membrane proteins require targeting to an intracellular organelle for insertion. When targeting fails, they must be recognized and degraded promptly to avoid aggregation. Itakura et al. show that Ubiquilins are cytosolic chaperones that prevent aggregation of mitochondrial membrane protein precursors, routing them for degradation if they linger uninserted in the cytosol.

Itakura et al., 2016, Molecular Cell *63*, 21–33
July 7, 2016 © 2016 MRC Laboratory of Molecular Biology.
Published by Elsevier Inc.
http://dx.doi.org/10.1016/j.molcel.2016.05.020

Molecular Cell
Article

CellPress

Ubiquilins Chaperone and Triage Mitochondrial Membrane Proteins for Degradation

Eisuke Itakura,[1,2] Eszter Zavodszky,[1] Sichen Shao,[1] Matthew L. Wohlever,[3] Robert J. Keenan,[3] and Ramanujan S. Hegde[1,*]
[1]MRC Laboratory of Molecular Biology, Francis Crick Avenue, Cambridge CB2 0QH, UK
[2]Department of Biology, Faculty of Science, Chiba University, 1-33, Yayoi-cho, Inage-ku, Chiba, 263-8522, Japan
[3]Department of Biochemistry and Molecular Biology, The University of Chicago, 929 East 57th Street, Chicago, IL 60637, USA
*Correspondence: rhegde@mrc-lmb.cam.ac.uk
http://dx.doi.org/10.1016/j.molcel.2016.05.020

SUMMARY

We investigated how mitochondrial membrane proteins remain soluble in the cytosol until their delivery to mitochondria or degradation at the proteasome. We show that Ubiquilin family proteins bind transmembrane domains in the cytosol to prevent aggregation and temporarily allow opportunities for membrane targeting. Over time, Ubiquilins recruit an E3 ligase to ubiquitinate bound clients. The attached ubiquitin engages Ubiquilin's UBA domain, normally bound to an intramolecular UBL domain, and stabilizes the Ubiquilin-client complex. This conformational change precludes additional chances at membrane targeting for the client, while simultaneously freeing Ubiquilin's UBL domain for targeting to the proteasome. Loss of Ubiquilins by genetic ablation or sequestration in polyglutamine aggregates leads to accumulation of non-inserted mitochondrial membrane protein precursors. These findings define Ubiquilins as a family of chaperones for cytosolically exposed transmembrane domains and explain how they use ubiquitin to triage clients for degradation via coordinated intra- and intermolecular interactions.

INTRODUCTION

Approximately 25% of protein-coding genes in all organisms encode integral membrane proteins. Although their final destination is in a lipid bilayer, they are synthesized by cytosolic machinery and transiently navigate the aqueous cytosol. Because the transmembrane domains (TMDs) in these proteins are effectively insoluble in aqueous environments, specialized machinery must recognize and shield them until their insertion. Several TMD-shielding factors have been identified and extensively studied for proteins destined for the endoplasmic reticulum (ER) (Cross et al., 2009; Hegde and Keenan, 2011). In contrast, the factors that interact with and maintain the solubility of mitochondrial membrane proteins during their transient residence in the cytosol are incompletely defined.

Mitochondria contain ~1,000–1,500 proteins. Nearly all mitochondrial proteins are imported from the cytosol (Neupert, 1997), and a large proportion of them contain TMDs. The import machinery at the outer and inner mitochondrial membranes has been extensively studied (Chacinska et al., 2009). However, the cytosolic factors that maintain import competence of mitochondrial precursors, prevent aggregation, and route them for degradation in the case of import failure are largely unknown. The general chaperones Hsp70 and Hsp90 are implicated in maintaining an unfolded state and targeting various precursors to the mitochondrial outer membrane (Young et al., 2003). However, these chaperones have client-binding clefts that are too small to effectively shield the long hydrophobic regions that typify many TMDs (Shiau et al., 2006; Zhu et al., 1996), and suitable alternative factors have not been identified.

TMD shielding is likely to be important not only for productive biogenesis, but also for degradation of failed insertion precursors. Failure of mitochondrial import can occur for a number of reasons. In addition to inherent inefficiencies that accompany any biological process (Wolff et al., 2014), import is under regulatory control and may be acutely inhibited under different physiologic conditions (Harbauer et al., 2014; Schmidt et al., 2011). Furthermore, mitochondrial stress can result in impaired import (Wright et al., 2001), and chronic failure of import is detrimental to cytosolic protein homeostasis (Wang and Chen, 2015; Wrobel et al., 2015). Thus, cells presumably have pathways to degrade mitochondrial precursors after their initial attempts at translocation or insertion fail. The factors involved in this type of quality control are not clear. As with biogenesis, membrane proteins pose a particularly difficult challenge due to their relative insolubility.

Nearly all available information about chaperones for TMDs in the cytosol comes from the study of targeting and degradation of proteins destined for the ER (Cross et al., 2009; Hegde and Keenan, 2011). Most ER membrane proteins are recognized co-translationally by the ribosome-associating signal recognition particle (SRP). The positioning of its TMD-binding domain at the ribosome exit tunnel ensures effective TMD shielding from solvent during targeting. Membrane proteins that fail to engage SRP encounter a different set of cytosolic TMD-binding factors that mediate post-translational targeting to the ER (Hegde and Keenan, 2011) or degradation at the proteasome (Hessa et al., 2011).

This is an open access article under the CC BY license (http://creativecommons.org/licenses/by/4.0/).

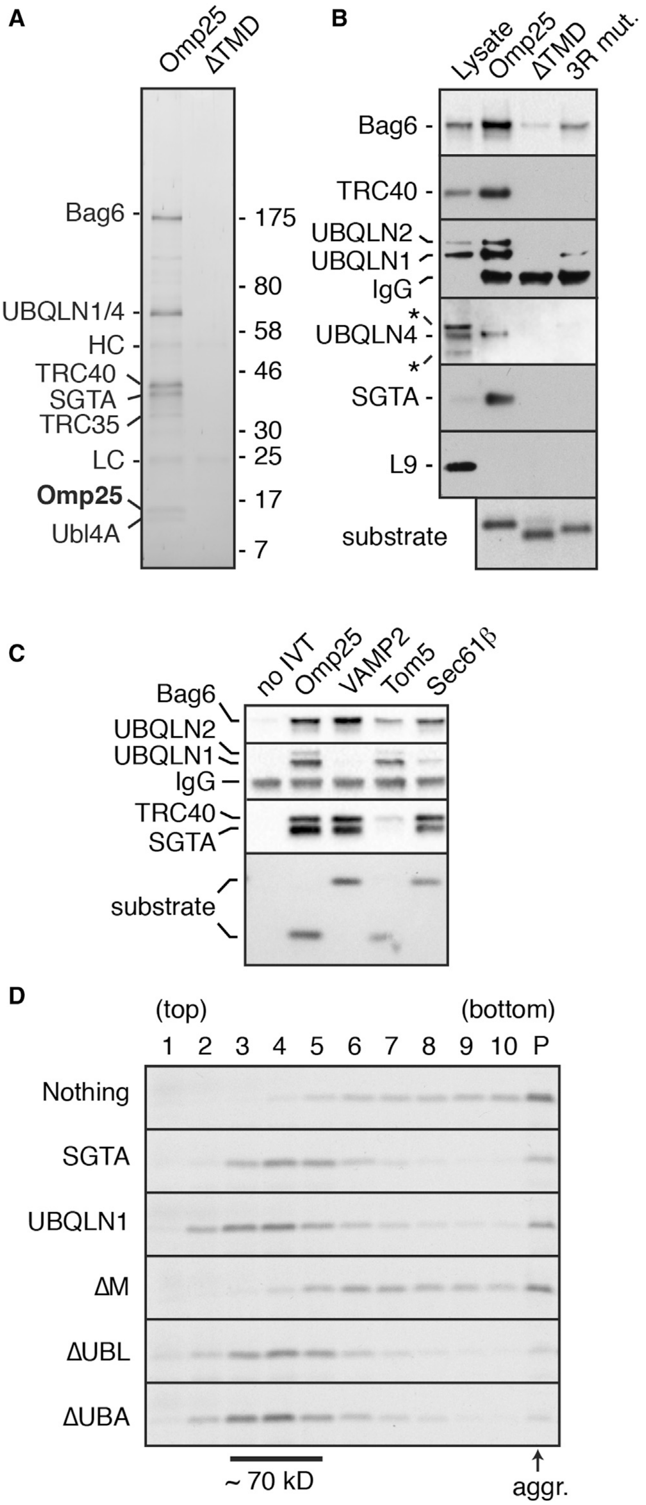

Figure 1. Ubiquilins Are Cytosolic TMD-Binding Chaperones

(A) FLAG-tagged constructs containing or lacking the Omp25 TMD (see Figure S1) were translated in RRL, affinity purified via the FLAG tag, separated by SDS-PAGE, and detected with SYPRO-Ruby stain. The identity of each band is indicated. HC and LC are immunoglobulin heavy and light chain, respectively.

(B) FLAG-tagged constructs containing the Omp25 TMD or the indicated TMD mutants were translated in RRL containing ^{35}S-methionine, affinity purified, and analyzed by immunoblotting relative to total RRL (first lane). The translated substrates were detected using autoradiography. Asterisks indicate non-specific products.

(C) FLAG-tagged constructs containing the TMD regions of the indicated proteins were analyzed for interaction partners as in (B).

(D) FLAG-tagged construct containing the Omp25 TMD was translated in the PURE translation system with ^{35}S-methionine and 15 μM of recombinant SGTA, UBQLN1, or the indicated UBQLN1 mutant. The reactions were separated by sucrose gradient, and the Omp25 visualized by autoradiography. SGTA (~70 kD native size) and each of the recombinant UBQLN1 proteins migrate primarily in fractions 3 to 5 (not shown).

See also Figures S1 and S2.

The major class of membrane proteins post-translationally targeted to the ER are tail-anchored (TA) proteins, named for their single C-terminal TMD (Hegde and Keenan, 2011). TA proteins must be loaded onto an ATPase named Get3 (TRC40 in mammals) for targeting to ER-resident receptors (Stefanovic and Hegde, 2007; Schuldiner et al., 2008). Loading onto Get3 requires transfer, via a bridging complex, from a "pre-targeting" chaperone called Sgt2 (Wang et al., 2010). Mammals appear to use homologous components, with the additional inclusion of Bag6 as a subunit of the bridging complex (Mariappan et al., 2010). Sgt2 (SGTA in mammals), Get3, and Bag6 are all TMD-binding proteins, thereby precluding aggregation of their hydrophobic clients. Of these, Bag6 appears to have broader specificity beyond TA proteins (Hessa et al., 2011) and can route its clients for degradation via recruitment of an E3 ubiquitin ligase (Rodrigo-Brenni et al., 2014).

Given the extensive machinery for ER membrane proteins, we anticipated the existence of analogous factors that operate on the equally challenging problem of mitochondrial membrane proteins. However, earlier searches for the simplified case of mitochondrial TA proteins (e.g., Krumpe et al., 2012; Setoguchi et al., 2006) did not reveal protein factors in either the cytosol or the membrane required for targeting, insertion, or degradation. We now report that Ubiquilins are general TMD chaperones for both TA and non-TA mitochondrial membrane proteins. Functional and biochemical studies indicate that the primary role of Ubiquilins is to triage their clients for proteasomal degradation in the instance of failed membrane targeting.

RESULTS

Ubiquilins Are Major TMD-Binding Proteins of the Cytosol

We initiated our studies using the outer mitochondrial TA membrane protein Omp25 (Figure S1A). We found that Omp25 synthesized in rabbit reticulocyte lysate (RRL) can be selectively targeted to mitochondria of semi-permeabilized cells (Figure S1B), but is ubiquitinated when insertion is precluded (Figure S1C). Both targeting and ubiquitination strictly depend on its TMD, which was observed to engage with factors in the translation extract as judged by the relatively large native size of Omp25 on sucrose gradient separations (Figure S1D). To find these putative factor(s), we affinity purified RRL-translated FLAG-tagged Omp25 and identified the major interaction partners using mass spectrometry (Figure 1A). In addition to the

TMD chaperones for ER-destined TA proteins (SGTA, TRC40, and the heterotrimeric Bag6 complex), we identified UBQLN1 and UBQLN4, two homologous members of the Ubiquilin (UBQLN) family of proteins.

Mammals have four UBQLNs with identical domain architecture and high sequence conservation (Figure S2A). They have an N-terminal ubiquitin-like (UBL) domain and a C-terminal ubiquitin-associating (UBA) domain. The middle region (hereafter termed the M domain) is poorly characterized, but has an unusually high methionine content similar to the TMD-binding domains of SRP (Bernstein et al., 1989), SGTA (Wang et al., 2010), and TRC40 (Mateja et al., 2009). This region contains predicted "Sti1-like" domains of uncertain relevance. Three of the UBQLNs (1, 2, and 4) are widely expressed, show between 68% and 83% sequence similarity to each other, and are specifically recovered in Omp25 immunoprecipitates (IPs) in a TMD-dependent (Figure 1B) and detergent-sensitive (data not shown) manner. The more distantly related testis-specific UBQLN3 was not identified with mass spectrometry or IP and was not pursued further.

Analysis of the interaction partners for a range of TMDs and mutants indicate that UBQLNs prefer moderately hydrophobic TMDs that typify many mitochondrial membrane proteins (Figures 1C and S2B). Hence, TMDs from the mitochondrial proteins Omp25, Tom5, and Bak engage UBQLNs more effectively than the ER-destined TMDs from Sec61β and VAMP2. ATP5G1, a two-TMD inner mitochondrial membrane protein containing a mitochondrial targeting signal (MTS) for import via the TOM and TIM complexes (Figure S2C; Chacinska et al., 2009), also interacted with UBQLNs in a TMD-dependent manner (Figure S2D). No interaction was seen with either the MTS or incomplete or disrupted TMDs. At this level of resolution, we have not observed major differences in the substrate specificity of the different UBQLN family members, so we focused our subsequent studies on UBQLN1.

To determine whether UBQLN1 can maintain solubility of its TMD-containing client, we used the "PURE" translation system reconstituted with recombinant *E. coli* translation factors (Shimizu and Ueda, 2010). Aggregation of newly synthesized Omp25 in this chaperone-free system was substantially prevented by the presence of recombinant UBQLN1 (Figure 1D). Analysis of UBQLN1 deletion mutants in this assay indicates that the UBA and UBL domains are dispensable for TMD binding, which is mediated by the central M domain (Figure 1D). The native size of the UBQLN1-Omp25 complex suggests that it contains a single UBQLN1, similar to the native UBQLN-Omp25 complexes observed in cytosol (Figure S2E).

Depletion of the major TMD-binding proteins from RRL by passage over phenyl-sepharose produced a translation extract with diminished capacity to maintain Omp25 solubility (Figure S2E). This was rescued in a dose-dependent manner by UBQLN1. The major cytosolic chaperones of the Hsp70 and Hsp90 family are not depleted by phenyl-sepharose. Therefore, whereas these general chaperones appear to maintain translocation competence of less hydrophobic protein precursors (Young et al., 2003), hydrophobic TMD-containing proteins require other factor(s) to prevent their aggregation. UBQLNs appear to be a highly conserved, widely expressed, and abundant family of factors that are capable of fulfilling this TMD-chaperoning function in vitro.

UBQLN Insufficiency Causes Aggregation of Membrane Protein Precursors

To understand the physiologic importance of this biochemical activity, we simultaneously disrupted UBQLN1, 2, and 4 in cultured cells using CRISPR/Cas9 methodology (Figure S3A) and analyzed the effect on transfected mitochondrial membrane proteins. We focused on ATP5G1 because its cytosolic precursor and mitochondrial mature product can be distinguished by import-dependent removal of its MTS (Figure S2C). Pulse-labeling of wild-type (WT) cells transfected with ATP5G1-HA showed both precursor and mature products. After a 1 hr chase, only the mature product persisted (Figure 2A). The lower levels of mature product after chase is likely due to degradation within mitochondria after failing to incorporate into pre-existing ATP synthase (Koppen and Langer, 2007). When mitochondrial import is inhibited with CCCP and valinomycin, only ATP5G1-HA precursor is observed after the pulse, and is completely degraded during the chase.

In UBQLN1/2/4 triple knockout (TKO) cells, we observed essentially identical amounts of mature ATP5G1-HA compared to WT cells (Figure 2A). In contrast, the amount of precursor was substantially (~3-fold) higher after the pulse and was incompletely degraded during the chase. Similar precursor stabilization preferentially in TKO cells was seen in the presence of CCCP and valinomycin (Figure 2A) and at a wide range of ATP5G1-HA expression levels (Figures S3B and S3C). This suggested that the absence of UBQLNs has little effect on ATP5G1-HA import, but significantly impairs the degradation of non-imported precursors. Indeed, immunoblotting of total cell lysate showed similar steady-state levels of mature ATP5G1-HA in WT and TKO cells, but increased precursor selectively in the latter (Figure 2A).

Finer pulse-chase studies under conditions of blocked mitochondrial import (Figure 2B) showed that degradation of non-imported precursor in WT cells was rapid and nearly complete within 20 min. In contrast, ~50% precursor remained in TKO cells after 40 min, and this stabilization was rescued by re-expressing physiologic levels of Myc-UBQLN1 (hereafter termed rescue cells; Figure S3A). In all cases, degradation was completely blocked by proteasome inhibition. Pulse-chase studies without and with proteasome inhibition in WT cells indicate that ~25% of nascent ATP5G1-HA molecules fail import (Figure S3D). Although this is exaggerated by overexpression (e.g., Figure S3C), import failure would always occur at some basal level, necessitating the UBQLN-dependent degradation pathway.

A role for UBQLNs in precursor degradation in cells was further supported by an interaction between newly synthesized ATP5G1-HA and Myc-UBQLN1 in rescue cells by co-immunoprecipitation (Figure 2C). This interaction was detergent sensitive, permitting selective release of ATPG1-HA from immunoprecipitated Myc-UBQLN1. Numerous nascent endogenous interacting partners (observed as a heterogeneous collection of products of varying sizes) were also eluted from Myc-UBQLN1 under these conditions, indicating their binding via primarily

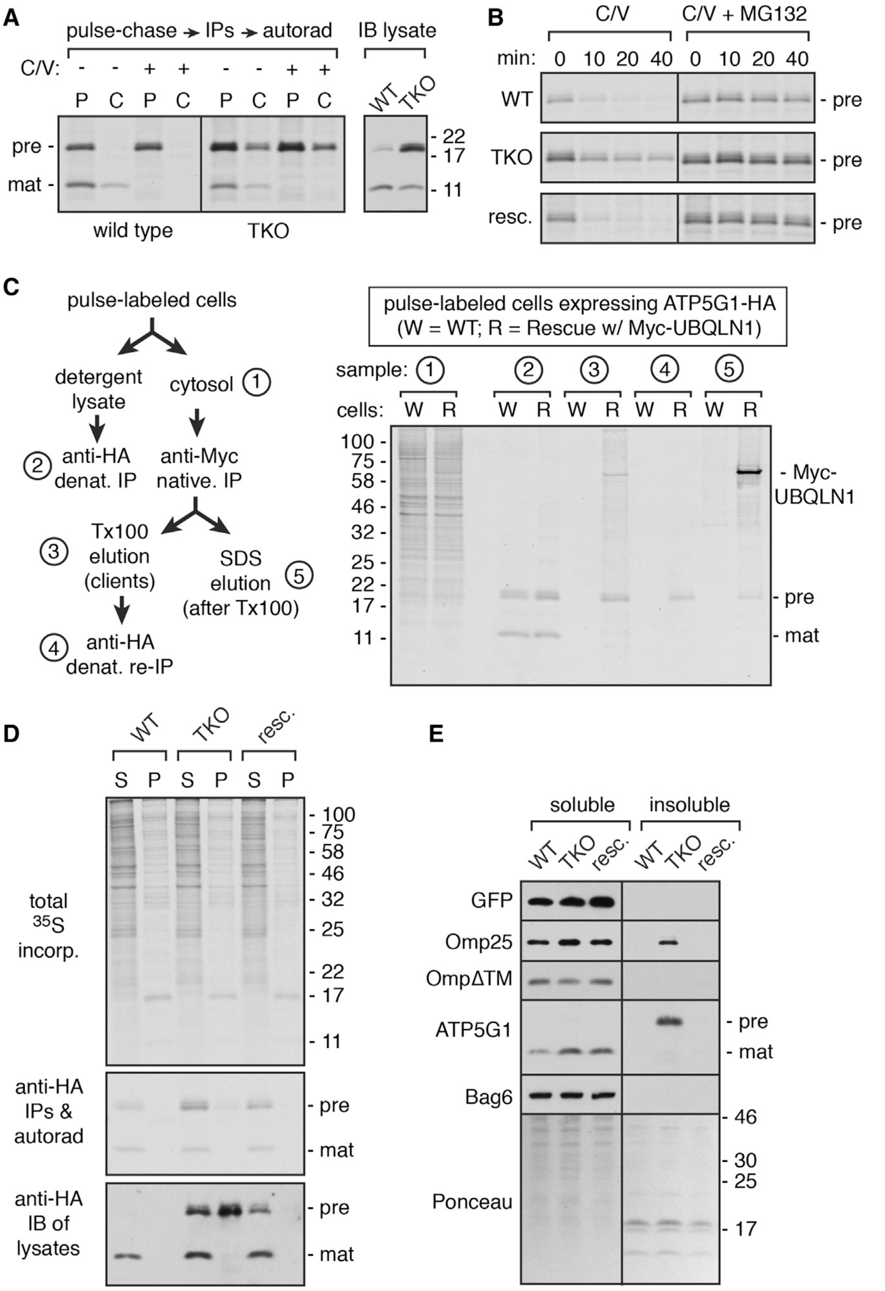

Figure 2. The Consequences of UBQLN Deficiency in Cells

(A) Wild-type (WT) or UBQLN triple knockout (TKO) cells expressing ATP5G1-HA were pulse-labeled with ^{35}S-methionine for 30 min and chased for 1 hr with unlabeled methionine. Immunoprecipitated ATP5G1-HA was detected using autoradiography. Where indicated, a mixture of CCCP and valinomycin (C/V) was included during the pulse-chase to inhibit mitochondrial import. The right image shows an anti-HA immunoblot of untreated total cell lysate to detect steady-state levels of ATP5G1-HA. The positions of precursor (pre) and mature (mat) forms of ATP5G1 are indicated.

(B) WT, TKO, and Myc-UBQLN1 rescue cells (resc.) were transfected with ATP5G1-HA and analyzed by pulse-chase labeling in the presence of C/V. Pulse time was 5 min, and chase times were from 0 to 40 min. Where indicated, the proteasome was inhibited with MG132.

(C) Detergent-free cytosolic lysates from cells pulse-labeled for 30 min with ^{35}S-methionine were subjected to the separation protocol shown on the left. The indicated fractions were analyzed by SDS-PAGE and autoradiography. The positions of Myc-UBQLN1 (only expressed in the rescue cells) and the precursor and mature forms of ATP5G1-HA (expressed in both cells) are indicated.

(D) WT, TKO, and rescue cells transfected with ATP5G1-HA were pulse-labeled for 30 min with ^{35}S-methionine, separated into detergent soluble (S) and insoluble (P) fractions, and either analyzed directly by autoradiography (top), immunoblotting with anti-HA (bottom), or immunoprecipitation with anti-HA and autoradiography (middle).

(E) WT, TKO, and rescue cells were transfected with plasmids expressing GFP, GFP-Omp25, GFP-Omp25ΔTM, or ATP5G1-HA, separated into detergent-soluble and insoluble fractions, and equivalent amounts of each fraction immunoblotted for the respective antigens. Endogenous Bag6 and the Ponceau-stained blot from one of the experiments are shown to illustrate uniformity of fractionation. The fractions for any given sample were analyzed on the same gel, and the image was assembled from the same exposure in each case.

See also Figures S3 and S4.

hydrophobic interactions. This is further consistent with our conclusion, based on in vitro analysis, that UBQLNs engage clients via TMDs.

At steady state, ATP5G1-HA precursors that fail to be degraded in TKO cells were mostly detergent insoluble (>70%), consistent with their aggregation (Figure 2D, bottom image). However, this aggregation was minimal (<10%) for newly synthesized ATP5G1-HA detected using pulse labeling (Figure 2D, middle image). This suggests that other chaperones, such as perhaps SGTA (e.g., Figure 1D), can temporarily maintain precursor solubility, but that the inability to degrade this product leads to its aggregation over time. Similar to ATP5G1, we observed increased detergent-insoluble Omp25 selectively in TKO cells (Figure 2E), without any appreciable difference in the amount of Omp25 successfully targeted to mitochondria (Figure S3E). As expected from in vitro interaction results (Figure 1B), UBQLN-dependent changes in Omp25 solubility were TMD dependent (Figure 2E).

Thus, for both a TA protein (Omp25) and a TOM/TIM pathway client (ATP5G1), UBQLNs are crucial for preventing aggregation and promoting efficient degradation of non-imported mitochondrial products. At this level of resolution, we have not detected a consistent effect on insertion, and do not observe any obvious alterations in mitochondrial morphology (Figure S3E), induction

of mitochondrial stress (Figure S4A), or reduced levels of respiratory chain complexes (Figure S4A) in TKO cells. This suggests that other TMD-binding partners (e.g., Figure 1A), perhaps aided by an activated heat shock response (Figures S4A and S4B), prevent aggregation over short time frames to permit membrane targeting and maintain mitochondrial biogenesis. In contrast, cytosolic precursor stabilization and aggregation, a constitutive heat shock response, and decreased fitness in TKO cells (Figures S4C and S4D) all point to the physiologic importance of UBQLNs in maintaining cytosolic protein homeostasis.

Pathologic UBQLN Depletion by Polyglutamine Aggregates

Earlier studies had suggested that aggregates of expanded polyglutamine (polyQ)-containing proteins, implicated in a variety of human diseases, may partially sequester UBQLNs (Doi et al., 2004; Wang and Monteiro, 2007; Mori et al., 2012; Rutherford et al., 2013). This raised the possibility that polyQ aggregates might affect cytosolic protein homeostasis by depleting UBQLNs. We tested this idea by developing a single-cell fluorescent reporter of UBQLN-mediated degradation and analyzing whether this reporter was preferentially stabilized in polyQ aggregate-containing cells.

To design a constitutive UBQLN pathway client, we deleted the MTS from GFP-tagged ATP5G1 (GFP-ATP5G1ΔMTS) to force its translocation failure. An in-frame fusion with RFP separated by a viral 2A sequence (de Felipe et al., 2006) provided an internal expression control in which an RFP molecule is produced as a separate product every time a GFP-ATP5G1ΔMTS is translated (Figure 3A). Thus, the GFP:RFP ratio provides a quantitative indicator of the stability of the GFP-tagged protein relative to the long-lived RFP.

Relative to GFP-2A-RFP, most cells expressing GFP-ATP5G1ΔMTS-2A-RFP showed an ~10- to 100-fold lower GFP:RFP ratio (Figure 3B). This ratio increased in TKO cells, and was partially reversed in rescue cells. The GFP-ATP5G1ΔMTS that was stabilized in TKO cells was primarily detergent insoluble (Figure S5A), and appeared to be in amorphous aggregates by microscopy (Figure S5B). The GFP:RFP ratio for the control construct did not change in TKO or rescue cells (Figure 3B), validating the specificity of the ATP5G1ΔMTS reporter.

As expected, CFP-tagged Huntingtin exon 1 containing 105 glutamines (HttQ105) formed aggregates in ~25%–30% of transfected cells, whereas CFP-HttQ25 remained diffusely nucleocytoplasmic (Figures S5C and S5D). When CFP-HttQ105 was expressed in rescue cells, Myc-UBQLN1 was observed to co-associate with the HttQ105 aggregate, with corresponding depletion from the bulk cytosol in many (but not all) aggregate-containing cells (Figure 3C). Biochemical fractionation of these aggregate-containing cells verified that Myc-UBQLN1 was primarily in the insoluble pellet (along with HttQ105), with more than 50% depletion from the supernatant (Figure S5E). Myc-UBQLN1 was predominantly soluble in HttQ105 cells lacking aggregates, HttQ25-expressing cells, and non-transfected cells. Very similar results were observed for endogenous UBQLN2 in WT cells (Figure 3D). Thus, polyQ aggregates efficiently engage UBQLNs to markedly deplete their availability in the bulk cytosol.

Using the GFP:RFP ratiometric assay, we found that GFP-ATP5G1ΔMTS was preferentially stabilized in cells containing HttQ105 aggregates, but not HttQ25-expressing or aggregate-lacking HttQ105 cells (Figure 3E). The effect was a partial phenocopy of TKO cells (compare to Figure 3B), consistent with partial UBQLN depletion by HttQ105 aggregates. Importantly, the stabilized GFP-ATP5G1ΔMTS only partially co-localized with polyQ, as judged by fluorescence microscopy (Figure 3F) and flow cytometry (Figure S5F), arguing against co-aggregation as the sole explanation for its stabilization. Hence, UBQLN depletion by pathologic sequestration in polyQ aggregates contributes to impaired degradation of membrane protein precursors that fail successful mitochondrial insertion.

UBQLN1 Recruits an E3 Ligase to Facilitate Client Ubiquitination

To understand the mechanistic basis of UBQLN-mediated degradation, we turned to biochemical studies. We observed that PURE system-produced Omp25-UBQLN1 complex added to cytosol resulted in Omp25 ubiquitination (Figure S6A). This is similar to Omp25 ubiquitination observed in complex with Bag6, a chaperone known to recruit an E3 ligase (Hessa et al., 2011; Rodrigo-Brenni et al., 2014). The specificity of ubiquitination was verified by the markedly lower levels seen for complexes with the targeting factor TRC40 or UBQLN1ΔUBA. These results suggested that UBQLN1 may facilitate client ubiquitination by recruiting an E3 ligase, possibly via its UBA domain.

To test this idea, we determined if an E3 ligase activity could be co-purified with Omp25-UBQLN1 complexes (Figure 4A). RRL was fractionated to remove free ubiquitin and UbcH5 (Hessa et al., 2011), and this ubiquitination-deficient lysate was used to synthesize Omp25 in the presence of FLAG-tagged UBQLN1. The Omp25-UBQLN1 complex was affinity purified via the FLAG tag and incubated with E1, E2, ATP, and His-tagged ubiquitin. Pulldowns via the His tag revealed that Omp25 was ubiquitinated in an E1/E2-dependent manner, indicating that the purified complexes contained an E3 ligase (Figure 4B).

Ubiquitination was not observed when UBQLN1ΔUBA was used, but was enhanced with UBQLN1ΔUBL. Deletion of the UBL domain presumably enhances ligase recruitment by freeing the UBA domain, with which the UBL domain can associate (Lowe et al., 2006; Zhang et al., 2008). Consistent with a key role for the UBA domain in ligase recruitment, we found that ubiquitination of Omp25 translated in RRL is dominantly inhibited most potently by excess UBQLN1ΔUBA (Figure 4C). In contrast, a TMD mutant (Omp25-3R) that cannot interact with UBQLNs (Figure 1B) is not inhibited in its ubiquitination by UBQLN1ΔUBA (Figure 4C). Similar results were observed for a different mitochondrial TA protein (Figure S6B). Thus, we conclude that UBQLN1 can recruit an E3 ligase in a UBA domain-dependent manner to mediate ubiquitination of its bound client.

UBQLN1 Clients Are Insertion Competent Prior to Their Ubiquitination

During the course of our experiments, we observed that Omp25 targeting to mitochondria and ATP5G1 import were preferentially reduced for molecules that had acquired one or more ubiquitins

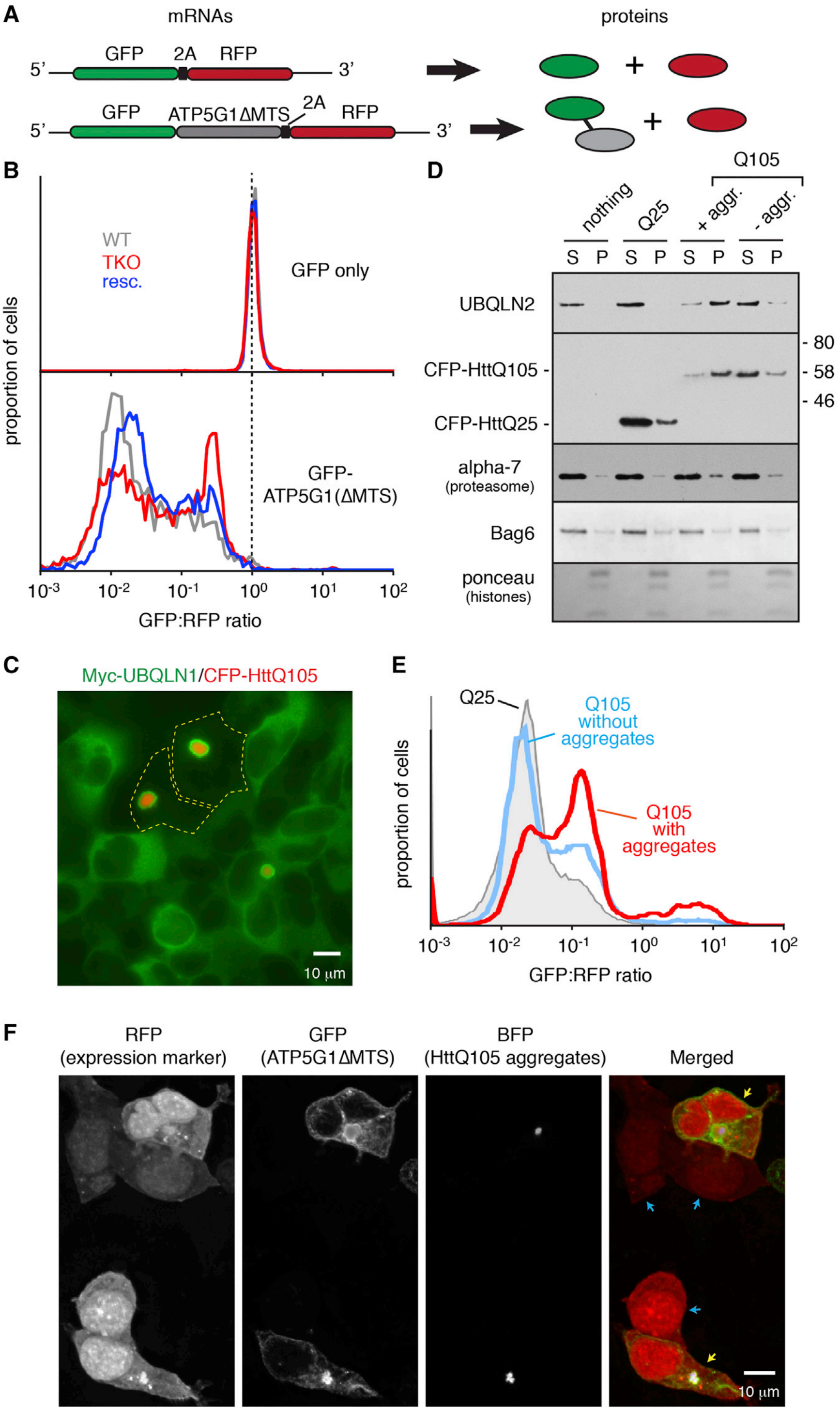

Figure 3. Pathologic UBQLN Depletion by PolyQ Aggregates

(A) Schematic of the expected mRNA and protein products of GFP-2A-RFP constructs.

(B) Flow cytometry analysis of the GFP:RFP ratio of the indicated cell lines expressing GFP-2A-RFP (top) or GFP-ATP5G1ΔMTS-2A-RFP (bottom).

(C) TKO cells rescued with Myc-UBQLN1 were transfected with CFP-HttQ105 and the localization analyzed by immunostaining (Myc-UBQLN1, green) or fluorescence (CFP-HttQ105, red). Yellow dashed lines are cell margins.

(D) HEK293 cells were transfected with CFP-HttQ25 or CFP-HttQ105, with the latter separated by FACS into cells containing or lacking aggregates (see Figure S5D). The cells were separated into soluble (S) and insoluble (P) fractions, and analyzed by immunoblotting. The Bag6 blot and Ponceau-stained membrane (to detect the nuclear histones) illustrate uniform fractionation and loading.

(E) HEK293 cells co-expressing CFP-HttQ105 and GFP-ATP5G1ΔMTS-2A-RFP were analyzed by flow cytometry. Histograms of the GFP:RFP ratio of the ATP5G1 reporter are shown for aggregate-containing (red) and -lacking (blue) cells. The profile for cells expressing CFP-HttQ25 is shown in gray for comparison.

(F) Deconvoluted projection image of cells co-transfected with BFP-HttQ105 and GFP-ATP5G1ΔMTS-2A-RFP. Cells containing and lacking HttQ105 aggregates are indicated by yellow and blue arrows, respectively, in the merge image.

See also Figure S5.

(Figures 5A, 5B, and S7A). This result implies that there exists mechanism(s) to avoid targeting and import of ubiquitinated proteins. We therefore investigated the relationships between engagement of UBQLNs, membrane targeting, and ubiquitination.

To determine if engagement of UBQLN1 is a commitment to degradation, we tested the mitochondrial targeting competence of the Omp25-UBQLN1 complex assembled using the PURE system. Omp25 showed equally high and specific mitochondrial targeting when complexed with either UBQLN1 or SGTA, an unrelated TMD-binding chaperone (Figure 5C). The efficiency of insertion was comparable to that seen for Omp25 produced in RRL. This indicates that engagement of UBQLN1 is not mutually exclusive with insertion, and is not necessarily a commitment to degradation. As expected, Omp25 synthesized in the PURE system in the absence of any chaperone showed no capacity for specific membrane targeting. Thus, precluding aggregation of nascent Omp25 by either UBQLN1 or SGTA is sufficient to maintain insertion competence and permit mitochondrial targeting.

To test whether ubiquitination is the key step for switching from insertion competence to degradation, we generated a "constitutively" ubiquitinated Omp25 in which the deubiquitinase-resistant ubiquitin(G76V) was fused to the N terminus of Omp25 (Ub-Omp25). Translation of Ub-Omp25 in RRL

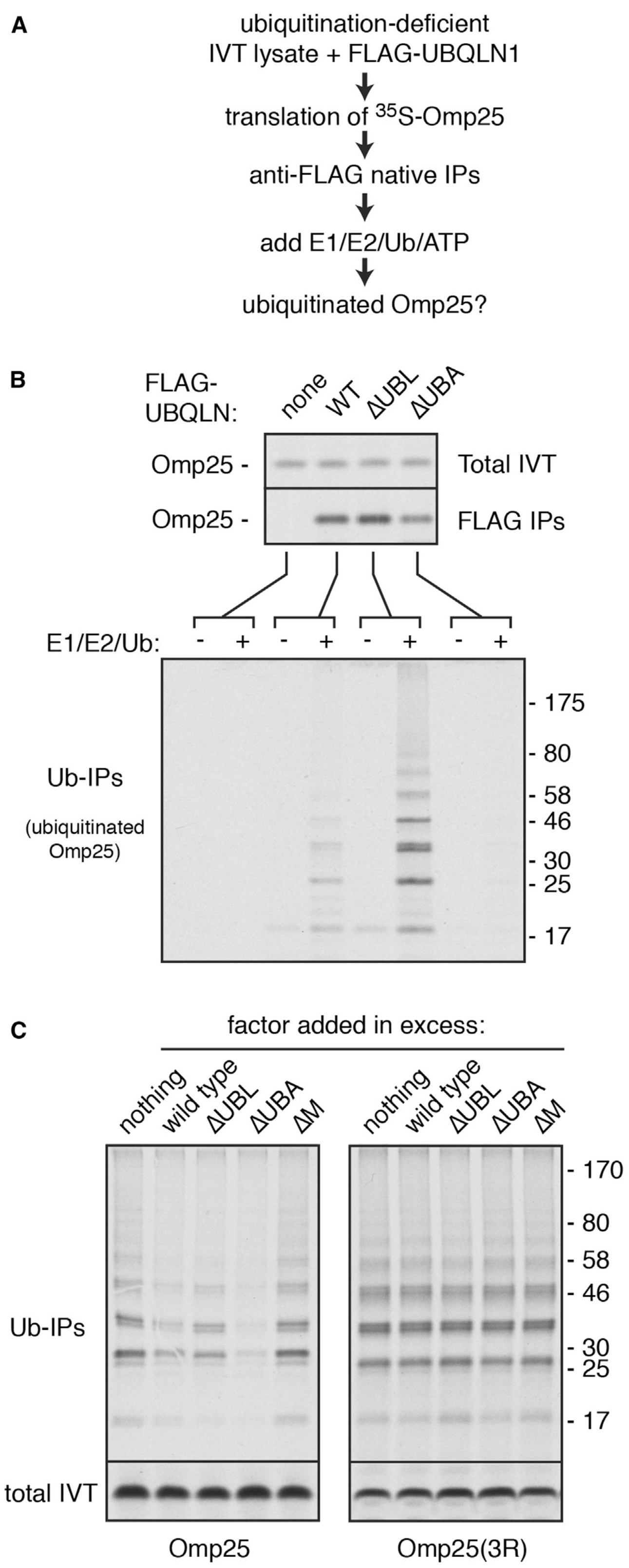

Figure 4. UBQLN1 Recruits an E3 Ligase for Client Ubiquitination

(A) Experimental strategy to detect E3 ligase activity associated with UBQLN1-Omp25 complexes.

(B) A construct containing the Omp25 TMD was translated in RRL depleted of ubiquitin and the UbcH5 E2 enzyme but supplemented with the indicated FLAG-tagged UBQLN1 protein and ^{35}S-methionine. After translation, anti-FLAG affinity resin was used to isolate the UBQLN1-Omp25 complexes, the sample divided in two, and one half supplemented with a mixture of E1, E2, His-tagged Ubiquitin, and ATP. The reaction products were then affinity purified via the His tag on Ubiquitin, and the ^{35}S-Omp25 was detected using autoradiography. Aliquots of the sample at each step are shown.

(C) The UBQLN-associating client Omp25 and the non-associating Omp25(3R) mutant were translated in RRL containing ^{35}S-methionine, the indicated UBQLN1 protein (at 1 μM), and His-tagged Ubiquitin (at 10 μM). Shown are aliquots of the total translation products and the ubiquitinated products isolated via His-tagged Ubiquitin.

See also Figure S6.

supplemented with excess UBQLN1 (to outcompete capture by other chaperones) resulted in a complex that was sharply reduced in mitochondrial insertion (Figure 5D). This inhibitory effect was not observed with UBQLN1ΔUBA, suggesting that the interaction between ubiquitin on the client with the UBA domain of UBQLN1 may be important for terminating insertion competence.

This was demonstrated by showing that Ub-Omp25 regained insertion competence when ubiquitin contained the I44A mutation to inhibit its association with the UBA domain (Figures 5E and 5F). Similarly, the F559A mutation in the UBA domain, which disrupts ubiquitin binding (Zhang et al., 2008), also restored insertion competence to Ub-Omp25 (Figure 5F). Thus, the Omp25-UBQLN1 complex retains insertion competence until Omp25 acquires ubiquitin, whose inhibitory effect on insertion depends on interaction with the UBA domain of UBQLN1. Similar results were observed for ATP5G1 (Figure S7B).

Mechanism of Fate-Switching by UBL-UBA-Ubiquitin Interactions

Having identified the key step when insertion competence is lost, we could investigate the mechanistic basis for this shift and determine how it might facilitate degradation. A pre-requisite for membrane insertion is release of the TMD from its protective chaperone. We therefore suspected that ubiquitination of a UBQLN1-bound client precludes insertion by preventing efficient release of the TMD. To test this idea, we modified the PURE system to re-assign the amber stop codon (UAG) to code for *p*-benzoyl-L-phenylalanine (BpF), a UV-activated photo-crosslinker (Chin et al., 2002). We used this system to produce UBQLN1-Omp25 complexes containing BpF in the TMD. Control experiments verified the production of UV- and BpF-dependent photo-adducts to UBQLN1, providing a means to monitor the Omp25-UBQLN1 interaction (data not shown). Importantly, the photo-crosslinking reaction can proceed on flash-frozen samples (e.g., Shao and Hegde, 2011), providing a snapshot of TMD interactions at any particular moment.

Using this assay, we could determine whether client release from UBQLN1 is impeded by a ubiquitin on the client (Figure 6A). To prevent re-binding of client released from UBQLN1, we used calmodulin (CaM), a protein capable of efficient and stable binding to hydrophobic domains in the presence of high Ca^{2+} (Shao and Hegde, 2011), as a “sink” to capture exposed TMDs. Kinetics of Omp25 release showed a $t_{1/2}$ of ~1–2 min (Figure 6B),

CellPress

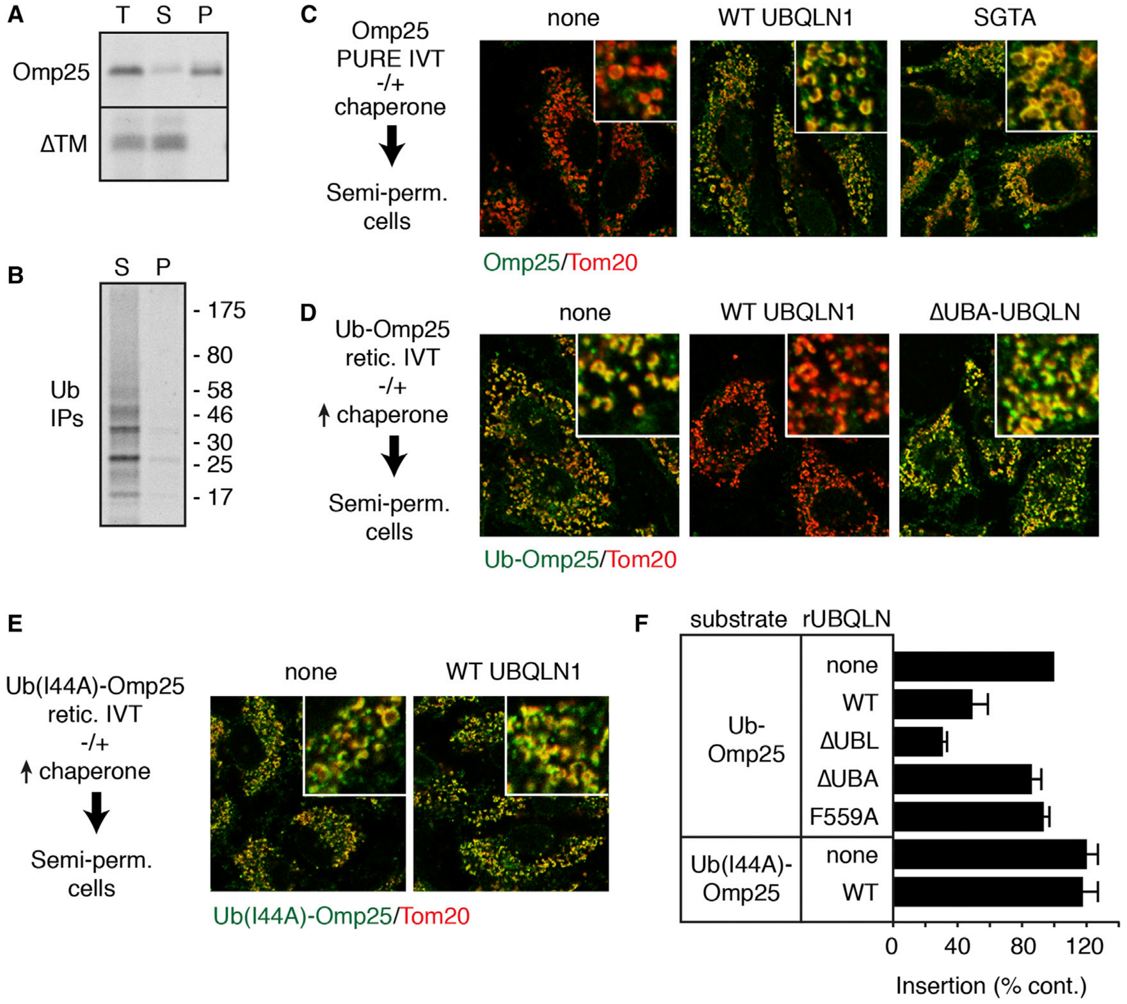

Figure 5. UBQLN1 Conditionally Inhibits Mitochondrial Insertion of Ubiquitinated Clients

(A) Constructs containing the Omp25 TMD or a mutant disrupted in its hydrophobic domain (ΔTM) were translated in RRL in the presence of ^{35}S-methionine and incubated with semi-permeabilized cultured cells. The total reactions (T) were fractionated into a soluble supernatant (S) and cell pellet (P), and the translated products were detected using autoradiography.

(B) A construct containing the Omp25 TMD was translated in a reaction containing ^{35}S-methionine, His-tagged Ubiquitin, and semi-permeabilized cells. After translation, the reaction was fractionated as in (A), the ubiquitinated products in each fraction isolated via the His tag, and the ubiquitinated Omp25 detected using autoradiography.

(C) An HA-tagged construct containing the Omp25 TMD was translated in the PURE system supplemented without or with the indicated chaperone (either UBQLN1 or SGTA), and the products applied to semi-permeabilized cells. After washing, immunofluorescence microscopy was used to detect the localization of the translation product (green) relative to mitochondria (red).

(D) An HA-ubiquitin-tagged construct containing the Omp25 TMD was translated in RRL containing 1 μM of the indicated UBQLN1 protein. The translation products were applied to semi-permeabilized cells and visualized as in (C).

(E) As in (D), but with a construct containing the I44A mutation in Ubiquitin.

(F) The indicated substrates were translated in RRL supplemented with the indicated recombinant UBQLN1 proteins and analyzed for insertion into the mitochondria of semi-permeabilized cells as in (A). The graph shows the normalized quantification from three experiments (mean ± SD).

See also Figure S7.

which is suitably fast to permit multiple opportunities for mitochondrial insertion before client ubiquitination. In contrast to Omp25, very little Ub-Omp25 was released from UBQLN1 in 15 min, whereas ~80% of Ub(I44A)-Omp25 was released (Figure 6C). Thus, the UBQLN1-Omp25 complex dynamically dissociates and re-associates rapidly, whereas Ub-Omp25 is stably bound to UBQLN1 over physiologically relevant time frames.

In addition to precluding insertion, degradation should ideally be favored for an ubiquitinated UBQLN1 client. An important clue for how this occurs came from the comparative analysis of interaction partners for UBQLN1ΔUBA versus UBQLN1ΔUBL (Figure 7A): the former preferentially associated with the proteasome, while the latter did not. This suggested a model where engagement of ubiquitin on the client by

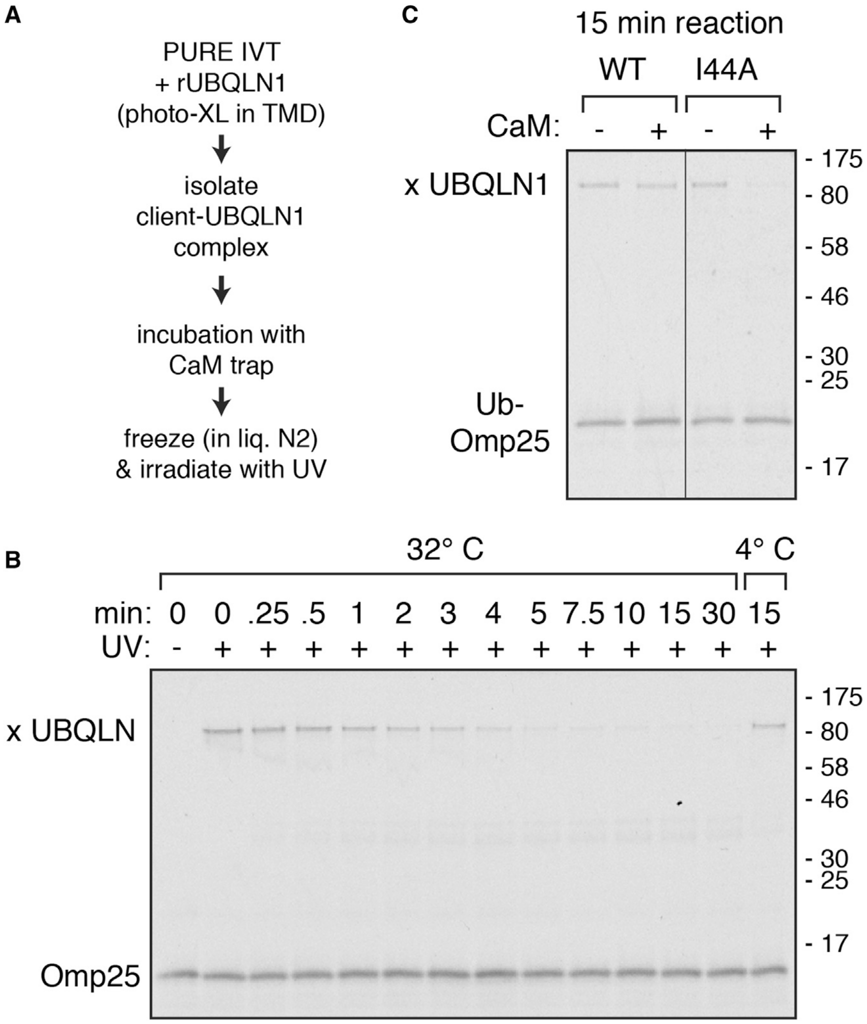

Figure 6. Client Ubiquitination Alters the Dynamics of UBQLN1 Interaction

(A) Experimental strategy to detect Omp25 dissociation from UBQLN1.

(B) A construct containing an amber codon in the Omp25 TMD was translated in a modified PURE system containing BpF-charged amber suppressor tRNA and recombinant UBQLN1. The UBQLN1-Omp25 complexes were isolated via sucrose gradient, mixed with 10-fold excess calmodulin (CaM), and incubated for the indicated times at either 32°C or 4°C. Aliquots were flash-frozen, irradiated with UV light in the frozen state, and analyzed by SDS-PAGE. Omp25 and its photo-adduct with UBQLN1 are indicated. Crosslinks to CaM are not clearly observed due to heterogeneity and lower efficiency of adduct formation, but can be seen faintly in the middle part of the gel.

(C) Ub-Omp25 containing a photo-probe in the TMD, or a variant containing the I44A mutation in Ubiquitin, was assembled into a UBQLN1 complex as in (B). The sample was mixed with CaM, incubated for 15 min at 32°C, subjected to UV irradiation, and analyzed by SDS-PAGE and autoradiography. Ub-Omp25 and its photo-adduct with UBQLN1 are indicated.

UBQLN's UBA domain frees the UBL domain to engage the proteasome.

To test this idea (Figure 7B), we first modified RRL by depleting all of its TMD-binding proteins (by passing over phenyl-sepharose) and removing free ubiquitin (by fractionation using anion-exchange). This translation system was then replenished with recombinant FLAG-tagged UBQLN1 variants at endogenous levels. This allows UBQLN1 to be the only TMD-binding protein for Omp25, whose ubiquitination status could be controlled by using Ub-Omp25. Following translation of a client in this system at levels sufficient to saturate the recombinant UBQLN1, the FLAG tag was used to capture UBQLN1 and its targeting to the proteasome was judged by immunoblotting.

Whereas UBQLN1ΔUBA constitutively associated with the proteasome, and UBQLN1ΔUBL failed to associate, WT UBQLN1 associated in a client-modulated manner (Figure 7C). UBQLN1 interaction with the proteasome was ~2- to 3-fold higher when UBQLN1 was loaded with Ub-Omp25 relative to Omp25. This difference is similar to that observed between UBQLN1ΔUBA and WT UBQLN1, indicating that UBA domain sequestration by client ubiquitin was involved. Thus, UBQLN1 favors proteasome association when its client is ubiquitinated, and this depends on its UBL domain. The shift toward proteasome targeting accompanies a simultaneous inhibition of insertion capacity (Figure 5), with both events relying critically on the UBA interaction with client ubiquitin. Ubiquitin is therefore used by UBQLN1 to regulate fate-switching of the bound client.

DISCUSSION

In this study, we have discovered that UBQLNs are a new class of chaperones dedicated to shielding TMDs from aggregation during their transient residence in the cytosol. UBQLN specificity, while overlapping with other TMD chaperones, is tuned to favor mitochondrial membrane proteins due to their slightly lower overall hydrophobicity. Mechanistic analysis of UBQLN1 function indicates that beyond preventing client aggregation, it acts at the crossroads between mitochondrial membrane protein biogenesis and degradation. Hence, a key function of UBQLNs is to facilitate degradation of mitochondrial membrane proteins that fail successful insertion, a pathway likely to be of crucial importance to normal cellular physiology and various pathologic conditions.

The molecular basis of triage between biosynthesis and degradation by UBQLNs appears to be a multi-step process involving a sequence of dynamic and non-dynamic inter- and

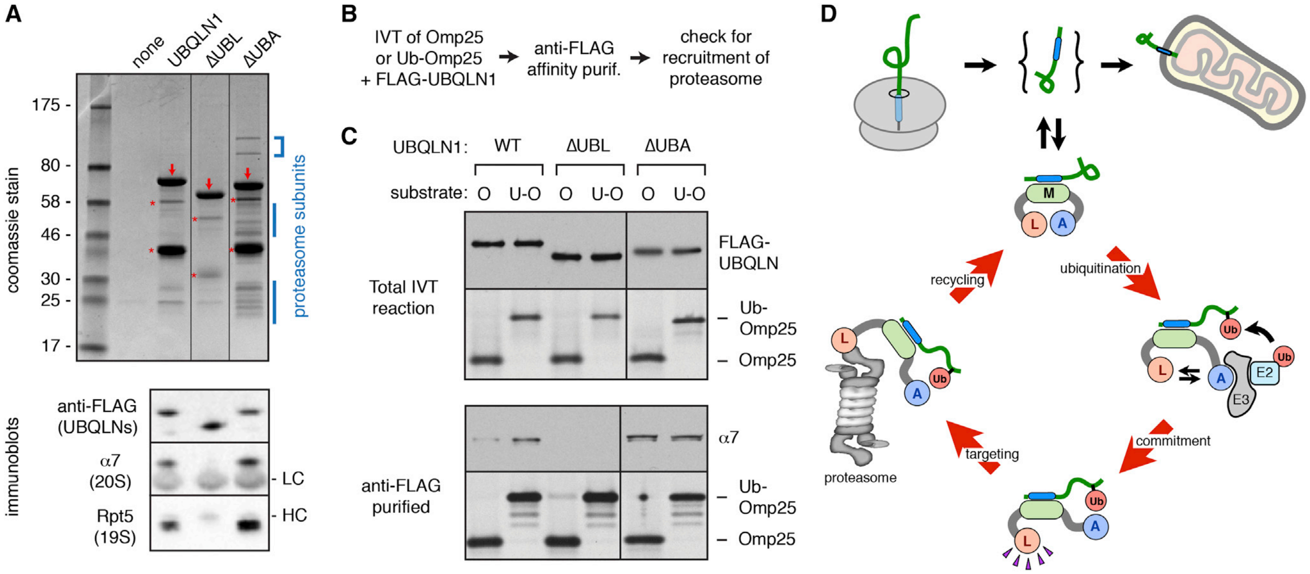

Figure 7. Client-Regulated Proteasome Targeting by UBQLN1

(A) Recombinant FLAG-tagged UBQLN1 or the indicated mutant was added to total RRL, incubated, recovered via anti-FLAG affinity resin, and analyzed by SDS-PAGE. The positions of the recombinant proteins (downward red arrows) and proteolytic fragments (red asterisks) are shown. Blue marks indicate proteasome subunits. Bottom: a similar analytic scale experiment analyzed by immunoblotting for the UBQLN1 protein and proteasome subunits.

(B) Experimental strategy for analyzing proteasome targeting of UBQLN1-Omp25 complexes.

(C) Constructs containing the Omp25 TMD without or with an in-line fused ubiquitin were translated in lysates lacking ubiquitin, UbcH5, and the major TMD-binding factors, but supplemented with the indicated recombinant UBQLN1 at 40 nM. After translation, UBQLN1-Omp25 complexes were affinity purified via the FLAG tag on UBQLN1, and the products analyzed by autoradiography (to detect the Omp25 substrate) or immunoblotting for the proteasome subunit α7. An aliquot of the starting translation reaction was analyzed in parallel (top).

(D) Model for UBQLN function. TMD-containing proteins destined for the mitochondria can be intercepted by UBQLNs during their cytosolic transit. This binding is dynamic, permitting opportunities for mitochondrial targeting in the unbound state. Over time, the UBA domain (blue) facilitates recruitment of an E3 ligase to mediate client ubiquitination. This results in a conformational change that simultaneously increases client-UBQLN affinity (via UBA-ubiquitin interactions), while exposing the UBL domain (light orange). This re-structured complex is now favored for proteasome targeting.

intramolecular interactions (Figure 7D). Engagement of UBQLN by a membrane protein is highly dynamic, and does not commit the client for degradation. This suggests that UBQLNs, whose total concentration in cytosol we estimate to be ~1–2 μM, act as surveillance factors for exposed TMDs. Dynamic engagement by a relatively abundant factor not only protects against aggregation in the bound state, but also allows opportunities for engaging the yet unidentified targeting pathway to mitochondria in the unbound state.

This opportunity for mitochondrial targeting is time-limited by the contingency that, while associated with UBQLN, an E3 ligase can be recruited to ubiquitinate the client. The timing of ubiquitination is presumably determined by a combination of how long the ligase is bound to UBQLN and the kinetics of ligase activity. Once ubiquitinated, the previously dynamic client-UBQLN interaction changes to a comparatively stable interaction that precludes additional insertion attempts. Key to this stabilization is the UBA-ubiquitin interaction, whose K_d of ~8 μM is 10-fold lower than the ~80 μM K_d for the UBA-UBL interaction (Lowe et al., 2006). Thus, the UBA domain exploits the high local concentration of the client's ubiquitin and the avidity gained by the M domain-TMD interaction to effectively switch the fate of membrane protein clients away from insertion. Ubiquitin binding to the UBA domain might also protect the client from deubiquitinases to limit the reversibility of this switch in fate.

The higher affinity interaction of the UBA domain with client ubiquitin relative to the UBL domain means the latter becomes preferentially freed only after the client is ubiquitinated. Hence, proteasome targeting via the UBL domain is favored for UBQLN containing a ubiquitinated client, explaining how futile excursions to the proteasome are minimized for UBQLN that is either empty or loaded with non-ubiquitinated client. Our tools to prepare defined UBQLN-client complexes capable of efficient proteasome targeting now open up opportunities to mechanistically dissect both the targeting reaction and the transfer of clients from UBQLN to the proteasome.

Based primarily on similarity to Rad23 domain organization (Su and Lau, 2009), earlier work had suggested that yeast Dsk2 (and by inference, the homologous UBQLNs) act as putative "shuttling factors" that deliver polyubiquitinated clients to the proteasome (Ghaboosi and Deshaies, 2007). Curiously, UBQLNs contain only one UBA domain relative to Rad23's tandem UBAs, and hence UBQLNs may not have linkage-specificity for polyubiquitin chains (Zhang et al., 2008). In addition, the methionine-richness of the M domain is conserved from yeast to humans, but is not seen in Rad23. Our findings provide an explanation for these differences and revise the biological role for UBQLNs. Rather than simply bridging ubiquitin with the proteasome, UBQLNs encode client specificity in their M domains and actively participate in deciding the client's fate via a combination

of E3 ligase interactions and dynamic client interactions. Thus, the single low-affinity UBA domain is not selecting clients, but rather stabilizing them after their selection via the M domain and ubiquitination by UBQLN-associating E3 ligases.

In this light, the generic shuttling function assumed for other UBL-UBA proteins merits re-evaluation. Given the ability of intrinsic ubiquitin receptors on the proteasome to select ubiquitinated clients (Finley, 2009), it is perhaps more attractive to view the UBL-UBA proteins as a family of client-specific factors for regulated degradation or quality control. In this view, the UBA-UBL proteins may mediate client ubiquitination analogous to our findings with UBQLNs, or preferentially associate with only certain ubiquitinated clients as dictated by non-UBA substrate-binding region(s). More specific roles for these proteins may better explain their distinct phenotypes and evolutionary conservation of regions, such as the M domain of UBQLNs, outside the UBL-UBA regions (Su and Lau, 2009).

The function of UBQLNs in degrading non-inserted mitochondrial membrane proteins is analogous to the role of Bag6 complex in degrading mislocalized proteins destined for the ER (Hessa et al., 2011). Indeed, UBQLNs and Bag6 have overlapping client specificities, and both are capable of also impacting the respective biosynthesis pathways. Consistent with this conclusion, UBQLN4 was recently observed to influence degradation of proteins that fail to successfully translocate into the ER (Suzuki and Kawahara, 2016), a set of clients also handled by the Bag6 pathway (Hessa et al., 2011). It seems likely that UBQLNs and Bag6 act in partially redundant pathways for the management of diverse hydrophobic domains that must necessarily transit the cytosol en route to either an intracellular membrane or the proteasome. We have observed that while many substrates may prefer one or the other factor in the cytosol, they are capable of binding the non-preferred factor when the primary factor is absent. Such a system would impart substantial robustness to these quality control systems by having alternate pathways for degradation. This would come at the expense of taxing these alternative pathways when one pathway is ablated, causing a general "proteostasis" insufficiency (and an accompanying stress response as seen in TKO cells) that indirectly impacts the fate of substrates for other pathways.

Our assignment of a molecular function for UBQLNs in mitochondrial precursor degradation has implications for both cellular physiology and disease. Mitochondrial import efficiency is not uniformly high and is modulated by physiologic and pathologic stimuli (Harbauer et al., 2014; Schmidt et al., 2011; Wright et al., 2001). It is therefore intriguing that conditional knockout mice lacking UBQLN1 in mouse brain display increased sensitivity to oxidative stress (Liu et al., 2014), during which import is probably impaired (Wright et al., 2001). Furthermore, UBQLNs are upregulated in response to oxidative stress (Ko et al., 2002), supporting a potential role in aiding recovery from the stress.

Conversely, conditions that deplete or impair UBQLNs would affect cytosolic proteostasis. Hence, the finding that UBQLNs are substantially depleted from the bulk cytosol of cells containing polyQ aggregates may help explain their impairment in degradation of a UBQLN client. As these clients would spill over into other quality control pathways, their increased demand could result in the more generalized effects on proteostasis observed in many studies (Labbadia and Morimoto, 2015). It is noteworthy that while a wide range of proteins have been found in aggregates (Olzscha et al., 2011), the precise level of depletion has rarely been defined. In the case of UBQLNs at endogenous levels, the extent of sequestration is clearly sufficient to deplete it from the cytosol. Thus, part of the phenotype caused by polyQ aggregates may result from UBQLN insufficiency, perhaps explaining why UBQLN1 overexpression can partially rescue a mouse model of polyQ disease (Safren et al., 2014). With our introduction of a range of cellular and biochemical assays for UBQLN function, it should now be possible to examine these ideas with greater precision, including the prospect of determining why certain mutations in UBQLN2 cause neurodegenerative disease in humans (Deng et al., 2011).

EXPERIMENTAL PROCEDURES

Plasmids, Antibodies, and Proteins

A list of plasmids is provided in Table S1. Plasmids were prepared by standard methods. Antibodies against the following proteins were purchased: UBQLN1/2 (Sigma, clone 5F5), UBQLN4 (Abcam ab106443), L9 and Tom20 (Santa Cruz Biotech., T-17 and FL-145), HA (Covance, clone 16B12), Rpt5 (Abcam ab22635), α7 (Enzo Life Sciences PW8110), FLAG (Sigma, clone M2), Hsc/Hsp70 (AssayDesigns, SPA-822), Hsp60 (Abcam ab46798), ClpP (Abcam ab124822), Actin-HRP (Sigma A3854), OxPhos cocktail (Abcam ab110411). FLAG-M2 Affinity resin was from Sigma, and GFP-trap from Chromotek. Antibodies to Bag6, TRC40, SGTA, TRAPα, GFP, and RFP have been described (Fons et al., 2003; Mariappan et al., 2010; Hessa et al., 2011). Anti-Myc was clone 9E10. Anti-HA used for IPs and blots (e.g., Figure 2A) was raised in rabbits against the KLH-HA peptide conjugate. Recombinant proteins were either purchased (His6-Ubiquitin, E1, and E2 from Boston Biochem) or expressed and purified from *E. coli* as described (Mateja et al., 2015).

Cell Culture

HeLa and Flp-in T-Rex HEK293 cells (Invitrogen) were cultured in DMEM supplemented with 10% fetal bovine serum and appropriate selection antibiotics. Gene disruption in HEK293 cells by CRISPR was performed as described (Ran et al., 2013) with guide RNA sequences provided in the Supplemental Experimental Procedures. Rescue cells were made by integrating a Myc-UBQLN1 expression cassette into the FRT site of TKO cells by Flp recombination (Invitrogen). 10 ng/ml doxycycline was used for induction of the integrated gene at the FRT site. Flow cytometry and FACS separation based on aggregates, pulse-chase experiments, fluorescence microscopy, and the detergent solubility assay have been described (Ramdzan et al., 2012; Ashok and Hegde, 2009).

In Vitro Biochemistry

In vitro translation (IVT) reactions in the RRL and PURE systems were as described (Sharma et al., 2010; Shimizu and Ueda, 2010). DEAE and/or phenyl-sepharose fractionation of RRL was performed as described elsewhere (Hessa et al., 2011; Shao and Hegde, 2011). Site-specific photo-crosslinker incorporation by amber suppression in the PURE system was performed as described elsewhere (Shimizu et al., 2001). Affinity purification of IVT translation products and mass spectrometry (Stefanovic and Hegde, 2007), ubiquitination analysis (Rodrigo-Brenni et al., 2014), photo-crosslinking (Shao and Hegde, 2011), targeting assays in semi-permeabilized cells (Setoguchi et al., 2006), sucrose gradient separations (Stefanovic and Hegde, 2007), and preparation of chaperone-client complexes in the PURE system (Mateja et al., 2015) have all been described.

SUPPLEMENTAL INFORMATION

Supplemental Information includes Supplemental Experimental Procedures, seven figures, and one table and can be found with this article online at http://dx.doi.org/10.1016/j.molcel.2016.05.020.

AUTHOR CONTRIBUTIONS

Conceptualization, R.S.H. and E.I.; Investigation, E.I., E.Z., S.S., M.L.W., and R.S.H.; Writing—Original Draft, R.S.H.; Writing—Review and Editing, E.I., E.Z., S.S., M.L.V., R.J.K., R.S.H.; Supervision, R.S.H. and R.J.K.

ACKNOWLEDGMENTS

We are grateful to J. James for advice on the use of 2A peptides, M. Barczyk for preparing anti-Myc antibody, M. Daly for help with flow cytometry, V. Romashova for help with cell sorting, M. Pasche for microscopy support, M. Skehel for mass spectrometry, M. Kivlen for preparing PURE system factors, J. Chin for amber suppression constructs, M. Rodrigo-Brenni for advice on ubiquitination assays, K. Yanagitani for constructs, and R.S.H. lab members for useful discussions. This work was supported by the UK Medical Research Council (MC_UP_A022_1007 to R.S.H.). E.I. was supported by a fellowship from the Japan Society for the Promotion of Science, an MRC Career Development Fellowship, and a Grant-in-Aid for Young Scientists. E.Z. was supported by an MRC Postdoctoral Fellowship. S.S. was supported by an MRC Career Development Fellowship and a St. John's College Title A fellowship.

Received: January 12, 2016
Revised: April 4, 2016
Accepted: May 16, 2016
Published: June 23, 2016

REFERENCES

Ashok, A., and Hegde, R.S. (2009). Selective processing and metabolism of disease-causing mutant prion proteins. PLoS Pathog. *5*, e1000479.

Bernstein, H.D., Poritz, M.A., Strub, K., Hoben, P.J., Brenner, S., and Walter, P. (1989). Model for signal sequence recognition from amino-acid sequence of 54K subunit of signal recognition particle. Nature *340*, 482–486.

Chacinska, A., Koehler, C.M., Milenkovic, D., Lithgow, T., and Pfanner, N. (2009). Importing mitochondrial proteins: machineries and mechanisms. Cell *138*, 628–644.

Chin, J.W., Martin, A.B., King, D.S., Wang, L., and Schultz, P.G. (2002). Addition of a photocrosslinking amino acid to the genetic code of Escherichiacoli. Proc. Natl. Acad. Sci. USA *99*, 11020–11024.

Cross, B.C.S., Sinning, I., Luirink, J., and High, S. (2009). Delivering proteins for export from the cytosol. Nat. Rev. Mol. Cell Biol. *10*, 255–264.

de Felipe, P., Luke, G.A., Hughes, L.E., Gani, D., Halpin, C., and Ryan, M.D. (2006). E unum pluribus: multiple proteins from a self-processing polyprotein. Trends Biotechnol. *24*, 68–75.

Deng, H.-X., Chen, W., Hong, S.-T., Boycott, K.M., Gorrie, G.H., Siddique, N., Yang, Y., Fecto, F., Shi, Y., Zhai, H., et al. (2011). Mutations in UBQLN2 cause dominant X-linked juvenile and adult-onset ALS and ALS/dementia. Nature *477*, 211–215.

Doi, H., Mitsui, K., Kurosawa, M., Machida, Y., Kuroiwa, Y., and Nukina, N. (2004). Identification of ubiquitin-interacting proteins in purified polyglutamine aggregates. FEBS Lett. *571*, 171–176.

Finley, D. (2009). Recognition and processing of ubiquitin-protein conjugates by the proteasome. Annu. Rev. Biochem. *78*, 477–513.

Fons, R.D., Bogert, B.A., and Hegde, R.S. (2003). Substrate-specific function of the translocon-associated protein complex during translocation across the ER membrane. J. Cell Biol. *160*, 529–539.

Ghaboosi, N., and Deshaies, R.J. (2007). A conditional yeast E1 mutant blocks the ubiquitin-proteasome pathway and reveals a role for ubiquitin conjugates in targeting Rad23 to the proteasome. Mol. Biol. Cell *18*, 1953–1963.

Harbauer, A.B., Opalińska, M., Gerbeth, C., Herman, J.S., Rao, S., Schönfisch, B., Guiard, B., Schmidt, O., Pfanner, N., and Meisinger, C. (2014). Mitochondria. Cell cycle-dependent regulation of mitochondrial preprotein translocase. Science *346*, 1109–1113.

Hegde, R.S., and Keenan, R.J. (2011). Tail-anchored membrane protein insertion into the endoplasmic reticulum. Nat. Rev. Mol. Cell Biol. *12*, 787–798.

Hessa, T., Sharma, A., Mariappan, M., Eshleman, H.D., Gutierrez, E., and Hegde, R.S. (2011). Protein targeting and degradation are coupled for elimination of mislocalized proteins. Nature *475*, 394–397.

Ko, H.S., Uehara, T., and Nomura, Y. (2002). Role of ubiquilin associated with protein-disulfide isomerase in the endoplasmic reticulum in stress-induced apoptotic cell death. J. Biol. Chem. *277*, 35386–35392.

Koppen, M., and Langer, T. (2007). Protein degradation within mitochondria: versatile activities of AAA proteases and other peptidases. Crit. Rev. Biochem. Mol. Biol. *42*, 221–242.

Krumpe, K., Frumkin, I., Herzig, Y., Rimon, N., Özbalci, C., Brügger, B., Rapaport, D., and Schuldiner, M. (2012). Ergosterol content specifies targeting of tail-anchored proteins to mitochondrial outer membranes. Mol. Biol. Cell *23*, 3927–3935.

Labbadia, J., and Morimoto, R.I. (2015). The biology of proteostasis in aging and disease. Annu. Rev. Biochem. *84*, 435–464.

Liu, Y., Lü, L., Hettinger, C.L., Dong, G., Zhang, D., Rezvani, K., Wang, X., and Wang, H. (2014). Ubiquilin-1 protects cells from oxidative stress and ischemic stroke caused tissue injury in mice. J. Neurosci. *34*, 2813–2821.

Lowe, E.D., Hasan, N., Trempe, J.F., Fonso, L., Noble, M.E.M., Endicott, J.A., Johnson, L.N., and Brown, N.R. (2006). Structures of the Dsk2 UBL and UBA domains and their complex. Acta Crystallogr. D Biol. Crystallogr. *62*, 177–188.

Mariappan, M., Li, X., Stefanovic, S., Sharma, A., Mateja, A., Keenan, R.J., and Hegde, R.S. (2010). A ribosome-associating factor chaperones tail-anchored membrane proteins. Nature *466*, 1120–1124.

Mateja, A., Szlachcic, A., Downing, M.E., Dobosz, M., Mariappan, M., Hegde, R.S., and Keenan, R.J. (2009). The structural basis of tail-anchored membrane protein recognition by Get3. Nature *461*, 361–366.

Mateja, A., Paduch, M., Chang, H.-Y., Szydlowska, A., Kossiakoff, A.A., Hegde, R.S., and Keenan, R.J. (2015). Protein targeting. Structure of the Get3 targeting factor in complex with its membrane protein cargo. Science *347*, 1152–1155.

Mori, F., Tanji, K., Odagiri, S., Toyoshima, Y., Yoshida, M., Ikeda, T., Sasaki, H., Kakita, A., Takahashi, H., and Wakabayashi, K. (2012). Ubiquilin immunoreactivity in cytoplasmic and nuclear inclusions in synucleinopathies, polyglutamine diseases and intranuclear inclusion body disease. Acta Neuropathol. *124*, 149–151.

Neupert, W. (1997). Protein import into mitochondria. Annu. Rev. Biochem. *66*, 863–917.

Olzscha, H., Schermann, S.M., Woerner, A.C., Pinkert, S., Hecht, M.H., Tartaglia, G.G., Vendruscolo, M., Hayer-Hartl, M., Hartl, F.U., and Vabulas, R.M. (2011). Amyloid-like aggregates sequester numerous metastable proteins with essential cellular functions. Cell *144*, 67–78.

Ramdzan, Y.M., Polling, S., Chia, C.P.Z., Ng, I.H.W., Ormsby, A.R., Croft, N.P., Purcell, A.W., Bogoyevitch, M.A., Ng, D.C.H., Gleeson, P.A., and Hatters, D.M. (2012). Tracking protein aggregation and mislocalization in cells with flow cytometry. Nat. Methods *9*, 467–470.

Ran, F.A., Hsu, P.D., Wright, J., Agarwala, V., Scott, D.A., and Zhang, F. (2013). Genome engineering using the CRISPR-Cas9 system. Nat. Protoc. *8*, 2281–2308.

Rodrigo-Brenni, M.C., Gutierrez, E., and Hegde, R.S. (2014). Cytosolic quality control of mislocalized proteins requires RNF126 recruitment to Bag6. Mol. Cell *55*, 227–237.

Rutherford, N.J., Lewis, J., Clippinger, A.K., Thomas, M.A., Adamson, J., Cruz, P.E., Cannon, A., Xu, G., Golde, T.E., Shaw, G., et al. (2013). Unbiased screen reveals ubiquilin-1 and -2 highly associated with huntingtin inclusions. Brain Res. *1524*, 62–73.

Safren, N., El Ayadi, A., Chang, L., Terrillion, C.E., Gould, T.D., Boehning, D.F., and Monteiro, M.J. (2014). Ubiquilin-1 overexpression increases the lifespan and delays accumulation of Huntingtin aggregates in the R6/2 mouse model of Huntington's disease. PLoS ONE *9*, e87513.

Schmidt, O., Harbauer, A.B., Rao, S., Eyrich, B., Zahedi, R.P., Stojanovski, D., Schönfisch, B., Guiard, B., Sickmann, A., Pfanner, N., and Meisinger, C. (2011). Regulation of mitochondrial protein import by cytosolic kinases. Cell *144*, 227–239.

Schuldiner, M., Metz, J., Schmid, V., Denic, V., Rakwalska, M., Schmitt, H.D., Schwappach, B., and Weissman, J.S. (2008). The GET complex mediates insertion of tail-anchored proteins into the ER membrane. Cell *134*, 634–645.

Setoguchi, K., Otera, H., and Mihara, K. (2006). Cytosolic factor- and TOM-independent import of C-tail-anchored mitochondrial outer membrane proteins. EMBO J. *25*, 5635–5647.

Shao, S., and Hegde, R.S. (2011). A calmodulin-dependent translocation pathway for small secretory proteins. Cell *147*, 1576–1588.

Sharma, A., Mariappan, M., Appathurai, S., and Hegde, R.S. (2010). In vitro dissection of protein translocation into the mammalian endoplasmic reticulum. Methods Mol. Biol. *619*, 339–363.

Shiau, A.K., Harris, S.F., Southworth, D.R., and Agard, D.A. (2006). Structural Analysis of E. coli hsp90 reveals dramatic nucleotide-dependent conformational rearrangements. Cell *127*, 329–340.

Shimizu, Y., and Ueda, T. (2010). PURE technology. Methods Mol. Biol. *607*, 11–21.

Shimizu, Y., Inoue, A., Tomari, Y., Suzuki, T., Yokogawa, T., Nishikawa, K., and Ueda, T. (2001). Cell-free translation reconstituted with purified components. Nat. Biotechnol. *19*, 751–755.

Stefanovic, S., and Hegde, R.S. (2007). Identification of a targeting factor for posttranslational membrane protein insertion into the ER. Cell *128*, 1147–1159.

Su, V., and Lau, A.F. (2009). Ubiquitin-like and ubiquitin-associated domain proteins: significance in proteasomal degradation. Cell. Mol. Life Sci. *66*, 2819–2833.

Suzuki, R., and Kawahara, H. (2016). UBQLN4 recognizes mislocalized transmembrane domain proteins and targets these to proteasomal degradation. EMBO Rep., Published online April 22, 2016. http://dx.doi.org/10.15252/embr.201541402.

Wang, X., and Chen, X.J. (2015). A cytosolic network suppressing mitochondria-mediated proteostatic stress and cell death. Nature *524*, 481–484.

Wang, H., and Monteiro, M.J. (2007). Ubiquilin interacts and enhances the degradation of expanded-polyglutamine proteins. Biochem. Biophys. Res. Commun. *360*, 423–427.

Wang, F., Brown, E.C., Mak, G., Zhuang, J., and Denic, V. (2010). A chaperone cascade sorts proteins for posttranslational membrane insertion into the endoplasmic reticulum. Mol. Cell *40*, 159–171.

Wolff, S., Weissman, J.S., and Dillin, A. (2014). Differential scales of protein quality control. Cell *157*, 52–64.

Wright, G., Terada, K., Yano, M., Sergeev, I., and Mori, M. (2001). Oxidative stress inhibits the mitochondrial import of preproteins and leads to their degradation. Exp. Cell Res. *263*, 107–117.

Wrobel, L., Topf, U., Bragoszewski, P., Wiese, S., Sztolsztener, M.E., Oeljeklaus, S., Varabyova, A., Lirski, M., Chroscicki, P., Mroczek, S., et al. (2015). Mistargeted mitochondrial proteins activate a proteostatic response in the cytosol. Nature *524*, 485–488.

Young, J.C., Hoogenraad, N.J., and Hartl, F.U. (2003). Molecular chaperones Hsp90 and Hsp70 deliver preproteins to the mitochondrial import receptor Tom70. Cell *112*, 41–50.

Zhang, D., Raasi, S., and Fushman, D. (2008). Affinity makes the difference: nonselective interaction of the UBA domain of ubiquilin-1 with monomeric ubiquitin and polyubiquitin chains. J. Mol. Biol. *377*, 162–180.

Zhu, X., Zhao, X., Burkholder, W.F., Gragerov, A., Ogata, C.M., Gottesman, M.E., and Hendrickson, W.A. (1996). Structural analysis of substrate binding by the molecular chaperone DnaK. Science *272*, 1606–1614.

Molecular Cell, Volume *63*

Supplemental Information

Ubiquilins Chaperone and Triage Mitochondrial Membrane Proteins for Degradation

Eisuke Itakura, Eszter Zavodszky, Sichen Shao, Matthew L. Wohlever, Robert J. Keenan, and Ramanujan S. Hegde

Itakura, Fig. S1 (related to Fig. 1)

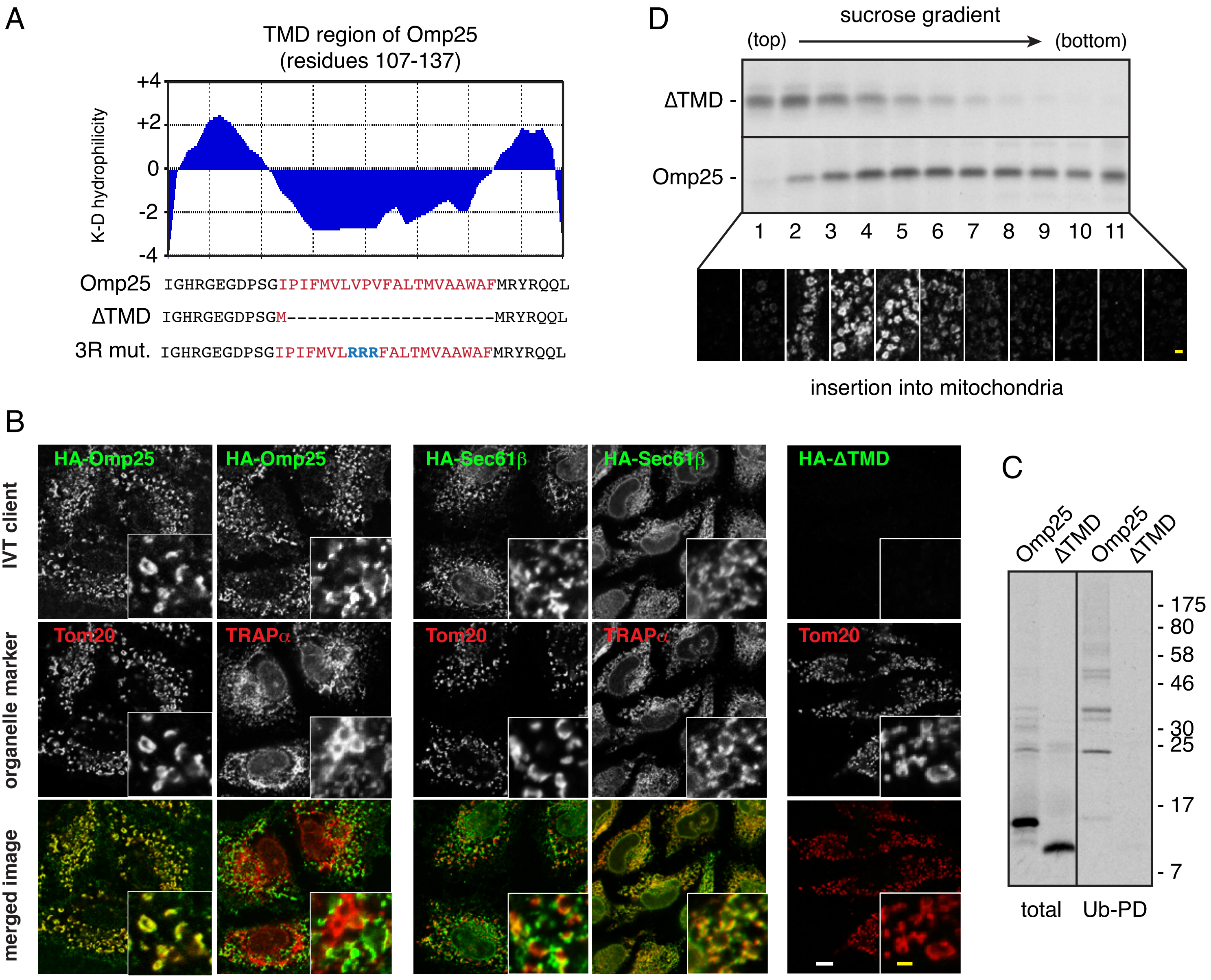

Fig. S1 (related to Fig. 1). Characterization of Omp25 targeting and ubiquitination. (**A**) Sequence and hydrophilicity plot of the TMD region used in all of the Omp25 constructs analyzed in this study. Sequences of the ΔTMD and 3R mutants are also shown. In a series of preliminary experiments, we confirmed that this TMD region at the C-terminus is necessary and sufficient for Omp25 targeting to mitochondria, and for its ubiquitination. Thus, the N-terminal soluble domain of Omp25 could therefore be replaced with any other soluble domains without consequences for targeting or ubiquitination. We therefore produced a number of different N-terminally tagged versions of the Omp25 TMD region (see Table S1) and used them essentially interchangeably as dictated by the requirements for any particular experiment. (**B**) HA-tagged constructs encoding the TMD region of Omp25 or full length Sec61β were translated in reticulocyte lysate and applied to semi-permeabilized HeLa cells. After the incubation, the cells were washed, fixed, and immunostained for the HA tag (green) or an organelle marker (red; Tom20 for mitochondria, or TRAPα for ER). Insets show an enlarged region of one of the cells. A matched ΔTMD control for Omp25 is also shown for comparison. All images were captured using identical settings. Note that mitochondrial targeting of Omp25 is dependent on the hydrophobic domain of the TMD, and that Omp25 and Sec61β are targeted selectively to mitochondria or ER, respectively. (**C**) Matched constructs containing the TMD region of Omp25 or the ΔTMD mutant were translated in reticulocyte lysate containing ^{35}S-methionine and His-tagged Ubiquitin. The samples were either analyzed directly (total) or after isolation of the ubiquitinated products via the His tag (Ub-PD). The products were detected by autoradiography. Note that Omp25 ubiquitination is dependent on the hydrophobic domain of the TMD. (**D**) Constructs containing the Omp25 TMD or the ΔTMD mutant were translated in reticulocyte lysate containing ^{35}S-methionine and separated on a 5-25% sucrose gradient into 11 fractions. Note that while the ΔTMD construct migrates near the top of the gradient consistent with its small size, Omp25 migrates heterogeneously deeper into the gradient, consistent with its engagement in higher molecular weight complexes. In a separate non-radioactive translation reaction of Omp25 fractionated by the same method, each fraction was applied to semi-permeabilized cells and monitored for mitochondrial targeting by immunostaining as in panel B. Note that Omp25-containing complexes in fractions 3-6 display high insertion activity, while complexes in fractions 7-11 are largely inactive. For reference, a native protein of ~60-80 kD would have its peak in fraction 4.

Itakura, Fig. S2 (related to Fig. 1)

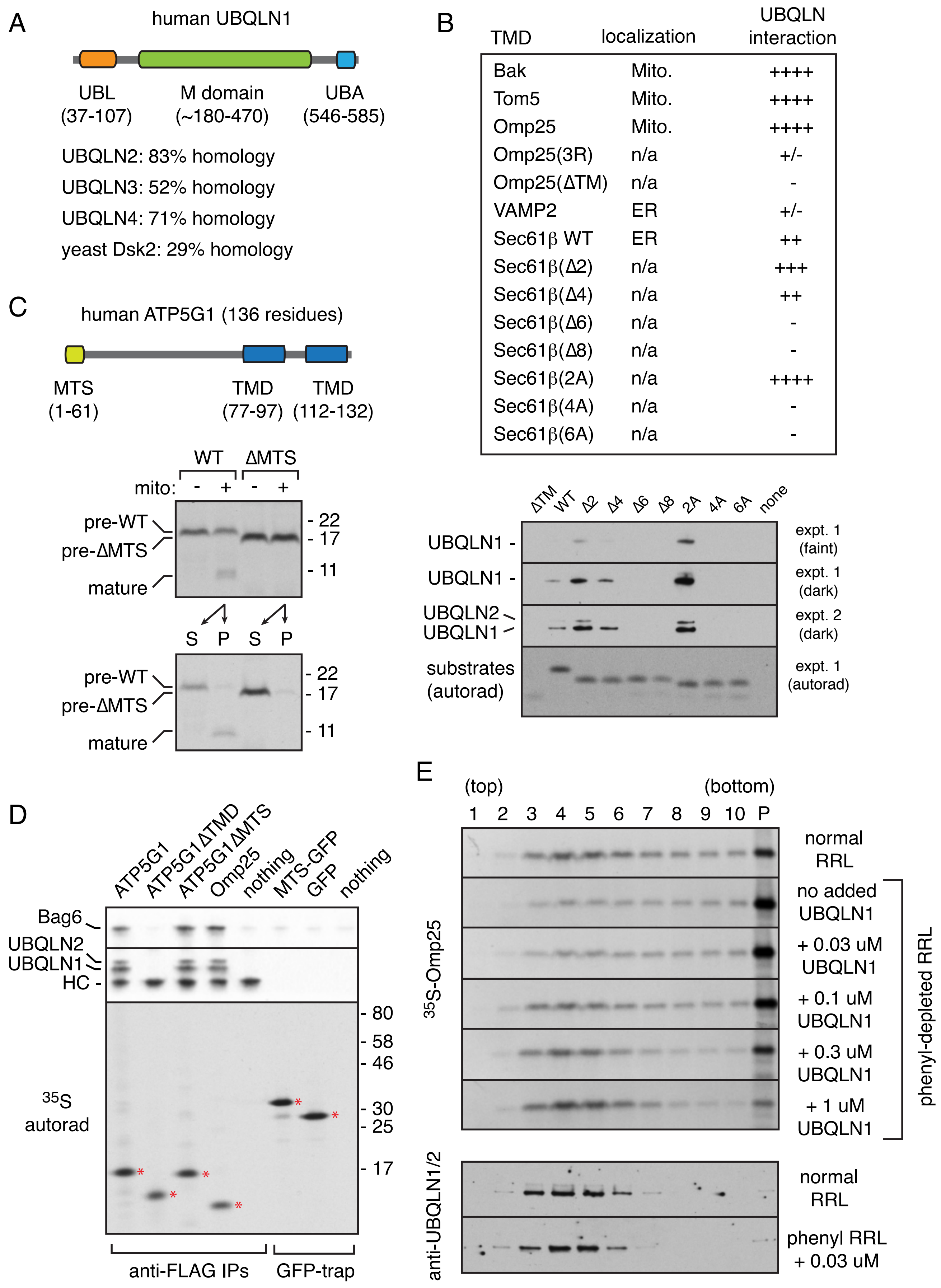

TMD	localization	UBQLN interaction
Bak	Mito.	++++
Tom5	Mito.	++++
Omp25	Mito.	++++
Omp25(3R)	n/a	+/-
Omp25(ΔTM)	n/a	-
VAMP2	ER	+/-
Sec61β WT	ER	++
Sec61β(Δ2)	n/a	+++
Sec61β(Δ4)	n/a	++
Sec61β(Δ6)	n/a	-
Sec61β(Δ8)	n/a	-
Sec61β(2A)	n/a	++++
Sec61β(4A)	n/a	-
Sec61β(6A)	n/a	-

Fig. S2 (related to Fig. 1). Characterization of UBQLN-client interactions. (**A**) Schematic of human Ubiquilin 1 architecture showing the relative positions of the key domains. UBL is the ubiquitin-like domain, UBA is the ubiquitin-associating domain, and the middle "M" domain is the highly methionine-rich region within which putative "Sti1" motifs of uncertain function have been suggested. Although Sti1 domains can potentially bind to heat shock proteins, we have yet to detect such an interaction with UBQLNs in our studies. Percent homology (the sum of identical and similar residues) with other human UBQLN family members and yeast Dsk2 are indicated. (**B**) Table summarizing the relative interactions of the indicated TMDs with UBQLNs as assayed in Fig. 1B and 1C. Bak, Tom5, and Omp25 are all mitochondrially targeted tail-anchored membrane proteins. VAMP2 and Sec61β are ER targeted tail-anchored proteins. The various mutants of Sec61β decrease the hydrophobicity of its TMD by either deleting residues or replacing them with Alanine. The gels below the table show the primary data from two independent experiments for this series of Sec61β TMD constructs. Two exposures of the immunoblot are shown for experiment 1 to better illustrate differences among the strongest interactors. The sum of these analyses indicate that UBQLN interaction depends critically on hydrophobicity of sufficient length to be a TMD, and is disfavored from binding the most hydrophobic TMDs (which are Sec61β and VAMP2). (**C**) Domain diagram of human ATP5G1, an inner mitochondrial membrane protein subunit of ATP synthase, showing its N-terminal mitochondrial targeting sequence (MTS) and two TMDs. The gels show that ATP5G1 is imported into mitochondria in vitro, where its MTS is removed. The wild type (WT) or ΔMTS mutant (lacking residues 1-25) were translated in reticulocyte lysate (RRL) containing ^{35}S-methionine, then incubated with isolated yeast mitochondria. Aliquots of the total products of these reactions are shown in the top gel. In parallel, the products of an insertion reaction were separated into a soluble supernatant (S) and a mitochondrial pellet (P), and shown in the bottom gel. Mature ATP5G1 co-sediments with mitochondria, while the ΔMTS mutant completely prevents import and processing. (**D**) The indicated constructs were translated in RRL containing ^{35}S-methionine, affinity purified, and analyzed by immunoblotting and autoradiography. Negative controls lacking substrate were analyzed in parallel. ATP5G1, its deletion mutants, and Omp25 were FLAG tagged, while the isolated MTS (from cytochrome c) was GFP-tagged. Note that ATP5G1 interacts with Ubiquilins as efficiently as Omp25, and this interaction is dependent on the TMDs. The MTS of neither ATP5G1 nor cytochrome c interacts with UBQLNs. HC indicates IgG heavy chain. (**E**) A construct containing the Omp25 TMD region was translated in RRL containing ^{35}S-methionine and separated on a 5-25% sucrose gradient into 10 fractions and the high molecular weight pellet (P). Translation was performed in either complete RRL or RRL that had been passed over phenyl sepharose to deplete most hydrophobic binding proteins (including UBQLNs, TRC40, Bag6, and SGTA). The phenyl-depleted RRL was supplemented with recombinant UBQLN1 to the indicated concentrations before use in the translation reaction. Note that the amount of Omp25 in the insoluble pellet is increased in phenyl-depleted RRL (with a corresponding decrease from fractions 3-6), and this behavior is progressively rescued by increasing concentrations of UBLQN1. The immunoblots below show the position of UBQLN1 migration in the gradient, illustrating that endogenous and recombinant UBLQN1 migrate similarly. Note that at the low concentration of 0.03 mM, all of the UBQLN1 is expected to be fully engaged by Omp25, indicating that the UBQLN1-Omp25 complex is likely to contain a single UBLQN1.

Itakura, Fig. S3 (related to Fig. 2)

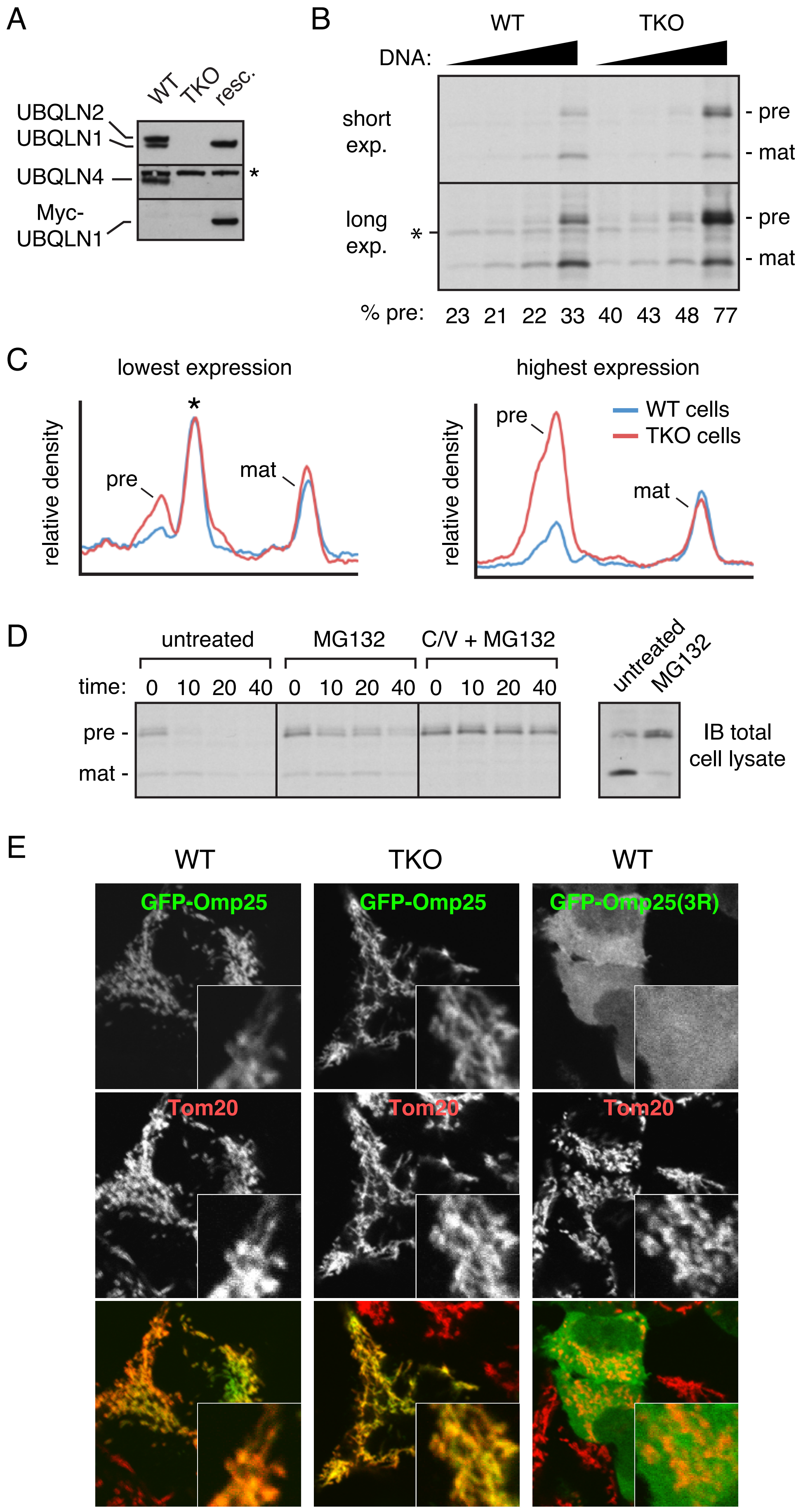

Fig. S3 (related to Fig. 2). Characterization of UBQLN triple knockout cells. (**A**) Immunoblotting of parental (WT), UBQLN1/2/4 triple knockout (TKO), and Myc-UBQLN1 rescued (resc.) HEK293 cells. Asterisk indicates a background band that serves as a loading control. (**B**) WT and TKO cells were transfected with a mixture of ATP5G1-HA and GFP expression plasmids in different ratios (1:5, 2:4, 3:3, and 6:0) to modulate the relative expression of ATP5G1. These cells were pulse labelled for 30 min with ^{35}S-methionine, the ATP5G1-HA was immunoprecipitated, and products were visualized by SDS-PAGE and autoradiography. The positions of precursor and mature forms of ATP5G1-HA are indicated. Short and long exposures of the gel are shown to visualize the highest and lowest expression samples, respectively. The asterisk indicates a background band (probably one of the histone proteins) recovered due to non-specific binding to the beads. Phosphorimaging was used to quantify the relative levels of precursor and mature forms in each sample and determine the percent of precursor (indicated below the gel). Note that the absolute amount of mature protein is very similar between WT and TKO cells across all expression levels. By contrast, the amount of precursor (and hence, the % precursor) is consistently higher in the TKO cells, consistent with its impaired degradation during the period of pulse labelling. Expression of ATP5G1-HA spanned a ~40-fold range (as determined by phosphorimaging) from the lowest to highest levels. This indicates that UBQLN-mediated precursor degradation is occurring at a variety of expression levels, and consistently accounts for ~15-20% of total synthesized ATP5G1-HA. This demand is elevated at the highest expression level, presumably due to some saturation of the import machinery. (**C**) Densitometry traces of the lowest (left) and highest (right) expression lanes from panel B illustrating the preferential increase in precursor in TKO cells (red traces). The positions of precursor (pre) and mature (mat) forms of ATP5G1-HA are indicated, as is the background band (asterisk). (**D**) WT cells expressing ATP5G1-HA were pulse-labeled with ^{35}S-methionine for 5 min and chased for up to 40 min with unlabeled methionine. Immunoprecipitated ATP5G1-HA was visualized by autoradiography. Where indicated, the proteasome was inhibited with MG132 and mitochondrial import was inhibited with a mixture of CCCP and Valinomycin (C/V). The positions of precursor (pre) and mature (mat) forms of ATP5G1 are indicated. Quantification of the MG132-treated pulse-chase samples by phosphorimaging indicates that ~25% of initially synthesized ATP5G1-HA is ordinarily degraded by a proteasome-dependent pathway. This pulse-chase experiment is part of the same experiment shown in Fig. 2B, and the 'C/V + MG132' panel for WT cells is duplicated here for comparison. The right panel shows an anti-HA immunoblot of untreated and MG132-treated (for 5 h) total cell lysate to detect steady state levels of ATP5G1-HA. The lower level of mature form in the MG132 treated lane of the immunoblot is due to some inhibition of protein synthesis during chronic proteasome inhibition. The important observation here is the preferential stabilization of the precursor, consistent with the pulse-chase results. (**E**) GFP-Omp25 and the 3R mutant were transiently transfected into WT and TKO cells as indicated, and their localization relative to mitochondria (stained with anti-Tom20) was determined by confocal microscopy. Note that in TKO cells, mitochondrial morphology is grossly normal, and that GFP-Omp25 is localized correctly and quantitatively. Comparison of the amount of GFP-Omp25 at mitochondria across numerous WT versus TKO cells showed no systematic differences, indicating that its successful targeting is similar in these two cell lines.

Itakura, Fig. S4 (related to Fig. 2)

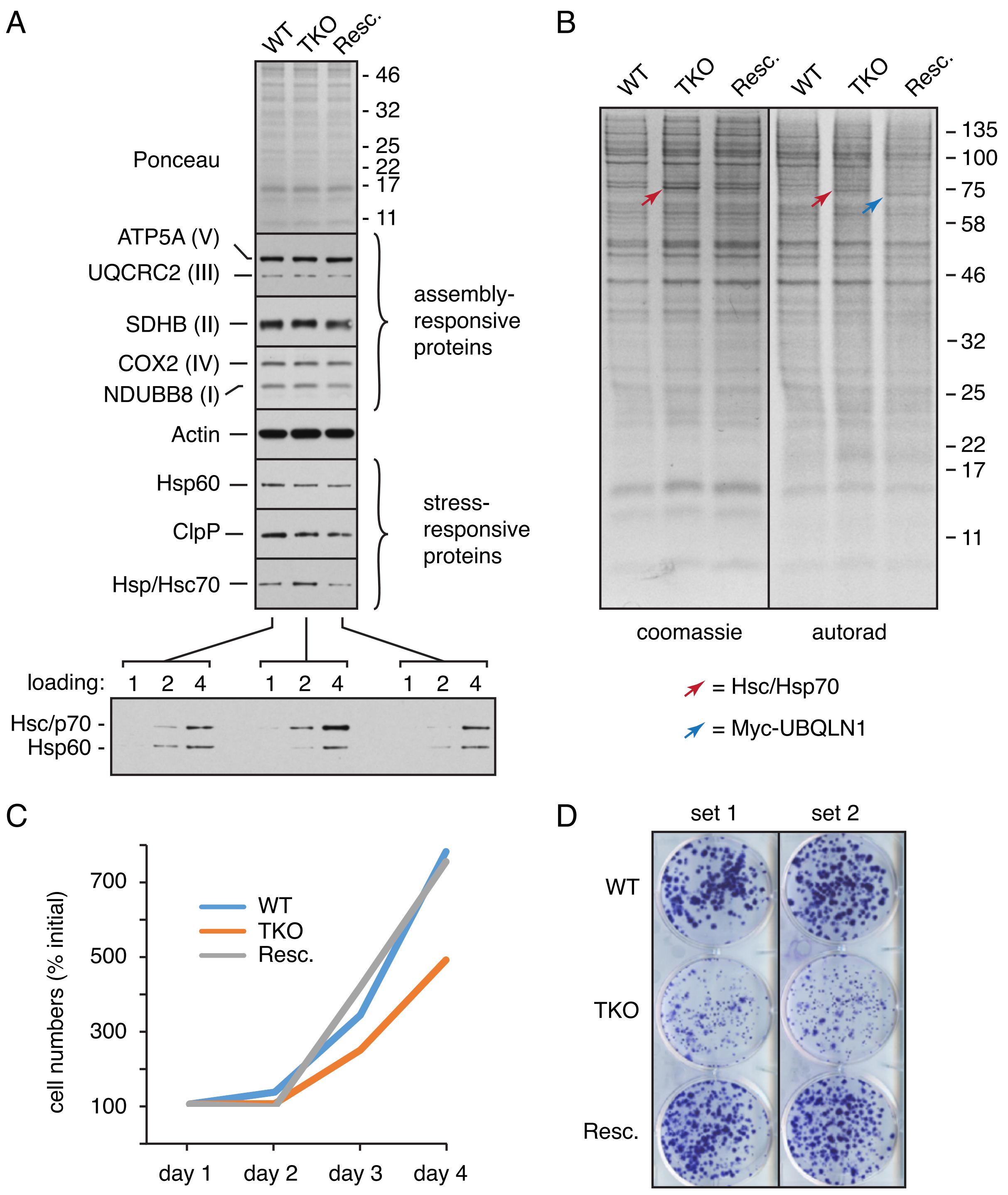

Fig. S4 (related to Fig. 2). Characterization of UBQLN triple knockout cells. (**A**) Equal amounts of total cell lysate prepared from WT, TKO, and rescue cells were analyzed by SDS-PAGE, blotted, and visualized for total protein with Ponceau stain (top panel) and the indicated antigens were detected by immunostaining. The five 'assembly-responsive proteins' are each core components of respiratory complexes I through V (all detected using the human OxPhos cocktail of monoclonal antibodies from Abcam) whose levels are sensitive to deficiencies in assembly of the respective complexes. Hsp60 and ClpP are mitochondrial proteins whose levels are increased by mitochondrial stress, while Hsp70 levels increase during cytosolic stress. The only protein systematically observed in multiple independent samples to be selectively changed in TKO cells was Hsc70/Hsp70 (the antibody detects both isoforms), as verified by analysis of serial dilutions of the three cell lysates (bottom panel) immunoblotted simultaneously for Hsc/Hsp70 and Hsp60. The numbers above the lanes in the bottom panel indicate the relative amounts loaded in each lane. (**B**) WT, TKO, and rescue cells were pulse labeled for 5 min with ^{35}S-methionine and analyzed by SDS-PAGE and coomassie staining (left panel) or autoradiography (right panel). The red arrow points to an abundant 70 kD protein (which we presume is Hsp70 based on the blots in panel A) whose levels are increased selectively in TKO cells at steady state (left panel) and whose rate of synthesis is notably higher (right panel). This indicates that the TKO cells are actively experiencing cytosolic stress. The blue arrow points to Myc-UBQLN1, whose labeling is unusually high due to its very high methionine content. UBQLNs are not easily discernable in the WT cells because the signal is distributed among UBQLN1/2/4. (**C**) Growth rate of WT, TKO, and rescue cells measured over four days after seeding. (**D**) Size of colonies of WT, TKO, and rescue cells formed over 14 days of growth after 100 cells were seeded into individual wells of a 6-well plate. Two replicates are shown.

Itakura, Fig. S5 (related to Fig. 3)

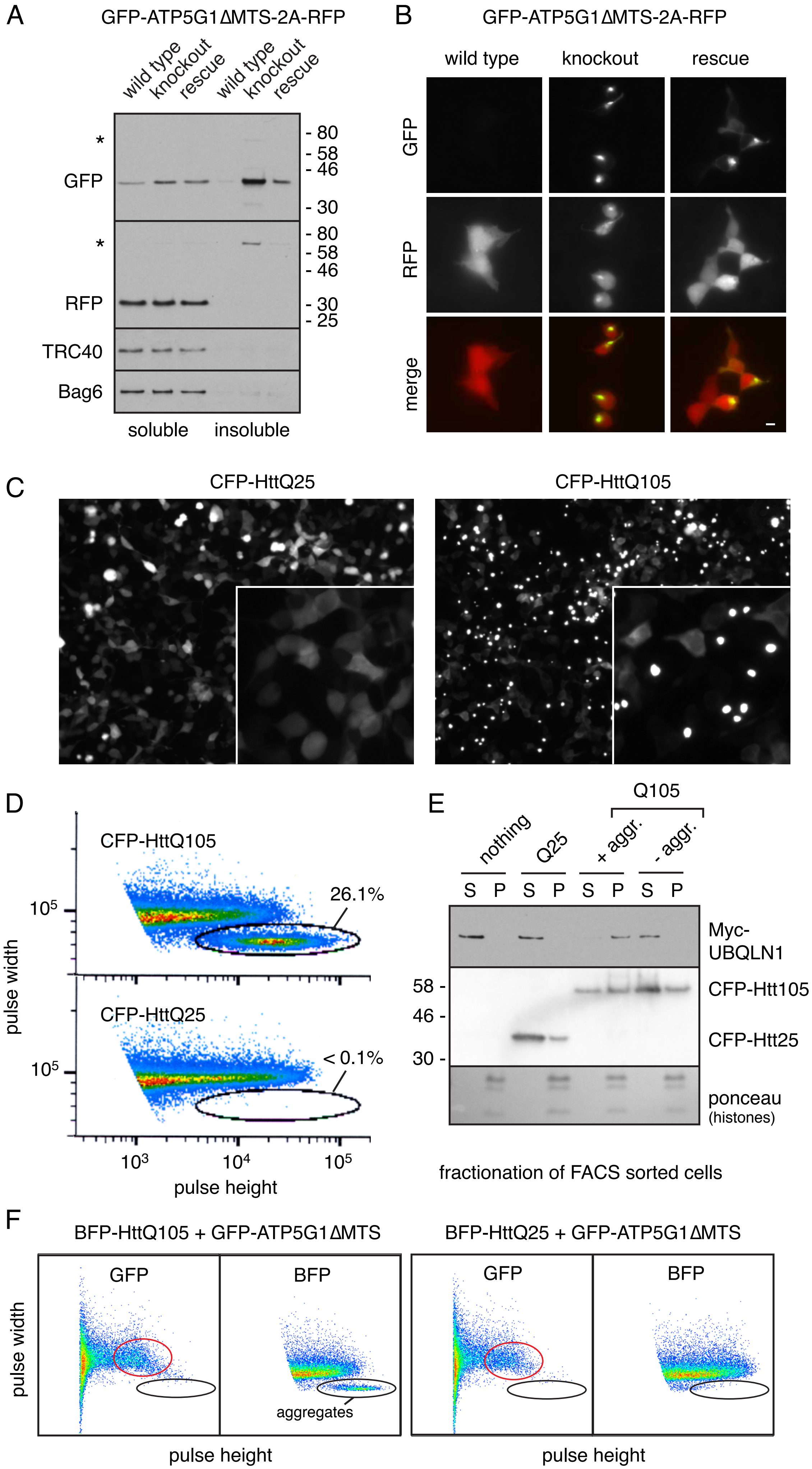

Fig. S5 (related to Fig. 3). Characterization of protein aggregation. (**A**) GFP-ATP5G1ΔMTS-2A-RFP was transfected into the indicated HEK293 cell lines, the cells harvested in non-denaturing detergent, and the lysates separated into soluble and insoluble fractions. The samples were blotted for GFP, RFP, TRC40, and Bag6. Note that the vast majority of translated RFP is appropriately separated at the 2A sequence, with only a minor amount of detectable fusion protein (asterisks in the GFP and RFP blots). As expected, the RFP is uniformly soluble in wild type, UBQLN triple knockout (TKO), and UBQLN1 rescue cells. By contrast, the GFP-ATP5G1ΔMTS fusion protein behaves differently in the three cell lines. Not only is the overall level higher in TKO cells, but a substantial proportion is found in the insoluble fraction, presumably representing aggregated protein. The increased levels and aggregation are partially reversed in knockout cells re-expressing UBQLN1. Note that the small amount of uncleaved GFP-ATP5G1ΔMTS-2A-RFP protein (asterisk) also accumulates preferentially in the insoluble fraction in TKO cells. TRC40 and Bag6 remain uniformly soluble in all cell lines and serve as fractionation and loading controls. (**B**) Cells transfected as in panel A were visualized by fluorescence microscopy to detect the localization of GFP-ATP5G1ΔMTS and RFP. Consistent with the biochemical analysis in panel A, RFP is uniformly distributed in the nucleocytoplasmic compartment of all cells. By contrast, the GFP signal is increased in knockout cells, where it is seen in puncta consistent with its aggregation. This is partially reversed in some, but not all cells upon UBQLN1 rescue. Scale bar is 10 μm. (**C**) View of a field of cells (with one region enlarged in the inset) of cells transfected with CFP-HttQ25 or CFP-HttQ105, illustrating that the latter is preferentially seen in puncta in many, but not all cells. (**D**) Aggregate-containing cells expressing CFP-HttQ105 can be separated from aggregate-lacking cells by flow cytometry using their differences in pulse height versus width. As expected from panel C, CFP-HttQ25 does not form aggregates. Quantification indicates ~25-30% of HttQ105-expressing cells contain aggregates in any typical experiment. (**E**) HEK293 rescue cells (knocked out for UBQLN1/2/4 but rescued with UBQLN1 re-expression) were transfected with CFP-HttQ25 or CFP-HttQ105. The CFP-HttQ105 cells were further separated by FACS into cells containing or lacking aggregates (as in panel D). Each set of cells was lysed in non-denaturing detergent, separated by centrifugation into supernatant (S) and pellet (P) fractions, and analyzed by immunoblotting and Ponceau-staining (to visualize the nuclear histones). Note that the majority of UBQLN1 is soluble, and this property does not change with expression of CFP-HttQ25. By contrast, cells containing CFP-HttQ105 aggregates result in most of the UBQLN1 now residing in the pellet fraction. This re-distribution was not seen in cells lacking aggregates, but nonetheless expressing CFP-HttQ105 at comparable levels. (**F**) HEK293 cells co-transfected with GFP-ATP5G1ΔMTS-2A-RFP and either BFP-HttQ105 or BFP-HttQ25 were analyzed by flow cytometry in both color channels (BFP and GFP) for pulse height versus width as in panel D. Aggregates of BFP-HttQ105, seen as a narrower pulse width relative to height, are not observed for HttQ25. Importantly, co-expressed GFP-ATP5G1 in these same cells does not show the narrow pulse width property in an HttQ105-dependent manner, indicating that the GFP signal is not exclusively co-localized (and hence, not quantitatively co-aggregated with) the BFP signal. The red circle indicates the area where an increased proportion of cells are seen in the HttQ105 cells relative to the HttQ25 cells. These represent cells whose GFP-ATP5G1 levels are increased due to stabilization, and represent specifically the cells containing HttQ105 aggregates (see Fig. 3E).

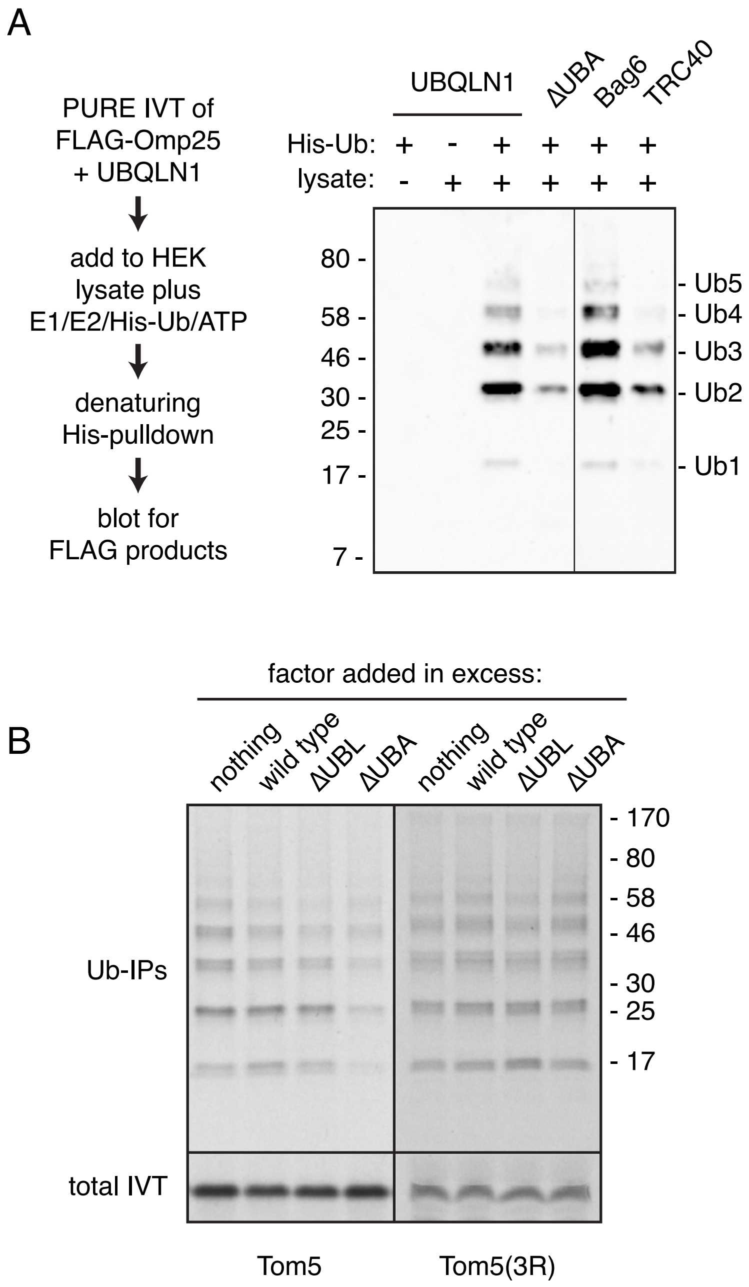
A
PURE IVT of
FLAG-Omp25
+ UBQLN1
add to HEK
lysate plus
E1/E2/His-Ub/ATP
denaturing
His-pulldown
blot for
FLAG products
UBQLN1
ΔUBA
Bag6
TRC40
His-Ub: + - + + + +
lysate: - + + + + +
80 -
58 -
46 -
30 -
25 -
17 -
7 -
- Ub5
- Ub4
- Ub3
- Ub2
- Ub1
B
factor added in excess:
nothing
wild type
ΔUBL
ΔUBA
nothing
wild type
ΔUBL
ΔUBA
- 170
- 80
- 58
- 46
- 30
- 25
- 17
Ub-IPs
total IVT
Tom5
Tom5(3R)

Fig. S6 (related to Fig. 4). Ubiquitination of Omp25 in complex with UBQLN1. (**A**) The left side shows the experimental strategy, and the right side the result. A FLAG-tagged construct containing the Omp25 TMD region was translated in the PURE system supplemented with recombinant UBQLN1 at 15 μM. The resulting UBQLN1-Omp25 complex was added to HEK293 cytosol supplemented with His-tagged Ubiquitin, E1 and E2 enzymes, and ATP. After incubation for 1 h, the ubiquitinated products were recovered via the His tag, and the samples immunoblotted for the FLAG tag. Note that lanes 1-4 were digitally spliced with lanes 5 and 6 to prepare the image. All samples were analyzed together on the same gel and the composite image was produced from the same exposure of the blot. Recovery of ubiquitinated Omp25 is observed to be dependent on both the lysate and His-Ubiquitin. The use of ΔUBA-UBQLN1 or TRC40 as the chaperone for Omp25 results in lower levels of ubiquitination, while Bag6 promotes client ubiquitination as expected from earlier studies. These results suggest that UBQLN1 is able to facilitate ubiquitination of its bound client in a UBA domain-dependent manner. (**B**) The UBQLN-associating client Tom5 and the non-associating Tom5(3R) mutant were translated in RRL containing ^{35}S-methionine, the indicated UBQLN1 protein (at 1 μM), and His-tagged Ubiquitin (at 10 μM). Shown are aliquots of the total translation products and the ubiquitinated products isolated via His-tagged Ubiquitin.

Itakura, Fig. S7 (related to Fig. 5)

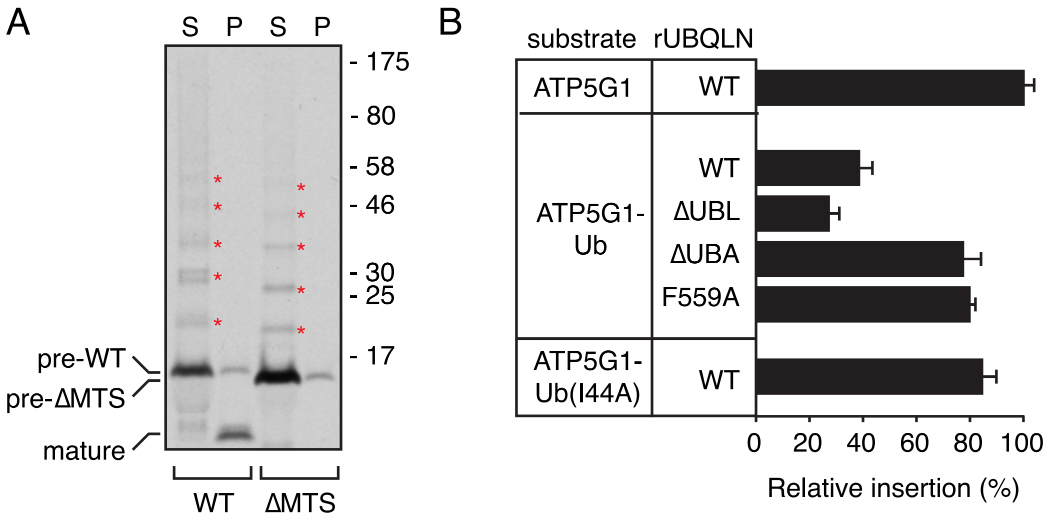

Fig. S7 (related to Fig. 5). Ubiquitinated ATP5G1 is prevented from membrane insertion. (**A**) Wild type (WT) and ΔMTS mutant ATP5G1 were produced in RRL containing ^{35}S-methionine, incubated with isolated mitochondria, separated by centrifugation into cytosolic supernatant (S) and mitochondrial pellet (P) fractions, and analyzed by SDS-PAGE and autoradiography. The positions of the precursor and mature forms of the proteins are indicated. Red asterisks indicate ubiquitinated species. Note that while non-ubiquitinated ATP5G1 is imported into mitochondria as judged by its processing and co-sedimentation, ubiquitinated ATP5G1 remains entirely in the cytosol. (**B**) The indicated substrates were translated in RRL supplemented with the indicated recombinant UBQLN1 proteins (at 1 μM) and analyzed for insertion into the mitochondria of semi-permeabilized cells. The graph shows the relative insertion efficiencies, normalized to that observed for reactions of ATP5G1 containing wild type UBQLN1 (mean ± SD).

Table S1 (related to Experimental Procedures) – List of constructs used in this study.

Construct Name (internal reference)	Purpose	Epitope tag	Functional element	Figure(s)
FH-Omp25 (EI-54)	IVT (RRL)	Tandem 3xFLAG-3xHA @ N-term.	Human Omp25 TMD region	1A, 1B, 5A
FH-Omp25ΔTM (EI-61)	IVT (RRL)	Tandem 3xFLAG-3xHA @ N-term.	Omp25 TMD region with hydrophobic region deleted	1A, 1B, 5A
FH-Omp25(3R) (EI-58)	IVT (RRL)	Tandem 3xFLAG-3xHA @ N-term.	Omp25 TMD region with 3R mutation	1B
FLAG-Omp25 (EI-71)	IVT (RRL)	3xFLAG @ N-term.	Omp25 TMD region	1C, 4C, S2D
FLAG-Omp25(3R) (EI-108)	IVT (RRL)	3xFLAG @ N-term.	Omp25 TMD region with 3R mutation	4C, S2D
β-Omp25 (SS)	IVT (RRL)	Sec61β-cytosolic domain @ N-term.	Omp25 TMD region	S1C, S1D
β-Omp25ΔTM (SS)	IVT (RRL)	Sec61β-cytosolic domain @ N-term.	Omp25 TMD region with hydrophobic region deleted	S1C, S1D
FLAG–β (4010)	IVT (RRL)	3xFLAG + Sec61β-cytosolic domain @ N-term.	Sec61β TMD region	1C
FLAG–β-VAMP2 (SS)	IVT (RRL)	3xFLAG + Sec61β-cytosolic domain @ N-term.	VAMP2 TMD region	1C
HA-Omp25 (EI-77)	IVT (RRL)	3xHA @ N-term.	Omp25 TMD region	S1B
HA-Omp25ΔTM (EI-78)	IVT (RRL)	3xHA @ N-term.	Omp25 TMD region with hydrophobic region deleted	S1B
HA-Sec61β (4009)	IVT (RRL)	3xHA @ N-term.	Human Sec61β	S1B
FLAG-Tom5 (EI-138)	IVT (RRL)	3XFLAG @ N-term.	Human Tom5 TMD region	1C, S6B
FLAG-Tom5(3R)	IVT (RRL)	3XFLAG @ N-term.	Human Tom5 TMD region with 3R mutation (changes residues ALLRVT with ARRRVT in TMD)	S6B
ATP5G1-FLAG (EI-186)	IVT (RRL)	3XFLAG @ C-terminus	Human ATP5G1	S2C, S2D, S7A
ATP5G1(ΔTM)-FLAG (EI-187)	IVT (RRL)	3XFLAG @ C-terminus	Human ATP5G1 with both TMDs deleted (residues 70-135)	S2D
ATP5G1(ΔMTS)-FLAG (EI-188)	IVT (RRL)	3XFLAG @ N-terminus in place of the MTS	Human ATP5G1 with N-terminal MTS disrupted (residues 1-25)	S2C, S2D, S7A
ATP5G1-HA	IVT (RRL)	3xHA @ C-terminus	Human ATP5G1	S7B
ATP5G1-Ub-HA	IVT (RRL)	3xHA @ C-terminus	Human ATP5G1 containing Ubiquitin(G76V) between ATP5G1 and the 3xHA tag.	S7B
ATP5G1-Ub(I44A)-HA	IVT (RRL)	3xHA @ C-terminus	Human ATP5G1 containing Ubiquitin(144A;G76V) between ATP5G1 and the 3xHA tag.	S7B
AcGFP (EI-190)	IVT (RRL)	AcGFP	none	S2D
MTS-AcGFP (EI-189)	IVT (RRL)	AcGFP	Mitochondrial targeting sequence (MTS) from cytochrome B @ N-terminus	S2D
HA-β-Omp25 (EI-197)	IVT (RRL)	3xHA + Sec61β cytosolic domain @ N-term.	Omp25 TMD region	4B, 5B, 7C, S2E
HA-Ub-β-Omp25 (EI-225)	IVT (RRL)	3xHA + Sec61β cytosolic domain @ N-term.	Omp25 TMD region plus Ubiquitin between HA and Sec61β domains	5D, 5F, 7C
HA-Ub(I44A)-β-Omp25 (EI-226)	IVT (RRL)	3xHA + Sec61β cytosolic domain @ N-term.	Omp25 TMD region plus Ubiquitin(I44A) between HA and Sec61β domains	5E, 5F
Sec61β (2935)	IVT (RRL)	None	Wild type human Sec61β	S2B
Sec61β(Δ2) (3554)	IVT (RRL)	None	Two residues deleted from TMD of Sec61β See Mariappan et al., 2010	S2B
Sec61β(Δ4) (3555)	IVT (RRL)	None	Four residues deleted from TMD of Sec61β See Mariappan et al., 2010	S2B
Sec61β(Δ6) (3556)	IVT (RRL)	None	Six residues deleted from TMD of Sec61β See Mariappan et al., 2010	S2B
Sec61β(Δ8) (3557)	IVT (RRL)	None	Eight residues deleted from TMD of Sec61β See Mariappan et al., 2010	S2B
Sec61β(2A) (3558)	IVT (RRL)	None	Two residues of Sec61β TMD changed to Alanine See Mariappan et al., 2010	S2B
Sec61β(4A) (3559)	IVT (RRL)	None	Four residues of Sec61β TMD changed to Alanine See Mariappan et al., 2010	S2B
Sec61β(6A) (3560)	IVT (RRL)	None	Six residues of Sec61β TMD changed to Alanine See Mariappan et al., 2010	S2B
FLAG-Omp25 (EI-117)	IVT (PURE)	3xFLAG @ N-term.	Omp25 TMD region	1D, S6A
HA-Omp25 (EI-118)	IVT (PURE)	3xHA @ N-term.	Omp25 TMD region	5C
HA-β-Omp25 (EI-203)	IVT (PURE)	3xHA + Sec61β-cytosolic domain @ N-term.	Omp25 TMD region	6B
HA-Ub-β-Omp25 (EI-227)	IVT (PURE)	3xHA + Sec61β-cytosolic domain @ N-term.	Omp25 TMD region plus Ubiquitin between HA and Sec61β domains	6C
HA-Ub(I44A)-β-Omp25 (EI-228)	IVT (PURE)	3xHA + Sec61β-cytosolic domain @ N-term.	Omp25 TMD region plus Ubiquitin(I44A) between HA and Sec61β domains	6C
GFP (F2)	Cell expression	Monomeric EGFP (originally from Clontech)	n/a	2E
GFP-Omp25 (EI-113)	Cell expression	Monomeric EGFP	Omp25 TMD region	2E, S3E
GFP-Omp25ΔTM (EI-174)	Cell expression	Monomeric EGFP	Omp25 TMD region with hydrophobic region deleted	2E
GFP-Omp25(3R) (EI-167)	Cell expression	Monomeric EGFP	Omp25 TMD region with 3R mutation	S3E
ATP5G1-HA	Cell expression	3xHA @ C-terminus	ATP5G1	2A, 2B, 2C, 2D,

(EI-156)				2E, S3B-S3D
GFP-ATP5G1ΔMTS-2A-RFP (EI-210)	Cell expression	GFP @ N-terminus; 2A-separated RFP (mCherry variant).	ATP5G1 with its MTS deleted (and replaced by GFP)	3B, 3E, 3F, S5A, S5B, S5F
GFP-2A-RFP (KY)	Cell expression	GFP @ N-terminus; 2A-separated RFP (mCherry variant).	n/a	3B
CFP-HttQ25 (EZ)	Cell expression	CFP (Cerulean variant) at C-terminus.	Human Htt exon1 containing 25 glutamine codons (mixture of CAA and CAG codons) at polymorphic site.	3D, 3E, S5C, S5D, S5E
CFP-HttQ105 (EZ)	Cell expression	CFP (Cerulean variant) at C-terminus.	Human Htt exon1 containing 105 glutamine codons (mixture of CAA and CAG codons) at polymorphic site.	3C, 3D, 3E, S5C, S5D, S5E
BFP-HttQ25 (EZ)	Cell expression	BFP (mTagBFP variant) at C-terminus.	Human Htt exon1 containing 25 glutamine codons (mixture of CAA and CAG codons) at polymorphic site.	S5F
BFP-HttQ105 (EZ)	Cell expression	BFP (mTagBFP variant) at C-terminus.	Human Htt exon1 containing 105 glutamine codons (mixture of CAA and CAG codons) at polymorphic site.	3F, S5F
pX330ΔCas9 (EI-154)	sgRNA cassette	n/a	pX330 from Ran et al. (2013) modified to excise the spCas9 expression cassette.	n/a
pX330Δ-UBQLN1 (EI-93)	sgRNA expression	n/a	pX330ΔCas9 plasmid with sgRNA that targets human UBQLN1 (CGAGAATAGCTCCGTCCAGC)	2A-2E, S3A, 3B, 3C, S5A, S5B, S5E, S4
pX330Δ-UBQLN2 (EI-98)	sgRNA expression	n/a	pX330ΔCas9 plasmid with sgRNA that targets human UBQLN2 (CGCGGGAACTAACACTACCT)	2A-2E, S3A, 3B, 3C, S5A, S5B, S5E, S4
pX330Δ-UBQLN4 (EI-101)	sgRNA expression	n/a	pX330ΔCas9 plasmid with sgRNA that targets human UBQLN4 (CTCTTGATCACAGTTCAAAG)	2A-2E, S3A, 3B, 3C, S5A, S5B, S5E, S4
pCDNA5-Frt-TO-FLAG-spCas9 (EI-128)	Cell expression	3xFLAG tag at N-terminus	Humanized S. pyogenes Cas9 in plasmid for stable-inducible expression in HEK293-Flp-in-TREX cells.	2A-2E, S3A, 3B, S5A, S5B, S4
pCDNA5-Frt-TO-Myc-UBQLN1 (EI-141)	Cell expression	3xMyc tag at N-terminus	Human UBQLN1 with 3xMyc at N-terminus in plasmid for stable-inducible expression in HEK293-Flp-in-TREX cells.	2B-2E, S3A, 3B, 3C, S5A, S5B, S5E, S4
pCDNA5-Frt-TO-Bag6-3C-FLAG (3444)	Cell expression	3C-FLAG tag at C-terminus	Human Bag6; See Hessa et al., 2011	S6A
His6-UBQLN1 (p24)	Bacterial expression	His6-tag at N-terminus	pCOLAduet vector containing human UBQLN1 tagged at N-terminus with His6-tag.	1D, S6A, 4C, S6B
His6-UBQLN1ΔUBL (p46)	Bacterial expression	His6-tag at N-terminus	pCOLAduet vector containing human UBQLN1ΔUBL tagged at N-terminus with His6-tag. Residues 2-107 deleted.	1D, 4C, S6B
His6-UBQLN1ΔUBA (p47)	Bacterial expression	His6-tag at N-terminus	pCOLAduet vector containing human UBQLN1ΔUBA tagged at N-terminus with His6-tag. Residues 547-589 deleted.	1D, S6A, 4C, S6B
His6-UBQLN1ΔM (p59)	Bacterial expression	His6-tag at N-terminus	pCOLAduet vector containing human UBQLN1 tagged at N-terminus with His6-tag. Residues 182-251 and 387-470 deleted.	1D, 4C
His6-3C-FLAG-UBQLN1 (EI-213)	Bacterial expression	His6-3C-3xFLAG at N-terminus	pCOLAduet vector containing human UBQLN1 tagged at N-terminus with His6-3C-FLAG sequence.	4B, 5C, 5D, 5E, 5F, 6, 7A, 7C, S7B
His6-3C-FLAG-UBQLN1ΔUBL (EI-214)	Bacterial expression	His6-3C-3xFLAG at N-terminus	pCOLAduet vector; human UBQLN1 tagged at N-terminus with His6-3C-FLAG sequence. Residues 2-107 deleted.	4B, 5F, 7A, 7C, S7B
His6-3C-FLAG-UBQLN1ΔUBA (EI-215)	Bacterial expression	His6-3C-3xFLAG at N-terminus	pCOLAduet vector; human UBQLN1 tagged at N-terminus with His6-3C-FLAG sequence. Residues 547-589 deleted.	4B, 5D, 5F, 7A, 7C, S7B
His6-3C-FLAG-UBQLN1(F559) (EI-229)	Bacterial expression	His6-3C-3xFLAG at N-terminus	pCOLAduet vector; human UBQLN1 tagged at N-terminus with His6-3C-FLAG sequence. F559A mutation in UBA domain.	5F, S7B
GST-3C-SGTA (3685)	Bacterial expression	GST-3C at N-terminus	Human SGTA. See Mateja et al., 2015	1D, 5C
His6-TEV-TRC40 (3567)	Bacterial expression	His6-TEV at N-terminus	Zebrafish TRC40	S6A
His6-HA-CaM (3621)	Bacterial expression	His6 and HA tags at N-terminus	Human Calmodulin; See Shao and Hegde, 2011	6

Extended Experimental Procedures

Plasmids, antibodies, and proteins

A description of all plasmids is provided in Table S1, along with the figure panels in which each was used. Plasmids for in vitro translation (IVT) in reticulocyte lysate (RRL) were based on the SP64 vector (Promega). Plasmids for IVT in the PURE system (Shimizu and Ueda, 2010) were based on the PURExpress DHFR Control Template provided by New England Biolabs. The DHFR open reading frame was replaced with the desired open reading frames. Plasmids for substrate expression in mammalian cells were based on either pCDNA3.1 (Invitrogen) or pEGFP (originally from Clontech) vectors, both of which use the CMV promoter.

Plasmid pX330 (Addgene 42230; Ran et al., 2013) was modified to remove the Cas9 expression cassette. This was then used for cloning of the desired sgRNA (chosen using the CRISPR design tool at crispr.mit.edu) to make knockout cell lines using CRISPR as described below. For transfection, the relevant expression cassettes for sgRNAs were PCR amplified using primers GAGGGCCTATTTCCCATGATTCC and CGGGCCATTTACCGTAAGTTAT and purified. The expression plasmid for FLAG-Cas9 was constructed by taking the FLAG-Cas9 open reading frame from plasmid pX330 (Ran et al., 2013) and cloned into the pCDNA5-Frt-TO vector. Myc-UBQLN1, used for rescue of the UBQLN knockout cell line, was cloned into the pCDNA5-Frt-TO vector.

The highly homologous UBQLN1 and UBQLN2 were detected simultaneously in blots using anti-UBQLN2 (from Sigma, clone 5F5, used at 1:1000 dilution). Control experiments using recombinant UBQLN1 and UBQLN2 verified that this antibody detects both antigens (data not shown). Anti-UBQLN4 was from Abcam (ab106443) and used at 1:1000 dilution. Rabbit polyclonal antibodies to Bag6, TRC40, SGTA, TRAPα, GFP, RFP have been described (Fons et al., 2003; Mariappan et al., 2010; Hessa et al., 2011). Anti-HA used for IPs and blots (e.g., Fig. 2A) was raised in rabbits against the KLH-HA peptide conjugate. Anti-L9 ribosomal protein was from Santa Cruz Biotech (T-17). The anti-Myc epitope tag (clone 9E10) was produced as culture supernatant from the corresponding hybridoma. Anti-HA was from Covance (Clone 16B12). Antibodies to proteasomal proteins Rpt5 and α7 were from Abcam (ab22635) and Enzo Life Sciences (PW8110), respectively. Anti-Tom20 was from Santa Cruz (FL-145). Anti-FLAG-M2 affinity resin and anti-FLAG-M2 antibody were from Sigma. GFP-trap was from Chromtek. Additional commercial antibodies used in Fig. 4A were: Hsc/Hsp70 (AssayDesigns, SPA-822), Hsp60 (Abcam ab46798), ClpP (Abcam ab124822), Actin-HRP (Sigma A3854), OxPhos cocktail (Abcam ab110411).

Recombinant His6-Ubiquitin, GST-Ube1 (E1 enzyme), and UbcH5a (E2 enzyme) were from Boston Biochem. Human UBQLN1 was tagged at the N-terminus with either a His6 tag or a tandem His6-3C-FLAG tag (where 3C is the cleavage site for 3C protease) and cloned into the pCOLA duet vector. The plasmids were introduced into BL21(DE3) LOBSTR strain of *E. coli* (Andersen et al., 2013), and induced with 0.1 mM IPTG at 16°C overnight. The cells were sedimented and washed in lysis buffer (PBS supplemented with 300 mM NaCl and 10 mM imidazole), resuspended in lysis buffer, and disrupted by sonication. The insoluble material was removed by centrifugation, and the soluble fraction was adjusted to 0.1% Triton X-100 before passing over a column of Co^{2+}-charged chelating sepharose (GE Biosciences). The column was washed with PBS containing 0.1% Triton X-100 and 500 mM NaCl, then extensively in PBS

plus 500 mM NaCl without detergent, and eluted with PBS plus 150 mM NaCl and 200 mM imidazole. The His tag on His-3C-Flag-UBQLN1 was cleaved by 3C protease, and the tag, protease, and uncleaved products were removed by passing over Co^{2+}-charged chelating sepharose resin. Recombinant TRC40, SGTA, and Bag6 were produced as previously described (Rodrigo-Brenni et al., 2014; Mateja et al., 2015).

Cell culture
HeLa and Flp-in T-Rex HEK293 cells (Invitrogen) were cultured in DMEM supplemented with 10% fetal bovine serum, 50 µg /ml penicillin/streptomycin in a humidified 5% CO_2 incubator. HEK293 Flp-in T-Rex cells were maintained in the presence of 15 µg/ml blasticidin and 100 µg/ml zeocin. Flag-Cas9, UBQLN triple knockout and UBQLN1 rescued cells (produced as described below) were maintained in the presence of 10 µg/ml blasticidin and 100 µg/ml hygromycin. 10 ng/ml doxycycline was used for induction of the integrated gene at the FRT site. Cell growth assay (Fig. 4C) was performed by passaging the cells into replicate 24 well plates and viable cells counted by trypan blue staining on each day for four days. For Fig. 4D, one hundred cells were passaged into 6-well dishes, cultured for 14 days, fixed in 3.7% formaldehyde in PBS for 30 min, and stained with 0.5% crystal violet in 10% methanol for 20 min. After washing in water, the cells were dried and photographed.

Microscopy
Images in Fig. 3C, 3F, and S5B, were acquired on a Nikon TE2000 fluorescence microscope with a 100x/1.49NA Oil objective. Fig. 3C and S5B show epifluorescence images. For Figure 3F, the specimen was imaged as a z-stack, deconvoluted using Huygens Software by Scientific Volume Imaging, and displayed as a maximum intensity projection with Fiji software. For Fig. 3F and S5B, samples were simply fixed and the fluorescent proteins imaged, while for Fig. 3C the sample was fixed, permeabilized, stained using anti-Myc antibody, and mounted prior to imaging.

Pulse-chase analysis
Analysis of cultured cells by pulse-chase labeling was essentially as described before (Ashok and Hegde, 2009, and references therein). Cells were typically transfected 24 h before initiating the experiment. Prior to labeling, cells were starved for 15 min at 37°C in media lacking methionine. Labeling was initiated by addition of ^{35}S-methionine to a final concentration of between 100 and 200 µCi per ml, depending on the experiment. After the intended labeling period, excess unlabeled methionine (to 2 mM final concentration) was added to the media, immediately after which the pulse labeled sample (i.e., the 0 min chase time point) was harvested. Subsequent time points were harvested in the same way (see below). Where indicated, a mixture of CCCP and Valinomycin (2 µM and 1 µM final concentrations, respectively) was included throughout (beginning with the starvation) to acutely inhibit mitochondrial import. Where MG132 was used for proteasome inhibition, it was added to a final concentration of 10 µM 90 min prior to beginning the starvation and maintained throughout the pulse and chase.

Cells were harvested in different ways depending on the downstream application. For immunoprecipitation of labeled products (e.g., Fig. 2A, 2B, S3B, and S3D), media was removed from the cells and they were directly solubilized in 1% SDS, 0.1 M Tris, pH 8. The samples were heated to 95°C, vortexed extensively and/or passed through narrow gauge needles to shear the

DNA, and cooled before dilution in immunoprecipitation (IP) buffer (150 mM NaCl, 50 mM Hepes, pH 7.4, 1% Triton X-100). For analysis of solubility (Fig. 2D), media was removed from the cells and the cells lysed in ice cold lysis buffer (150 mM NaCl, 50 mM Hepes, pH 7.4, 1% Triton X-100). The cell lysate was then passed 10 times through a 26 gauge needle. After centrifugation at 15,000 rpm for 30 min at 4°C, the supernatant was reserved in a separate tube and the pellet was dissolved directly in 1% SDS, 0.1 M Tris, pH 8.0. The genomic DNA for the pellet fraction was sheared by boiling and repeated vortexing, and the supernatant and pellet fractions analyzed either directly for autoradiography, subjected to immunoprecipitation, or subjected to immunoblotting.

In vitro transcription and translation

IVT reactions in the RRL system were performed by minor modifications of published methods (Sharma et al., 2010). SP6-based transcription utilized PCR products as templates and were for 1 hour at 37°C. Translation utilized RRL, phenyl-depleted RRL (Fig. S2E), DEAE-fractionated RRL (Fig. 4B), or phenyl-depleted and DEAE fractionated RRL (Fig. 7C) as previously described (Sharma et al., 2010; Hessa et al., 2011; Shao and Hegde, 2011). Where indicated in the Figure legends, recombinant proteins were added to the translation reactions, typically to 1 μM final concentration. Reactions were typically for 30 min at 32°C.

IVT in the PURE system was essentially as described (Shimizu and Ueda, 2010) and utilized either the PURExpress Kit (NEB) or in-house reagents prepared according to Shimizu and Ueda (2010). Where indicated, ^{35}S methionine and the indicated recombinant chaperone (at 15 μM final concentration) were included. Translation reactions were for 2 hours at 37°C. For site-specific photo-crosslinker incorporation (Fig. 6), the open reading frame was modified to have the UAG amber codon at the desired location and the UAA codon at the desired termination site. Translation reactions were supplemented with total tRNA isolated from *E. coli* expressing the amber suppressor (final A260 of 25), recombinant synthetase for charging the suppressor tRNA (50 μg/ml; Chin et al., 2002), and BpF (100 μM). A series of control experiments verified that all three components were required to obtain read-through of amber codons (data not shown), as expected from the well-documented orthogonality of this system in *E. coli* (Chin et al., 2002).

Sucrose gradient fractionation

0.2 ml gradients were prepared in 7 x 20 mm centrifuge tubes (Beckman 343775) by successively layering 40 μl each of 25%, 20%, 15%, 10%, and 5% sucrose (w/v) in KHM buffer (110 mM KAc, 20 mM Hepes, pH 7.4, 2 mM $MgAc_2$) and allowed to diffuse for 1-2 h at 4°C. Translation reactions (either 20 μl of RRL translations or 10 μl PURE reactions diluted with 10 μl KHM buffer) were loaded on top of the gradients, and the samples centrifuged in a TLS-55 rotor (with appropriate tube adaptors) at 55,000 rpm for 2.3 hours at 4°C with the slowest acceleration and deceleration settings. Eleven 20 μl fractions were successively collected from the top and used for downstream assays as indicated in the individual figure legends. The eleventh fraction was mixed with any pelleted materials. In some experiments, 10-fold larger gradients were prepared in exactly the same way using 11 x 34 mm tubes (Seton 5011), but centrifuged at 55,000 rpm in the TLS-55 rotor for 5 h. When fractionated into eleven fractions, the migration of products corresponds precisely to that seen in the smaller volume gradients.

Affinity purification

Affinity purification of UBQLN-client complexes was performed in the absence of detergent, which potently disrupts the interaction. In vitro translation reactions prepared as indicated in the individual figure legends, were added to pre-washed Anti-FLAG-M2 agarose or GFP-trap (typically 10 μl resin volume) and gently rotated for 2 hours at 4°C. The beads were washed between 4 to 6 times with 1 ml KHM buffer each. In some experiments, the beads were transferred to a new tube after the penultimate wash to reduce background. Bound proteins were eluted with SDS-PAGE sample buffer, or in some cases (e.g., Fig. 1A, 4B, and 7A), the FLAG peptide (added to 0.2 mM for 30 min at 20°C).

For affinity purification of UBQLN-client complexes from cells (Fig. 2C), WT and Myc-UBQLN1 rescue cells transfected with ATP5G1-HA were employed. After pulse-labeling with ^{35}S-methionine for 30 min, one-fifth of the cells was set aside for denaturing IPs of the ATP5G1-HA client to verify equal levels of expression. The remaining cells were chilled rapidly to between 0-4°C on ice, sedimented, and flash-frozen on dry ice. All further manipulations carried out with samples on ice in a cold room to minimize the otherwise rapid dissociation of client from UBQLN1 (e.g., Fig. 6B). The cells were thawed by addition of ice-cold lysis buffer (125 mM KAc, 50 mM Hepes, pH 7.4), dispersed by passage 7-8 times through a 26 gauge needle, and lysed by passage three times through a 30 gauge needle. The insoluble material was removed by centrifugation for 10 min at maximum speed in a microcentrifuge, and the supernatant added to ~5 μl immobilized anti-Myc monoclonal antibody (clone 9E10). After binding for 2 h at 4°C, the resin was washed three times in 80 volumes lysis buffer each, transferred to new tubes, and washed once more. After removing residual lysis buffer, the clients were eluted on ice with 1% Triton X-100, 150 mM NaCl, and 50 mM Hepes, pH 7.4. After removing the eluate, the resin was eluted a second time with SDS-PAGE sample buffer. One half of the Triton X-100 eluate was then subjected to re-immunoprecipitation with anti-HA resin after denaturation in SDS. Aliquots of all fractions and IPs were analyzed by SDS-PAGE and autoradiography.

Targeting assays in semi-intact cells

For microscopy-based targeting assays, HeLa cells grown on glass coverslips (13 mm diameter) were washed with ice-cold KHM buffer and treated with 50 μg/ml digitonin in KHM for 5 min at 4°C. After washing once in KHM buffer, the coverslips were placed in a humidified chamber at 30°C and overlaid with either 30 μl of the RRL translation reaction or 1 μl of the PURE system reaction diluted to 30 μl with KHM buffer. PURE IVT reactions and sucrose gradient fractions were supplemented with 3% BSA (to minimize non-specific binding) and an ATP regeneration system. Incubation was for 15 min (for RRL IVT) or 30 min (for PURE IVT). The coverslips were then washed with PBS twice and fixed in 3.7% formaldehyde in PBS for 15 min at room temperature. Fixed cells were permeabilized with 0.1% Triton X-100 in PBS for 5 min, blocked with 10% fetal bovine serum in PBS for 30 min, and incubated with primary antibodies in blocking buffer for 1 hour. After washing with PBS three times, cells were incubated with AlexaFluor 488-conjugated goat anti-mouse IgG and/or AlexaFluor 564-conjugated goat anti-rabbit IgG secondary antibodies (Invitrogen) in blocking buffer for 60 min. Images were acquired on a confocal laser scanning microscope (LSM 780, Zeiss) using a 63×/1.42NA oil-immersion objective.

For targeting assays using sedimentation, HEK293 cells (from a 10 cm dish at 50% confluence) were detached by pipetting with PBS and transferred to a 1.5 ml microcentrifuge tube. After centrifugation at 5000 rpm for 2 min, the supernatant was removed and the cells were resuspended in 50 μg/ml digitonin in KHM buffer and incubated for 5 min at 4°C. The cells were washed with KHM buffer 2 times and the semi-intact cells were resuspended in 500 μl KHM at 4°C. Twenty μl of the semi-intact cells were mixed with 10 μl of the RRL-based translation reaction and incubated for 30 min at 32°C. The reactions were then centrifuged at 2,348 x g for 5 min. The supernatant was saved and the pellet was washed with KHM 2 times, sedimenting the cells each time at 9,391 x g for 5 min. The cell fraction was treated with 1 U DNAse per 30 μl to digest genomic DNA. The supernatant and cell fractions were denatured in SDS-PAGE sample buffer prior to downstream analysis.

In vitro import into isolated mitochondria

Isolation of yeast mitochondria was as described (Meisinger et al., 2006), using the BY4741 strain. Import assays contained a 10:1 volume ratio of RRL translation reaction to mitochondria (A_{280} of ~30). Incubation was at 25 °C for 15 or 30 min. Reactions were directly analyzed (for signal cleavage) or centrifuged through a 15% sucrose cushion in 100 mM KAc, 50 mM Hepes, pH 7.4, 2 mM $MgCl_2$, in a microcentrifuge at 4 °C for 15 min at maximum speed. Equal amounts of supernatant and pellet samples were then directly analyzed by SDS-PAGE and autoradiography.

Generation of UBQLN triple knockout cells

To facilitate CRISPR/Cas9 mediated gene disruption, we first prepared stable inducible FLAG-Cas9 expressing cells by targeting the FLAG-Cas9 expression cassette to the FRT locus. Briefly, FLAG-Cas9 in the pCDNA5 Frt-Tet-On plasmid was co-transfected with the FLP recombinase expressing plasmid pOG44 (Invitrogen) into HEK293 Flp-in TRex cells and positive integrants were selected by resistance to 100 μg/ml hygromycin. These cells were then co-transfected with purified PCR products containing the sgRNA expression cassettes to target UBQLN1 (CGAGAATAGCTCCGTCCAGC), UBQLN2 (CGCGGGAACTAACACTACCT), and UBQLN4 (CTCTTGATCACAGTTCAAAG). FLAG-Cas9 expression was induced with 10 ng/ml doxycycline for 5 days after sgRNA transfection. Single cell clones were isolated by dilution into 96-well plates followed by culturing for 2 weeks, and screened for successful knockout of all three UBQLNs by immunoblotting relative to serial dilutions of parental cells.

To generate the UBQLN1 rescue cell line, the FLAG-Cas9 at FRT site of the UBQLN triple KO cells was removed by transient expression of the FLP recombinase using the pOG44 vector and selected for resistance to Zeocin. The restored FRT site was then re-targeted with either the empty pCDNA5-FRT-TO vector or pCDNA5-FRT-TO containing the Myc-UBQLN1 open reading frame. After hygromycin selection, the cell line transfected with the empty vector was used as the UBQLN triple knockout cell line, while the cell line transfected Myc-UBQLN1 was used as the rescue cell line. Induction of the rescue cell line with 10 ng/ml doxycycline resulted in Myc-UBQLN1 expression at levels comparable to total UBQLN1/2/4 expression in the parental HEK293 cells.

Detergent solubility assay
Cells in each well of a 6 well plate were mechanically lysed in 100 μl of lysis buffer (150 mM NaCl, 50 mM Tris pH. 7.4, 1mM EDTA, 0.5% Triton, 0.5% Deoxycholate, 1x Complete protease inhibitor cocktail, and 1 mM PMSF) and incubated on ice for 30 min. After centrifugation at 15,000 rpm for 30 min at 4°C, the supernatant was reserved in a separate tube and the pellet was washed once with lysis buffer, recovered by centrifugation, and resuspended in 100ul lysis buffer. Both the soluble and insoluble fractions were denatured in SDS-PAGE sample buffer for downstream analysis. Where necessary, the genomic DNA in the insoluble fraction was sheared by passing it several times through a 30-gauge needle. For Fig. 3D, S5A, and S5E, the cells were lysed in 150 mM NaCl, 50 mM Hepes, pH 7.4, 1% Triton X-100 and passed 10 times through a 26 gauge needle. After centrifugation at 15,000 rpm for 30 min at 4°C, the supernatant was reserved in a separate tube and the pellet was dissolved directly in SDS-PAGE sample buffer. The genomic DNA in was sheared by boiling and repeated vortexing.

Flow cytometry analysis
HEK293 cells transfected as indicated in the individual figure legends were recovered by detachment from the dish with EDTA and gentle pipetting. The cells were sedimented (5000 rpm for 2 min at 4°C) and resuspended in 10% fetal bovine serum in PBS for flow cytometry analysis using a Becton Dickinson LSRII analyzer. Sorting of aggregate-containing from aggregate-lacking cells was performed as described previously (Ramdzan et al., 2012) using a Sony iCyt Synergy sorter.

Analysis of ubiquitination
In experiments where ubiquitination in translation reactions was assessed (Fig. 4C, 5B, and S1C), 10 μM His-tagged Ubiquitin was added to the reaction. Ubiquitinated products were selectively recovered via the His tag by binding to Co^{2+}-charged chelating sepharose as described (Hessa et al., 2011). In reactions where ubiquitination was performed on affinity purified products (Fig. 4B) or PURE IVT reactions (Fig. S6A), the samples were supplemented with one-fourth volume of pre-assembled 4X Ubiquitination mix prepared by mixing 40 μM His-Ubiquitin, 0.4 μM GST-Ube1 (E1 enzyme), 1.2 μM UbcH5a (E2 enzyme), and an energy regenerating system (4 mM ATP, 40 mM creatine phosphate, 160 μg/ml creatine kinase). Reactions were incubated for 30 min at 37°C before termination by boiling in 1% SDS. The denatured products were diluted 10-fold in 50 mM HEPES pH7.4, 100 mM NaCl, 1% TritonX100 and the ubiquitinated products recovered by binding to Co^{2+}-immobilized chelating sepharose.

Miscellaneous
In most cases, samples were analyzed on 10 or 12% Tris-Tricine gels. Equal loading for SDS-PAGE, IPs, and targeting assays into semi-intact cells was controlled as follows. For immunoblotting experiments, equal loading was ensured by two means. First, the protein concentrations of cell lysates were measured and equal amounts were used for downstream analysis. Second, the blots were always stained for total protein (shown in the case of Fig. 2E and 3D as examples), verifying equal loading and recovery. In experiments where radiolabeled products were analyzed, the gels were always stained with coomassie blue to verify equal loading of IVT products or cell lysate as appropriate. In immunoprecipitation or pulldown experiments, equal amounts of samples were used to program the IP reaction, and equal recovery during the IP was always verified by staining of the gel to visualize IgG (or free ubiquitin in the

case of ubiquitin-pulldowns). For the pulse-chase experiment, small aliquots of the lysates for all samples were analyzed separately to verify equal rates of label incorporation. In instances where the IPs were eluted with peptide (e.g., Fig. 1A), the remaining beads were boiled and analyzed to verify equal recovery of IgG. For import reactions into semi-permeabilized cells, the amounts of translation products were quantified by using a small amount of ^{35}S-methionine as a tracer, and equal amounts of translated product were used in the assay.

Supplemental References

Andersen, K.R., Leksa,N.C., Schwartz, T.U. (2013) Optimized E. coli expression strain LOBSTR eliminates common contaminants from His-tag purification. Proteins *81*, 1857-61.

Fons, R.D., Bogert, B.A., Hegde, R.S. (2003) Substrate-specific function of the translocon-associated protein complex during translocation across the ER membrane. J Cell Biol.*160*, 529-39.

Meisinger, C., Pfanner, N., Truscott, K.N. (2006) Isolation of yeast mitochondria. Methods Mol Biol. *313*, 33-9.
